# Supplementary material for: Relationship between primary school healthy eating and physical activity promoting environments and children’s dietary intake, physical activity and weight status: a longitudinal study in the West Midlands, UK
Source: BMJ Open. 2020 Dec 22;10(12):e040833. doi: 10.1136/bmjopen-2020-040833 (PMC7757477; doi:10.1136/bmjopen-2020-040833)
Supplement: Supplementary data [file bmjopen-2020-040833supp001.pdf]

Relationship between primary school healthy eating and physical activity promoting environments and children's dietary intake, physical activity and weight status: a longitudinal study in the West Midlands, United Kingdom

Supplementary file

Table 1: School environment and policy scoring

Description of variable	Score allocation
**Healthy eating score**	
School has a policy regarding HE	0 = No<br>2 = Yes
HE is promoted in school curriculum	0 = No<br>2 = Yes
School cookery club to promote HE	0 = No<br>2 = Yes
Involve parents in the promotion of HE	0 = No<br>2 = Yes
Provide a range of ways to promote HE	Split data into tertiles<br>0 = < 5<br>1 = 5 ≤ x < 7<br>2 = ≥ 7
Drinking water is provided throughout the day	0 = No<br>2 = Yes
Breakfast club is available for students	0 = No<br>2 = Yes
Food available at break deemed as healthy	0 = No†<br>2 = Yes†
Proportion of children choosing to eat school lunches	Split data into tertiles<br>0 = < 50%<br>1 = 50% ≤ x < 60%<br>2 = ≥ 60% of children
Governors support HE in school *	0.33 = Weakly support<br>0.67 = Support<br>1.00 = Strongly support
The Senior Leadership Team support HE in school *	0.33 = Weakly support<br>0.67 = Support<br>1.00 = Strongly support
Staff support HE in school *	0.33 = Weakly support<br>0.67 = Support<br>1.00 = Strongly support
School council support HE in school *	0.33 = Weakly support<br>0.67 = Support<br>1.00 = Strongly support
Pupils support HE in school *	0.33 = Weakly support<br>0.67 = Support<br>1.00 = Strongly support
Parents support HE in school *	0.33 = Weakly support<br>0.67 = Support<br>1.00 = Strongly support
**Physical activity score**	
School has a policy regarding PA	0 = No<br>2 = Yes
School has a PA coordinator	0 = No<br>2 = Yes
Staff are trained regarding PA	0 = No<br>2 = Yes

Garden EM, *et al. BMJ Open* 2020; 10:e040833. doi: 10.1136/bmjopen-2020-040833

Description of variable	Score allocation
School has sport partnerships and community links	0 = No<br>2 = Yes
PA is promoted in the school curriculum	0 = No<br>2 = Yes
Involve parents in the promotion of PA	0 = No<br>2 = Yes
Have a walk to school campaign	0 = No<br>2 = Yes
Provide a range of ways to promote PA	Split data into tertiles<br>0 = < 5<br>1 = 5 ≤ x < 7<br>2 = ≥ 7
PE is taught by a specialist	0 = no<br>2 = yes
Range of activities offered in PE meet Department of Education recommendations	0 = no<br>2 = yes
Schools allocating two or more hours to PE per week	0 = for none of the school<br>1 = for some of the years<br>2 = for the whole school
Offer a variety of PA opportunities, other than PE	Split data into tertiles<br>0 = 0<br>1 = 1<br>2 = ≥ 2
Have a range of play equipment to be used	Split data into tertiles<br>0 = < 5<br>1 = 5<br>2 = ≥ 6
Provide a range of physically active/ sports clubs	Split data into tertiles<br>0 = < 4<br>1 = 4 ≤ x < 6<br>2 = ≥ 6
Governors support PA in school *	0.33 = Weakly support<br>0.67 = Support<br>1.00 = Strongly support
The Senior Leadership Team support PA in school *	0.33 = Weakly support<br>0.67 = Support<br>1.00 = Strongly support
Staff support PA in school *	0.33 = Weakly support<br>0.67 = Support<br>1.00 = Strongly support
School council support PA in school *	0.33 = Weakly support<br>0.67 = Support<br>1.00 = Strongly support
Pupils support PA in school *	0.33 = Weakly support<br>0.67 = Support<br>1.00 = Strongly support
Parents support PA in school *	0.33 = Weakly support<br>0.67 = Support<br>1.00 = Strongly support

* Given a maximum score of 1, to ensure that level of support by respective groups did not account for too great a proportion of overall score.
† No = Any food other than fruit, vegetables, milk and juice. Yes = Fruit, vegetables, milk and juice
HE, healthy eating. PA, physical activity. PE, physical education.

Garden EM, *et al. BMJ Open* 2020; 10:e040833. doi: 10.1136/bmjopen-2020-040833

Table 2: Multilevel models developed for body mass index z-score at the three study time points: all fixed effect covariates are presented

	**Baseline**		**Follow-up one**		**Follow-up two**	
	**B (95% CI)**	**p-value**	**B (95% CI)**	**p-value**	**B (95% CI)**	**p-value**
**Trial arm**	N/A	N/A	0.07 (-0.05, 0.19)	0.24	0.03 (-0.07, 0.14)	0.55
**Baseline zBMI**	N/A	N/A	0.95 (0.93, 0.98)	< 0.01	0.92 (0.89, 0.96)	< 0.01
**Index of Multiple Deprivation score**	0.004 (-0.001, 0.008)	0.09	<0.001 (-0.003, 0.002)	0.74	0.0004 (-0.003, 0.003)	0.80
**Female (reference male)**	-0.08 (-0.22, 0.05)	0.23	-0.11 (-0.18, -0.05)	< 0.01	-0.16 (-0.24, -0.07)	< 0.01
**Ethnicity (reference White)**						
**Asian**	-0.27 (-0.44, -0.10)	< 0.01	0.10 (0.01, 0.20)	0.03	0.16 (0.05, 0.28)	0.01
**African Caribbean**	0.38 (0.11, 0.65)	0.01	0.08 (-0.06, 0.22)	0.29	0.01 (-0.18, 0.19)	0.96
**Other**	-0.02 (-0.23, 0.18)	0.83	0.01 (-0.09, 0.12)	0.78	0.04 (-0.10, 0.17)	0.61
**Age**	0.03 (-0.19, 0.25)	0.76	-0.02 (-0.12, 0.09)	0.73	0.02 (-0.12, 0.16)	0.80
**School environment category (reference = minimal health focus)**						
**PA focus**	-0.06 (-0.25, 0.14)	0.57	-0.06 (-0.22, 0.10)	0.48	-0.17 (-0.31, -0.03)	0.02
**HE focus**	-0.09 (-0.28, 0.09)	0.33	-0.02 (-0.18, 0.15)	0.86	-0.02 (-0.16, 0.13)	0.80
**Complete health focus**	0.04 (-0.14, 0.23)	0.64	0.07 (-0.09, 0.23)	0.41	-0.11 (-0.25. 0.03)	0.14

95% CI, 95% confidence interval. N/A, not applicable. zBMI, body mass index z-score. PA, physical activity. HE, healthy eating.

Garden EM, *et al. BMJ Open* 2020; 10:e040833. doi: 10.1136/bmjopen-2020-040833

Table 3: Multilevel models developed for waist circumference z-score at the three study time points: all fixed effect covariates are presented

	**Baseline**		**Follow-up one**		**Follow-up two**	
	**B (95% CI)**	**p-value**	**B (95% CI)**	**p-value**	**B (95% CI)**	**p-value**
**Trial arm**	N/A	N/A	-0.12 (-0.31, 0.07)	0.23	-0.09 (-0.25, 0.07)	0.27
**Baseline zWC**	N/A	N/A	0.90 (0.86, 0.94)	< 0.01	0.85 (0.80, 0.89)	< 0.01
**Index of Multiple Deprivation score**	0.002 (-0.003, 0.007)	0.35	-0.001 (-0.004, 0.003)	0.76	0.001 (-0.003, 0.005)	0.48
**Female (reference male)**	0.07 (-0.07, 0.21)	0.32	-0.06 (-0.15, 0.03)	0.17	0.11 (0.01, 0.21)	0.04
**Ethnicity (reference White)**						
**Asian**	-0.20 (-0.39, -0.01)	0.04	0.19 (0.06, 0.32)	< 0.01	0.21 (0.07, 0.36)	< 0.01
**African Caribbean**	0.33 (0.04, 0.62)	0.03	0.02 (-0.18, 0.21)	0.88	0.07 (-0.17, 0.31)	0.57
**Other**	0.004 (-0.21, 0.22)	0.97	0.06 (-0.08, 0.20)	0.41	0.05 (-0.18, 0.21)	0.58
**Age**	0.05 (-0.18, 0.29)	0.65	-0.06 (-0.21, 0.09)	0.44	0.05 (-0.13, 0.22)	0.58
**School environment category (reference = minimal health focus)**						
**PA focus**	-0.10 (-0.36, 0.16)	0.44	0.04 (-0.22, 0.30)	0.75	-0.15 (-0.36, 0.06)	0.16
**HE focus**	-0.24 (-0.48, 0.01)	0.06	0.19 (-0.07, 0.45)	0.14	0.07 (-0.15, 0.29)	0.55
**Complete health focus**	0.10 (-0.14, 0.34)	0.40	-0.01 (-0.26, 0.24)	0.95	-0.18 (-0.38, 0.03)	0.09

95% CI, 95% confidence interval. N/A, not applicable. zWC, waist circumference z-score. PA, physical activity. HE, healthy eating.

Garden EM, *et al. BMJ Open* 2020; 10:e040833. doi: 10.1136/bmjopen-2020-040833

Table 4: Multilevel models developed for dietary total energy intake at the three study time points: all fixed effect covariates are presented

	Baseline		Follow-up one		Follow-up two	
	**B (95% CI)**	**p-value**	**B (95% CI)**	**p-value**	**B (95% CI**	**p-value**
**Trial arm**	N/A	N/A	18.5 (-364.3, 401.4)	0.92	71.4 (-431.0, 573.7)	0.78
**Baseline dietary total energy intake (kJ/ 24 hours)**	N/A	N/A	0.02 (0.01, 0.06)	0.19	0.06 (0.02, 0.11)	< 0.01
**Index of Multiple Deprivation score**	13.7 (-5.1, 32.5)	0.15	2.7 (-7.6, 13.0)	0.61	10.1 (-2.7, 22.8)	0.12
**Female (reference male)**	-586.9 (-1028.2, -145.6)	0.01	-199.7 (-484.2, 84.8)	0.17	-38.9 (-376.8, 298.9)	0.82
**Ethnicity (reference White)**						
**Asian**	-38.4 (-699.0, 622.3)	0.91	-423.5 (-803.7, -43.2)	0.03	304.4 (-153.3, 762.0)	0.19
**African Caribbean**	439.2 (-518.6, 1397.0)	0.37	97.2 (-520.6, 715.1)	0.76	43.9 (-740.1, 827.9)	0.91
**Other**	319.6 (-382.5, 1021.7)	0.37	-505.4 (-944.4, -66.3)	0.02	-120.8 (-654.6, 412.9)	0.66
**Age**	621.5 (-119.3, 1362.3)	0.10	423.3 (-49.1, 895.6)	0.08	353.6 (-208.3, 915.6)	0.22
**School environment category (reference = minimal health focus)**						
**PA focus**	-903.5 (-2402.7, 595.6)	0.24	433.7 (-70.5, 937.9)	0.09	33.2 (-614.5, 681.0)	0.92
**HE focus**	-847.8 (-2255.8, 560.3)	0.24	-63.7 (-598.0, 470.5)	0.82	260.8 (-443.1, 964.7)	0.47
**Complete health focus**	-399.3 (-1801.3, 1002.6)	0.58	32.8 (-464.7, 530.3)	0.90	171.3 (-466.4, 809.1)	0.60

95% CI, 95% confidence interval. N/A, not applicable. PA, physical activity. HE, healthy eating.

Garden EM, *et al. BMJ Open* 2020; 10:e040833. doi: 10.1136/bmjopen-2020-040833

Table 5: Multilevel models developed for fruit and vegetable intake at the three study time points: all fixed effect covariates are presented

	**Baseline**		**Follow-up one**		**Follow-up two**	
	**B (95% CI)**	**p-value**	**B (95% CI)**	**p-value**	**B (95% CI)**	**p-value**
**Trial arm**	N/A	N/A	9.6 (-20.5, 39.6)	0.53	-5.5 (-50.3, 39.2)	0.81
**Baseline fruit and vegetable intake (g/ 24 hours)**	N/A	N/A	0.17 (0.12, 0.22)	< 0.01	0.08 (0.02, 0.15)	0.01
**Index of Multiple Deprivation score**	0.31 (-0.72, 1.4)	0.55	-0.58 (-1.4, 0.20)	0.15	-0.66 (-1.7, 0.43)	0.24
**Female (reference male)**	11.8 (-13.0, 36.7)	0.35	36.9 (15.7, 58.1)	< 0.01	12.4 (-15.5, 40.4)	0.38
**Ethnicity (reference White)**						
**Asian**	17.0 (-19.9, 53.8)	0.37	9.6 (-19.1, 38.2)	0.51	46.5 (7.9, 85.0)	0.02
**African Caribbean**	10.8 (-43.0, 64.6)	0.69	64.8 (18.6, 111.0)	0.01	33.2 (-32.1, 98.4)	0.32
**Other**	41.6 (2.2, 81.0)	0.04	26.7 (-6.2, 59.6)	0.11	60.6 (16.1, 105.1)	0.01
**Age**	38.4 (-3.4, 80.1)	0.07	31.5 (-3.7, 66.7)	0.08	16.2 (-30.4, 62.7)	0.50
**School environment category (reference = minimal health focus)**						
**PA focus**	-57.8 (-135.5, 19.8)	0.14	11.8 (-27.9, 51.5)	0.56	-16.0 (-73.9, 41.9)	0.59
**HE focus**	-31.6 (-104.7, 41.4)	0.40	3.0 (-38.6, 44.7)	0.89	-4.4 (-66.9, 58.0)	0.89
**Complete health focus**	-7.3 (-80.0, 65.4)	0.84	27.1 (-11.9, 66.0)	0.17	8.6 (-48.1, 65.3)	0.77

95% CI, 95% confidence interval. N/A, not applicable. PA, physical activity. HE, healthy eating.

Garden EM, *et al. BMJ Open* 2020; 10:e040833. doi: 10.1136/bmjopen-2020-040833

Table 6: Multilevel models developed for physical activity energy expenditure at the three study time points: all fixed effect covariates are presented

	Baseline		Follow-up one		Follow-up two	
	**B (95% CI)**	**p-value**	**B (95% CI)**	**p-value**	**B (95% CI)**	**p-value**
**Trial arm**	N/A	N/A	0.25 (-4.6, 5.1)	0.92	-1.7 (-6.0, 2.6)	0.44
**Baseline PAEE (kJ/kg body weight/ 24 hours)**	N/A	N/A	0.53 (0.45, 0.60)	< 0.01	0.43 (0.36, 0.51)	< 0.01
**Index of Multiple Deprivation score**	0.09 (0.01, 0.19)	0.09	-0.10 (-0.22, 0.02)	0.09	-0.02 (-0.13, 0.10)	0.79
**Female (reference male)**	-14.1 (-16.9, -11.2)	< 0.01	-4.9 (-8.2, -1.6)	< 0.01	-7.0 (-10.4, -3.5)	< 0.01
**Ethnicity (reference White)**						
**Asian**	-3.8 (-7.7, 0.13)	0.06	-3.7 (-8.0, 0.62)	0.09	-7.9 (-12.1, -3.6)	< 0.01
**African Caribbean**	-17.8 (-23.8, -11.8)	< 0.01	-6.9 (-14.0, 0.18)	0.06	-12.8 (-21.0, -4.6)	< 0.01
**Other**	-4.1 (-8.4, 0.24)	0.06	-2.4 (-7.0, 2.3)	0.32	-4.4 (-9.4, 0.73)	0.09
**Age**	0.57 (-4.2, 5.3)	0.81	2.0 (-3.2, 7.3)	0.46	-6.6 (-12.1, -1.1)	0.02
**School environment category (reference = minimal health focus)**						
**PA focus**	-6.0 (-11.7, -0.28)	0.04	-0.79 (-7.2, 5.7)	0.81	1.9 (-3.8, 7.6)	0.51
**HE focus**	-6.3 (-11.8, -0.86)	0.02	3.8 (-3.1, 10.7)	0.28	3.5 (-2.7, 9.7)	0.27
**Complete health focus**	-6.3 (-11.6, -1.0)	0.02	-2.6 (-8.8, 3.6)	0.42	5.3 (0.04, 10.6)	0.05

95% CI, 95% confidence interval. N/A, not applicable. PAEE, physical activity energy expenditure. PA, physical activity. HE, healthy eating.

Garden EM, *et al. BMJ Open* 2020; 10:e040833. doi: 10.1136/bmjopen-2020-040833

Table 7: Multilevel models developed for estimated time spent in moderate to vigorous physical activity at the three study time points: all fixed effect covariates are presented

	Baseline		Follow-up one		Follow-up two	
	**B (95% CI)**	**p-value**	**B (95% CI)**	**p-value**	**B (95% CI)**	**p-value**
**Trial arm**	N/A	N/A	0.57 (-13.5, 14.6)	0.94	3.8 (-7.1, 14.7)	0.49
**Baseline estimated time spent in MVPA (min/ 24 hours)**	N/A	N/A	0.21 (0.11, 0.31)	< 0.01	0.13 (0.06, 0.20)	< 0.01
**Index of Multiple Deprivation score**	0.26 (0.03, 0.50)	0.03	-0.18 (-0.52, 0.15)	0.28	0.04 (-0.29, 0.20)	0.72
**Female (reference male)**	-13.6 (-19.6, -7.6)	< 0.01	-13.6 (-22.4, -4.7)	< 0.01	-13.6 (-19.6, -7.6)	< 0.01
**Ethnicity (reference White)**						
**Asian**	-3.0 (-11.6, 5.5)	0.49	-5.8 (-18.0, 6.4)	0.35	-12.5 (-20.8, -4.2)	< 0.01
**African Caribbean**	2.6 (-10.2, 15.5)	0.69	-3.1 (-23.1, 17.0)	0.77	-7.3 (-22.3, 7.6)	0.34
**Other**	5.8 (-3.4, 15.1)	0.22	-3.6 (-16.8, 9.6)	0.59	-3.4 (-13.0, 6.1)	0.48
**Age**	-0.62 (-10.7, 9.4)	0.90	8.4 (-6.4, 23.2)	0.27	-9.1 (-19.1, 1.0)	0.08
**School environment category (reference = minimal health focus)**						
**PA focus**	-4.2 (-18.5, 10.1)	0.57	-3.7 (-22.7, 15.2)	0.70	3.8 (-10.5, 18.2)	0.60
**HE focus**	-13.2 (-26.8, 0.47)	0.06	-0.80 (-20.9, 19.3)	0.94	0.78 (-14.5, 16.1)	0.92
**Complete health focus**	-14.1 (-27.4, -0.75)	0.04	-6.4 (-24.7, 11.8)	0.49	7.2 (-6.4, 20.9)	0.30

95% CI, 95% confidence interval. N/A, not applicable. MVPA, moderate to vigorous physical activity. PA, physical activity. HE, healthy eating.

Garden EM, *et al. BMJ Open* 2020; 10:e040833. doi: 10.1136/bmjopen-2020-040833

Table 8: Repeated measures models for outcomes of body mass index z-score, waist circumference z-score, dietary total energy intake, fruit and vegetable intake, physical activity energy expenditure and time spent in moderate to vigorous physical activity: all fixed covariates are presented

	**Outcome**											
	**zBMI**		**zWC**		**Dietary total energy intake (kJ/24 hrs)**		**Fruit and vegetable intake (g/24 hrs)**		**PAEE (kJ/kg body weight/24 hrs)**		**Estimated time in MVPA (min/24 hrs)**	
	**B (95% CI)**	**p-value**	**B (95% CI)**	**p-value**	**B (95% CI)**	**p-value**	**B (95% CI)**	**p-value**	**B (95% CI)**	**p-value**	**B (95% CI)**	**p-value**
**Trial arm**	-0.04 (-0.19, 0.10)	0.55	-0.11 (-0.28, 0.05)	0.17	322.7 (-120.8, 766.2)	0.15	20.1 (-11.4, 51.6)	0.21	-1.9 (-5.1, 1.4)	0.26	0.27 (-7.5, 8.1)	0.95
**Time point (reference baseline)**												
**Follow-up 1**	0.11 (0.08, 0.15)	<0.01	0.25 (0.20, 0.29)	<0.01	-229.2 (-491.6, 33.2)	0.09	-52.5 (-68.4, -36.6)	<0.01	-2.7 (-4.4, -1.0)	<0.01	10.4 (6.0, 14.8)	<0.01
**Follow-up 2**	0.19 (0.15, 0.22)	<0.01	0.30 (0.25, 0.35)	<0.01	445.1 (174.9, 715.2)	<0.01	-29.5 (-45.9, -13.1)	<0.01	-15.1 (-16.9, -13.3)	<0.01	-16.5 (-21.1, -11.8)	<0.01
**Index of Multiple Deprivation score**	0.004 (-0.001, 0.01)	0.12	0.003 (-0.002, 0.01)	0.26	10.6 (1.2, 19.9)	0.02	0.05 (-0.62, 0.71)	0.89	0.0002 (-0.8, 0.8)	0.99	0.04 (-0.14, 0.21)	0.67
**Female (reference male)**	-0.15 (-0.29, -0.01)	0.03	0.06 (-0.07, 0.20)	0.36	-316.1 (-554.0, -87.1)	0.01	20.3 (4.1, 36.5)	0.01	-13.3 (-15.6, -11.0)	<0.01	-14.3 (-18.7, -9.9)	<0.01
**Ethnicity (reference White)**												
**Asian**	-0.19 (-0.36, -0.02)	0.03	-0.09 (-0.26, 0.09)	0.34	-18.8 (-349.0, 311.5)	0.91	19.9 (-3.6, 43.3)	0.10	-6.7 (-9.8, -3.5)	<0.01	-8.1 (-14.3, -1.9)	0.01
**African Caribbean**	0.38 (0.11, 0.65)	0.01	0.31 (0.03, 0.59)	0.03	198.2 (-299.7, 696.0)	0.44	26.1 (-8.9, 61.0)	0.14	-16.9 (-21.9, -12.0)	<0.01	0.80 (-8.8, 10.4)	0.87
**Other**	-0.004 (-0.21, 0.20)	0.97	0.02 (-0.19, 0.23)	0.87	-38.9 (-401.4, 323.5)	0.83	41.4 (15.8, 67.1)	<0.01	-5.7 (-9.3, -2.2)	<0.01	0.52 (-6.3, 7.3)	0.88
**Age**	0.03 (-0.19, 0.25)	0.49	0.11 (-0.12, 0.33)	0.35	529.3 (148.7, 909.9)	0.01	37.9 (11.0, 64.9)	0.01	-0.70 (-4.6, 3.2)	0.73	-1.8 (-9.2, 5.5)	0.62
**School environment category (reference = minimal health focus)**												

	Outcome											
	**zBMI**		**zWC**		**Dietary total energy intake (kJ/24 hrs)**		**Fruit and vegetable intake (g/24 hrs)**		**PAEE (kJ/kg body weight/24 hrs)**		**Estimated time in MVPA (min/24 hrs)**	
	**B (95% CI)**	**p-value**	**B (95% CI)**	**p-value**	**B (95% CI)**	**p-value**	**B (95% CI)**	**p-value**	**B (95% CI)**	**p-value**	**B (95% CI)**	**p-value**
**PA focus**	-0.10 (-0.29, 0.10)	0.32	-0.11 (-0.32, 0.11)	0.33	-220.9 (-815.1, 373.4)	0.47	-22.6 (-64.9, 19.6)	0.29	-2.8 (-7.2, 1.6)	0.21	-1.4 (-11.9, 9.1)	0.80
**HE focus**	-0.07 (-0.27, 0.13)	0.47	-0.14 (-0.37, 0.08)	0.21	-483.8 (-1087.4, 119.9)	0.12	-21.6 (-64.4, 21.2)	0.32	-0.83 (-5.3, 3.7)	0.72	-5.0 (-15.7, 5.7)	0.36
**Complete health focus**	0.07 (-0.12, 0.26)	0.49	0.07 (-0.14, 0.28)	0.52	-200.0 (-773.9, 373.8)	0.49	5.16 (-35.6, 45.9)	0.80	-3.0 (-7.2, 1.2)	0.16	-6.4 (-16.5, 3.7)	0.21

zBMI, body mass index z-score. zWC, waist circumference z-score. PAEE, physical activity energy expenditure. MVPA, moderate to vigorous physical activity. 95% CI, 95% confidence interval. PAEE, physical activity energy expenditure. PA, physical activity. HE, healthy eating.

Garden EM, *et al. BMJ Open* 2020; 10:e040833. doi: 10.1136/bmjopen-2020-040833
