# Supplementary material for: Relationship between primary school healthy eating and physical activity promoting environments and children’s dietary intake, physical activity and weight status: a longitudinal study in the West Midlands, UK
Source: BMJ Open. 2020 Dec 22;10(12):e040833. doi: 10.1136/bmjopen-2020-040833 (PMC7757477; doi:10.1136/bmjopen-2020-040833)
Supplement: Author's manuscript [file bmjopen-2020-040833.draft_revisions.pdf]

BMJ Open is committed to open peer review. As part of this commitment we make the peer review history of every article we publish publicly available.

When an article is published we post the peer reviewers' comments and the authors' responses online. We also post the versions of the paper that were used during peer review. These are the versions that the peer review comments apply to.

The versions of the paper that follow are the versions that were submitted during the peer review process. They are not the versions of record or the final published versions. They should not be cited or distributed as the published version of this manuscript.

BMJ Open is an open access journal and the full, final, typeset and author-corrected version of record of the manuscript is available on our site with no access controls, subscription charges or pay-per-view fees (http://bmjopen.bmj.com).

If you have any questions on BMJ Open's open peer review process please email info.bmjopen@bmj.com

Relationship between primary school healthy eating and physical activity promoting environments and children's dietary intake, physical activity and weight status: a longitudinal study in the West Midlands, United Kingdom

Journal:	*BMJ Open*
Manuscript ID	bmjopen-2020-040833
Article Type:	Original research
Date Submitted by the Author:	26-May-2020

Complete List of Authors:	Garden, Elizabeth; University of Birmingham<br>Pallan, Miranda; University of Birmingham, Public Health, Epidemiology and Biostatistics<br>Clarke, Joanne; University of Birmingham, Institute of Applied Health Research<br>Griffin, Tania ; University of Bath,<br>Hurley, Kiya; University of Birmingham, Cancer Research UK Clinical Trials Unit<br>Lancashire, Emma; University of Birmingham, Public Health, Epidemiology and Biostatistics<br>Passmore, Sandra; Services for Education Ltd,<br>Adab, Peymane; University of Birmingham, Public Health

Keywords:	PUBLIC HEALTH, NUTRITION & DIETETICS, PREVENTIVE MEDICINE

SCHOLARONE™
Manuscripts

I, the Submitting Author has the right to grant and does grant on behalf of all authors of the Work (as defined in the below author licence), an exclusive licence and/or a non-exclusive licence for contributions from authors who are: i) UK Crown employees; ii) where BMJ has agreed a CC-BY licence shall apply, and/or iii) in accordance with the terms applicable for US Federal Government officers or employees acting as part of their official duties; on a worldwide, perpetual, irrevocable, royalty-free basis to BMJ Publishing Group Ltd ("BMJ") its licensees and where the relevant Journal is co-owned by BMJ to the co-owners of the Journal, to publish the Work in this journal and any other BMJ products and to exploit all rights, as set out in our licence.

The Submitting Author accepts and understands that any supply made under these terms is made by BMJ to the Submitting Author unless you are acting as an employee on behalf of your employer or a postgraduate student of an affiliated institution which is paying any applicable article publishing charge ("APC") for Open Access articles. Where the Submitting Author wishes to make the Work available on an Open Access basis (and intends to pay the relevant APC), the terms of reuse of such Open Access shall be governed by a Creative Commons licence – details of these licences and which Creative Commons licence will apply to this Work are set out in our licence referred to above.

Other than as permitted in any relevant BMJ Author's Self Archiving Policies, I confirm this Work has not been accepted for publication elsewhere, is not being considered for publication elsewhere and does not duplicate material already published. I confirm all authors consent to publication of this Work and authorise the granting of this licence.

For peer review only

Relationship between primary school healthy eating and physical activity promoting environments and children's dietary intake, physical activity and weight status: a longitudinal study in the West Midlands, United Kingdom

Mairenn Garden, Miranda Pallan, Joanne Clarke, Tania Griffin, Kiya Hurley, Emma Lancashire, Sandra Passmore, Peymane Adab

Affiliations:

Mairenn Garden: Student, University of Birmingham; Birmingham, United Kingdom

Miranda Pallan: Institute of Applied Health Research, University of Birmingham, Birmingham, UK

Joanne Clarke; Institute of Applied Health Research, University of Birmingham, Birmingham, UK

Tania Griffin: Department for Health, University of Bath; Bath, Uk

Kiya Hurley: Cancer Clinical Trials Unit, University of Birmingham; Birmingham, uk

Emma Lancashire: Institute of Applied Health Research, University of Birmingham, Birmingham, UK

Sandra Passmore: Services for Education; Birmingham, United Kingdom

Peymane Adab: Institute of Applied Health Research, University of Birmingham, Birmingham, UK

Corresponding author:

Miranda Pallan: Institute of Applied Health Research, Murray Learning Centre, University of Birmingham, Edgbaston, Birmingham, UK. B15 2TT

Email: M.J.Pallan@bham.ac.uk

Word count: 3,632 (excluding title page, abstract, references, figures and tables)

Abstract word count: 230

For peer review only

ABSTRACT

Objectives: This study aims to examine the association between health promoting environments in primary schools and child anthropometric, healthy eating and physical activity measures.

Design: Secondary data analysis.

Setting: 50 state-primary schools.

Participants: 1, 304 pupils who participated in the West Midlands ActiVe lifestyle and healthy Eating in School children (WAVES) childhood obesity prevention trial (2011-2015).

Outcome measures: School environment; was categorised according to questionnaire responses indicating their support for healthy eating and/or physical activity. Child measures; undertaken at three time-points (age five-to-six, seven-to-eight and eight-to-nine years), included: body mass index z-scores, (using a 24-hour food-tick-list) and physical activity (using an Actiheart monitor over 5 days).

Analyses: The associations between school health promotion environment categories and outcomes were explored through multilevel models at each time point.

Results: Data were available for 1,304 children. At age eight-to-nine years, children in ten schools with healthy eating and physical activity-supportive environments had a higher physical activity energy expenditure than those in 22 schools with non-supportive environments (mean difference=5.3kJ/kg body weight/24 hours, p=0.05). Children in eight schools with supportive physical activity environments had a lower body mass index z-score than those (n=22) in non-supportive environments (mean difference=-0.166, p=0.02). School health promoting environments were not significantly associated with dietary outcomes.

Conclusions: Health promoting school environments may positively influence physical activity and childhood obesity.

Trial registration: The WAVES trial was registered on 19/05/2010, registration number: ISRCTN97000586.

For peer review only - http://bmjopen.bmj.com/site/about/guidelines.xhtml

KEY WORDS

Obesity, Child, Primary school, Environment, Diet, Healthy eating, Physical activity

ARTICLE SUMMARY

Strengths and limitations of this study

- The study was conducted with a large ethnically and socio-economically diverse sample of children over three years.
- Outcomes were assessed by objective measures and validated instruments, using standardised procedures.
- The school questionnaire and scoring system used to assess school health promoting environments may not give a true representation of the extent to which schools promote health.
- Associations between weight, dietary and physical activity outcomes, and four categories of health promoting school were explored, but this does not fully take into account the large variation in environments seen across schools.
- The Child And Diet Evaluation Tool (CADET) only measured dietary intake over a single day, so may not be an accurate assessment of habitual intake.

For peer review only

For peer review only - http://bmjopen.bmj.com/site/about/guidelines.xhtml

BACKGROUND

Childhood obesity is a global public health crisis,[1] affecting countries of all income levels.[2] Despite a recent plateau, its prevalence remains high in England, and a substantial increase in excess weight prevalence is seen across the primary school years.[3] Approximately 23% of children age four-to-five years are living with overweight and obesity, and this rises to 34% by age ten-to-11 years.[3] Excess weight in childhood is associated with a range of physical, psychological and social challenges,[4] and is likely to persist into adulthood.[5] Excess weight also results in significant healthcare costs, estimated to be around £6 billion per year in the UK, and projected to continue to rise in the coming years.[6] Therefore, prevention strategies targeting the primary school years are essential to address this significant health and economic burden.

Over the last decade, prevention strategies have shifted from focusing on individuals to a systems approach;[4,7-9] recognising the multiple interconnecting physical, economic, political, social and cultural influences. Within this context, the environments in which children are situated are critical to consider.

Schools provide a physical, social and educational environment for children and have the ability to shape physical activity (PA) and eating behaviours.[4] However, to date, our understanding of how the whole school environment influences these behaviours, and impacts on childhood obesity, is relatively limited. Research has mainly focused on evaluating specific intervention programmes introduced into schools to target obesity-related behaviours. A recent comprehensive Cochrane systematic review of childhood obesity prevention interventions reported that in the primary school-aged population there is some evidence to suggest that combined dietary and PA interventions in schools had a small positive effect on body mass index z-scores (zBMI; mean difference between intervention and control groups of −0.05, 95% CI −0.10 to −0.01; based on meta-analysis of 20

For peer review only

randomised controlled trials, but with low certainty of evidence).[10] However, there was no evidence to suggest that PA or dietary interventions on their own influenced zBMI.[10]

Although previous trials, using objective outcomes, suggest that school based interventions have the ability to increase PA levels;[11-14] a recent systematic review found no evidence of an effect of activity interventions on increasing whole-day moderate-to-vigorous physical activity (MVPA; standardised mean difference 0.02, 95% CI -0.07 to 0.11).[15]

Two systematic reviews have explored the effectiveness of school food environment interventions and policies on dietary behaviours. These have reported some beneficial effects, such as increased vegetable consumption, but have found no evidence of effect of these interventions on total energy intake.[12,16] However, there was significant heterogeneity of included studies in terms of the differences between schools and educational systems, which makes it challenging to draw general conclusions.

In general, whilst useful for considering specific intervention programmes designed to target diet and/or PA, the existing research evidence gives little information on how the combination of different policies, initiatives and activities in schools (the school environment) promotes PA, healthy eating (HE), and consequently impacts obesity in children. Among the few studies that have examined the relationship between school level characteristics and obesity-related behaviours, the findings have been mixed and concluded that further research is needed.[17-18]

Objectives

The study reported here aims to explore the association between school environments promoting HE and/or PA, and child anthropometric, dietary intake and PA in UK primary school children.

For peer review only - http://bmjopen.bmj.com/site/about/guidelines.xhtml

METHODS

Design

This longitudinal, observational study uses data obtained from the West Midlands ActiVe lifestyle and healthy Eating in School children (WAVES) study; a cluster randomised controlled trial which evaluated the clinical and cost-effectiveness of a 12-month obesity-prevention intervention programme, delivered in primary schools.[19] The trial was undertaken in 54 state-primary schools in the West Midlands between 2011 and 2015.

Participants

The WAVES sampling strategy sought to over-recruit schools with a high minority ethnic population and accounted for the proportion of children eligible for free school meals and, school size and location, ensuring a multi-ethnic and socio-economically diverse study sample. Full details regarding the recruitment and sampling methods are described in the WAVES study protocol.[20] All Year One pupils (age five-to-six) in participating schools were eligible to take part (n=2,462 children). Parental consent was obtained for 60% of children (n=1,470), of whom 1,392 were measured at baseline. Parent questionnaires and school provided information at baseline were used to provide demographic data for the children. Home postcodes were obtained for children and mapped to Index of Multiple Deprivation 2010 scores,[21] to give an indicator of socio-economic status.

Pupil outcome measures

Participating pupils took part in assessments undertaken by trained researchers following standardised operating procedures. Apart from demographic data, which was only obtained at baseline, all outcome measures were obtained at three time points: baseline (age five-to-six years), follow-up-one (age seven-to-eight years) and follow-up-two (age eight-to-nine years).

For peer review only

The outcomes of interest in this study were: zBMI; waist circumference z-score (zWC) (both z-scores calculated using UK 1990 reference curves for children;[22] total energy intake (kJ/24 hours); fruit and vegetable intake (g/24 hours and portions); PA energy expenditure (PAEE; kJ/kg body weight/24 hours); and estimated time spent in MVPA (min/24 hours; defined as time spent above the acceleration threshold of 1.75 m/second.[23] Measurement instruments and processes are summarised in Table 1, and more details are available in the WAVES trial report.[24]

For peer review only

Measurement	Instrument	Number of measurements	Method of assessment	Outcome variable
**Weight**	Tanita bioimpedance monitor (Tanita SC-331S; Tanita, Tokyo, Japan)	Once	Barefoot and in light clothing	**zBMI:** BMI was calculated by dividing weight (kilograms) by height (metres squared). UK 1990 growth reference charts were used to produce an age- and sex-specific zBMI and define weight categories.[38] Overweight is defined as ≥ 85th centile and obese as ≥ 95th centile.[38]
**Height**	Leicester height measure	Twice (third measure if difference >0.4cm)†	Barefoot and in light clothing	
**Demographic data**	Parent questionnaire	N/A	Parent report and school record	
**Waist Circumference (to nearest 0.1cm)**	Flexible, non-stretch, cloth tape measure	Twice (third measure if difference >0.4cm)†	Measure at iliac crest	**zWC:** calculated from WC using UK reference curves.[38]
**Dietary intake**	CADET: A validated 115 item 24-hour food tick list completed for 7 distinct time periods.[39]	Once (24 hours)	Completed by trained researchers in school, and parent/carer at home (with instructional DVD). Processed through CADET nutrient analysis program at the University of Leeds.	The food and nutrient intake data was used to calculate: **dietary total energy intake,** and **fruit and vegetable intake** based on the Englyst method.[40]
**Physical activity**	Actiheart (Cambridge Neurotechnology, Papworth, UK)	Once (worn continuously for five days, including one weekend)	Fitted in school by a trained researcher	Total movement volume was summarised as average acceleration, along with its underlying movement intensity distribution, using the acceleration and heart rate signals. This was used to calculate: **PA energy expenditure** ¶ and **estimated time spent in MVPA.**

Table 1: Summary of Measurements Undertaken and their Associated Outcome Variables

† Where two values within ≤ 0.4cm, a definitive measurement value was calculated as the average of the two. For individuals with three values recorded, a definitive measurement value was calculated as the average of the closest pair (within ≤ 0.4cm), or average of all three reading (if there were no two closest readings, but the differences between values were ≤ 0.4cm). When none of the three values were within 0.4cm of each other, no definitive measurement value was calculated.

¶ Children with < 24 hours of valid data were excluded. To ensure representation across the whole 24-hour period, for those with 24 hours of valid data, only those with a distribution of at least six hours in each quadrant (morning 3 am – 9 am; noon 9 am – 3 pm; afternoon 3 pm – 9 pm; and midnight 9 pm – 3 am) were included

WC, waist-circumference. CADET, Child and Diet Evaluation Tool. zBMI, body mass index z-score. BMI, body mass index. zWC, waist circumference z-score. PA, physical activity. MVPA, moderate-to-vigorous-physical -activity

For peer review only - http://bmjopen.bmj.com/site/about/guidelines.xhtml

Assessment of the school environment

Schools participating in the WAVES trial were asked to complete a baseline questionnaire, which enquired about the school's HE and PA environments. The questionnaire was completed by the head teacher or a nominated staff representative and included tick-box questions, followed by open-ended questions to gain further detail regarding policies and practices in the school. The Health Promoting School framework requirements, government guidelines and prior research were used to develop a scoring system to categorise schools regarding their health promotion environment based on their questionnaire responses. (Supplementary File Table 1).[16,25-27] The scoring system was developed in an iterative way, by the study team, until a consensus was reached on the scoring method and weighting. The use of government publications ensured that the scoring system encompassed key items that have been determined as important for national policy relating to child health at school.

Each question contributed to the final scores. Questions with continuous response variables (such as number of active after school clubs on offer), were categorised into three groups: high; medium; low, using tertile cut-offs with a score allocation of 2;1;0, respectively. Questions with categorical response variables were either ordinal or binary (yes/no) questions. Binary questions, such as schools having a healthy eating policy, were scored as 2/0. For the ordinal variables: schools allocating more than two hours to Physical Education (PE) was considered of high importance, they were grouped into schools providing this for all years; some years; no years with a score of 2;1;0; while level of support from stakeholders (strongly support; support; weakly support) was deemed as less important thus scored out of 1 (1;0.67;0.33 respectively.

We developed separate HE and PA scores based on a sum of scores from 15 and 20 questions (range of possible scores 44%-92% and 24%-92%), respectively. Using the range of scores across all schools, we used tertile cut-offs to split HE and PA scores into three groups. Schools were then categorised according to their health promotion environment: i) complete health focus, supporting

For peer review only - http://bmjopen.bmj.com/site/about/guidelines.xhtml

both HE and PA (top tertile for both HE and PA scores); ii) HE focus (top tertile for HE scores only); iii) PA focus (top tertile for PA scores only); iv) no health focus (all other schools).

Data analysis

All analyses were undertaken using STATA Version 15. Descriptive analyses were used to describe pupil characteristics at baseline and both follow-up time points. Categorical variables were summarised as numbers (percentage). Continuous data were inspected for normality and found to be skewed, so were summarised as medians (interquartile ranges (IQR)).

Due to the clustered nature of the data, multilevel linear regression models were constructed to explore the relationship between the school health promotion environment category and pupil outcome measures at the three time-points. School was included in all models as a random effect. Individual-level variables (baseline age, sex, ethnicity, Index of Multiple Deprivation score) were included in the models as fixed-effect covariates. The models for outcomes at the follow-up time-points also included the baseline outcome value and the trial-arm of the WAVES study as fixed-effect covariates. The models were further developed by including the school health promotion environment category as a fixed-effect covariate. This allowed estimation of the association between the school health promotion environment category and the outcome variable, adjusting for the clustering effect of schools and for individual factors. Pupils with missing data either at baseline or follow up were not included in the analyses.

RESULTS

Of the 54 schools participating in the WAVES trial, 50 (93%) returned the baseline questionnaire. It is these schools and the pupil participants attending them (n=1,304, 94% of the WAVES trial sample) that were included in the current analyses.

For peer review only - http://bmjopen.bmj.com/site/about/guidelines.xhtml

For peer review only

School characteristics

All schools had some activities or policies that promoted HE or PA. Overall schools gained higher scores for PA than HE promotion, with the mean normalised score (adjusted for number and type of questions) being 76% (IQR: 66–85%) for PA and 67% (IQR: 51–74%) for HE. Eight (16%) schools were classed as PA focus, 10 (20%) as HE focus, 10 (20%) as complete health focus and 22 (44%) as no health focus. The application of the scoring system to schools based on their questionnaire responses, including information on missing data, is shown in Table 2.

**Description of variable**	**Number of schools (%)**		
**HEALTHY EATING SCORE**			
	**No (score=0)**		**Yes (score =2)**
School has a policy regarding HE*	11 (22.45)		38 (77.55)
HE is promoted in school curriculum	10 (20.00)		40 (80.00)
School cookery club to promote HE	29 (58.00)		21 (42.00)
Involve parents in the promotion of HE	27 (54.00)		23 (46.00)
Drinking water is provided throughout the day*	0 (0.00)		49 (100.00)
Breakfast club is available for students	15 (30.00)		35 (70.00)
Food provided by the school at break deemed as healthy*	32 (65.31)		17 (34.69)
	**Low (score=0)**	**Medium (score =1)**	**High (score =2)**
Provide a range of ways to promote HE	12 (24.00)	13 (26.00)	25 (50.00)
Proportion of children choosing to eat school lunches†	12 (32.43)	11 (29.73)	14 (37.84)
	**Weakly support (score=0.33)**	**Support (score=0.67)**	**Strongly support (score=1)**
Governors support HE in school	2 (4.00)	31 (62.00)	17 (34.00)
Senior Leadership Team support HE in school*	0 (0.00)	21 (42.86)	28 (57.14)
Staff support HE in school*	0 (0.00)	27 (55.10)	22 (44.90)
School council support HE in school*	2 (4.08)	25 (51.02)	22 (44.90)
Pupils support HE in school*	5 (10.20)	35 (71.43)	9 (18.37)
Parents support HE in school*	14 (28.57)	31 (63.27)	4 (8.16)
**PHYSICAL ACTIVITY SCORE**			
	**No (score=0)**		**Yes (score=2)**

For peer review only - http://bmjopen.bmj.com/site/about/guidelines.xhtml

Description of variable	Number of schools (%)		
School has a policy regarding PA**	3 (6.25)	45 (93.75)	
School has a PA co-ordinator***	4 (8.51)	43 (91.49)	
Staff are trained regarding PA***	17 (36.17)	30 (63.83)	
Has sport partnerships and community links***	11 (23.40)	36 (76.60)	
PA is promoted in the school curriculum**	6 (12.50)	42 (87.50)	
Involve parents in the promotion of PA**	32 (66.67)	16 (33.33)	
School has a walk to school campaign**	13 (27.08)	35 (72.92)	
PE is taught by a specialist**	21 (43.75)	27 (56.25)	
Range of activities offered in PE meet Department of Education recommendations**	3 (6.25)	45 (93.75)	
	**Low (score=0)**	**Medium (score=1)**	**High (score=2)**
Provide a range of ways to promote PA**	7 (14.58)	20 (41.67)	21 (43.75)
Variety of PA opportunities other than PE**	10 (20.83)	15 (31.25)	23 (47.92)
Have a range of play equipment to be used**	12 (25.00)	18 (37.50)	18 (37.50)
Provide a range of sports clubs**	12 (25.00)	11 (22.92)	25 (52.08)
	**For no years (score=0)**	**For some years (score=1)**	**For all years (score=2)**
Schools allocating two or more hours to PE **	7 (14.58)	2 (4.17)	39 (81.25)
	**Weakly support (score=0.33)**	**Support (score=0.67)**	**Strongly support (score=1)**
Governors support PA in school**	0 (0.00)	28 (58.33)	20 (41.67)
Senior Leadership Team support PA in school**	0 (0.00)	18 (37.50)	30 (62.50)
Staff support PA in school**	0 (0.00)	22 (45.83)	26 (54.17)
School council support PA in school***	0 (0.00)	24 (51.06)	23 (48.94)
Pupils support PA in school**	0 (0.00)	22 (45.83)	26 (54.17)
Parents support PA in school**	4 (8.33)	33 (68.75)	11 (22.92)

Table 2: Summary of Results of the Scoring of Schools

* Missing data from 1 school**,** ** Missing data from 2 schools, *** Missing data from 3 schools, † Missing data from 13 schools

Missing data values are not included in the denominator used for percentage calculations.

HE, healthy eating. PA, physical activity. PE, Physical Education.

For peer review only

Not all schools reported having HE or PA policies (11 (22%) with no HE policy; three (6%) with no PA policy). However, several of these schools were placed in the PA focus, HE focus or complete health focus categories as they scored highly in other aspects of health promotion activity. All schools provided drinking water throughout the school day. Thirty-five (70%) had a breakfast club available to students. However, it was difficult to assess how healthy the food provided was from

For peer review only - http://bmjopen.bmj.com/site/about/guidelines.xhtml

the information available and only 17 schools (35%) provided a healthy break snack. Forty-five schools (94%) offered a range of activities during PE that met the Department of Education recommendations and 39 (81%) allocated two or more hours to PE. However, PE was taught by a specialist in only 27 schools (56%). Parents were perceived to support the HE and PA environment less than other stakeholders in the school. In addition, only 23 (46%) and 16 (33%) of schools involved parents in HE and PA promotion, respectively.

Pupil characteristics

Baseline characteristics for pupils are shown in Table 3. The sociodemographic characteristics of the WAVES trial participants excluded from these analyses (due to non-return of school questionnaires) were compared to the study sample and did not differ significantly by age or gender. However, the study sample were less likely to be of White British ethnicity, and more likely to be of South Asian ethnicity, or in the most deprived Index of Multiple Deprivation score quintile. Anthropometric, dietary and PA characteristics did not differ between the groups.

	**Baseline value for those analysed in this study (n=1304)**
**Median (IQR) age (years); missing data***	6.3 (6.0 – 6.5); 61
**Gender: n (%)**	
**Boys**	654 (50.15)
**Girls**	650 (49.85)
**Missing data***	0
**Ethnicity: n (%)**	
**White British**	656 (50.81)
**South Asian**	333 (25.79)
**Afro-Caribbean**	100 (7.75)
**Other**	202 (15.65)
**Missing data***	13
**Deprivation quintile: n (%)†**	
**1 (most deprived)**	639 (49.96)
**2**	266 (20.80)
**3**	146 (11.42)
**4**	119 (9.30)
**5 (least deprived)**	109 (8.52)
**Missing data***	25
**Median deprivation score (IQR)†; missing data***	34.0 (17.9 – 47.6); 25

Table 3: Summary of Pupil Demographic Information at Baseline

* Missing data values are not included in the denominator.

For peer review only - http://bmjopen.bmj.com/site/about/guidelines.xhtml

For peer review only

† Index of multiple deprivation scores categorised into five groups using the national quintile cut-offs for England. IQR, interquartile range.

Outcome measures are summarised for all three time-points in Table 4. The prevalence of overweight/obesity in the study population was 20.81% at baseline, 26.83% at follow-up-one (15 months) and 31.06% at follow-up-two (30 months). Prevalence varied across schools from 8 – 32% at baseline, 9 – 47% at follow-up-one and 9 – 53% at follow-up-two. There was a statistically significant increase between baseline and follow-up-two for zBMI and zWC ($p<0.001$). As would be expected, total energy intake increased over the study period as the participating children grew and increased their energy requirement. However, a statistically significant decrease in PAEE and estimated time spent in MVPA ($p<0.001$) was also observed during this period. There was no significant difference in fruit and vegetable intake over time ($p=0.20$).

For peer review only - http://bmjopen.bmj.com/site/about/guidelines.xhtml

	Baseline	Follow-up one	Follow-up two
**ANTHROPOMETRIC**			
**Median (IQR) zBMI; missing data***	0.095 (-0.550 - 0.839); 64	0.177 (-0.565 - 1.126); 201	0.270 (-0.580 - 1.353); 293
**BMI group category: n (%)†**			
**Underweight (≤2nd centile)**	29 (2.34)	23 (2.09)	23 (2.27)
**Healthy (>2nd and <85th centiles)**	953 (76.85)	784 (71.08)	674 (66.67)
**Overweight (≥85th and <95th centiles)**	109 (8.79)	126 (11.42)	117 (11.57)
**Obese (≥95th centile)**	149 (12.02)	170 (15.41)	197 (19.49)
**Missing data***	64	201	293
**Median (IQR) zWC; missing data***	0.679 (-0.027 – 1.437); 167	0.891 (0.180 – 1.842); 275	0.942 (0.144 – 2.025); 416
**DIETARY INTAKE**			
**Median (IQR) dietary total energy intake (kJ/ 24 hours); missing data***	6976.9 (5933.9 – 8101.8); 212	7150.3 (6100.0 – 8279.0); 301	7728.8 (6595.0 – 9128.9); 393
**Median (IQR) fruit and vegetable intake (g/ 24 hours); missing data***	241.8 (149.6 – 352.6); 212	207.8 (111.5 – 320.2); 301	219.5 (117.0 – 346.5); 393
**≥ 5 portion fruit and vegetables: n (%)**			
**Yes**	705 (64.56)	502 (50.05)	519 (56.97)
**No**	387 (35.44)	501 (49.95)	392 (43.03)
**Missing data***	212	301	393
**PHYSICAL ACTIVITY**			
**Median (IQR) PAEE (kJ/kg body weight/ 24 hours); missing data***	91.7 (78.0 – 108.0); 341	89.4 (73.9 – 106.0); 521	75.9 (63.3 – 93.1); 680
**Median (IQR) estimated time spent in MVPA (min/ 24 hours); missing data***	58.1 (41.9 – 81.7); 345	62.0 (39.8 – 101.9); 523	43.1 (31.6 – 64.2); 672

Table 4: Summary of Pupil Outcome Measures at Three Timepoints

* Missing data values are not included in the denominator.

† Based on UK 1990 reference centile curves and applying cut-offs used for population monitoring.

IQR, interquartile range. zBMI, body mass index z-score. BMI, body mass index. zWC, waist circumference z-score. PAEE, physical activity energy expenditure. MVPA, moderate-to-vigorous physical activity.

For peer review only - http://bmjopen.bmj.com/site/about/guidelines.xhtml

Associations between the school health promotion environment category and childhood anthropometric, diet and PA outcomes

For all models (i.e. for each outcome and at each time-point), the regression coefficients (B) for the school health promotion environment categories are presented in Table 5, with the no health focus category as the reference. Regression coefficients for all fixed-effect covariates in the models are presented in Supplementary File Tables 2-7. School health promotion environment category was not statistically significantly associated with child zBMI at baseline or follow-up-one. At follow-up-two, compared with no health focus schools, pupils in PA focus schools had a lower zBMI (B=-0.166, 95% CI -0.305 to -0.028, p=0.02). Although not statistically significant, compared to those in schools with no health focus, children in schools with a complete health focus also had a lower zBMI at follow-up-two (B=-0.106, 95% CI -0.245 to 0.034, p=0.14). At follow-up-two, PA focus and complete health focus schools also had a lower zWC than no health focus schools, but this was non-significant (PA focus: B=-0.151, 95% CI -0.361 to 0.058, p=0.16 and complete health focus: B=-0.175, 95% CI -0.377 to 0.027, p=0.09).

Children in schools in HE, PA and complete health focus categories had significantly lower daily PAEE at baseline than those in no health focus schools (PA focus: B=-6.0, 95% CI -11.7 to -0.3, p=0.04; HE focus: B=- 6.3, 95% CI -11.8 to – 0.9, p=0.02; and complete health focus: B=-6.3, 95% CI -11.6 to -1.0, p=0.02). They also did less MVPA at baseline, however, this was only significant for schools with a complete health focus (B=-14.1, 95% CI -27.4 to -0.8, p=0.04). By follow-up-two, this trend reversed, with higher levels of both PAEE and MVPA in the HE, PA and complete health focus schools, compared with no health focus schools, although these findings were not significant.

School health promotion environment category was not significantly associated with total dietary energy intake or fruit and vegetable intake.

For peer review only

Outcome variable	School health promotion environment category *	Baseline		Follow-up-one		Follow-up-two	
		Regression coefficient (95% CI)	p-value	Regression coefficient (95% CI)	p-value	Regression coefficient (95% CI)	p-value
zBMI	PA focus	-0.055 (-0.247 to 0.137)	0.57	-0.058 (-0.220 to 0.104)	0.48	**-0.166 (-0.305 to -0.028)**	**0.02**
	HE focus	-0.092 (-0.277 to 0.093)	0.33	-0.015 (-0.179 to 0.149)	0.86	-0.019 (-0.164 to 0.125)	0.80
	Complete health focus	0.044 (-0.143 to 0.231)	0.64	0.067 (-0.092 to 0.226)	0.41	-0.106 (-0.245 to 0.034)	0.14
zWC	PA focus	-0.102 (-0.360 to 0.156)	0.44	0.041 (-0.216 to 0.299)	0.75	-0.151 (-0.361 to 0.058)	0.16
	HE focus	-0.236 (-0.480 to 0.007)	0.06	0.192 (-0.065 to 0.450)	0.14	0.067 (-0.154 to 0.289)	0.55
	Complete health focus	0.104 (-0.137 to 0.344)	0.40	-0.009 (-0.255 to 0.238)	0.95	-0.175 (-0.377 to 0.027)	0.09
Dietary total energy intake (kJ/24 hours)	PA focus	-903.5 (-2402.7 to 595.6)	0.24	433.7 (-70.5 to 937.9)	0.09	33.2 (-614.5 to 681.0)	0.92
	HE focus	-847.8 (-2255.8 to 560.3)	0.24	-63.7 (-598.0 to 470.5)	0.82	260.8 (-443.1 to 964.7)	0.47
	Complete health focus	-399.3 (-1801.3 to 1002.6)	0.58	32.8 (-464.7 to 530.3)	0.90	171.3 (-466.4 to 809.1)	0.60
Fruit and vegetable intake (g/24 hours)	PA focus	-57.8 (-135.5 to 19.8)	0.14	11.8 (-27.9 to 51.5)	0.56	-16.0 (-73.9 to 41.9)	0.59
	HE focus	-31.6 (-104.7 to 41.4)	0.40	3.0 (-38.6 to 44.7)	0.89	-4.4 (-66.9 to 58.0)	0.89
	Complete health focus	-7.3 (-80.0 to 65.4)	0.84	27.1 (-11.9 to 66.0)	0.17	8.6 (-48.1 to 65.3)	0.77
PAEE (kJ/kg body weight/24 hours)	PA focus	**-6.0 (-11.7 to -0.3)**	**0.04**	-0.8 (-7.2 to 5.7)	0.81	1.9 (-3.8 to 7.6)	0.51
	HE focus	**-6.3 (-11.8 to -0.9)**	**0.02**	3.8 (-3.1 to 10.7)	0.28	3.5 (-2.7 to 9.7)	0.27
	Complete health focus	**-6.3 (-11.6 to -1.0)**	**0.02**	-2.6 (-8.8 to 3.6)	0.42	5.3 (0.0 to 10.6)	0.05
Estimated time spent in MVPA (min/24 hours)	PA focus	-4.2 (-18.5 to 10.1)	0.57	-3.7 (-22.7 to 15.2)	0.70	3.8 (-10.5 to 18.2)	0.60
	HE focus	-13.2 (-26.8 to 0.5)	0.06	-0.8 (-20.9 to 19.3)	0.94	0.8 (-14.5 to 16.1)	0.92
	Complete health focus	**-14.1 (-27.4 to -0.8)**	**0.04**	-6.4 (-24.7 to 11.8)	0.49	7.2 (-6.4 to 20.9)	0.30

Table 5: The Association between the School Health Promotion Environment Category and Pupil Obesity, Dietary and Physical Activity Outcome Measures, Estimated Using Multilevel Modelling

* No health focus schools are the reference category. All models include school as a random effect and age at baseline, sex, ethnicity, and index of multiple deprivation score as fixed-effect covariates. Models with outcomes at follow-up-one and follow-up-two also include the outcome variable at baseline and the WAVES trial arm as fixed-effect covariates.

Results shown in bold are statistically significant.

zBMI, body mass index z-score. zWC, waist circumference z-score. PAEE, physical activity energy expenditure. MVPA, moderate-to-vigorous physical activity. PA, physical activity. HE, healthy eating.

For peer review only - http://bmjopen.bmj.com/site/about/guidelines.xhtml

DISCUSSION

The questionnaire responses indicated that all schools promoted healthy behaviours to some degree, but there was variation in the extent of their efforts. The school scores indicated that schools tended to focus more on promoting PA than HE. This may relate to increased government focus and spending on physical activity in schools during the study period. Overall, we found little relationship between the school health promotion environment and pupil anthropometric measures, diet or PA levels. Most associations between the school health promotion environment category and the outcome measures were non-significant, and those that were significant were small in magnitude. However, by the second follow-up time point (30 months after baseline) there was a statistically significant lower mean zBMI among pupils in schools with a PA focus, compared with those in schools with no health focus. Although the effect size is small (mean difference= -0.166; 95% CI -0.305 to -0.028; p= 0.02), if successfully sustained it has the potential to produce public health benefits at a population level.[28] A similar finding was observed for zWC, although this was non-significant. In line with these associations between school health promotion activity and obesity-related outcomes, we found that at the second follow-up time point PAEE levels were higher in schools with a complete health focus compared with those with no health focus.

Strengths

To our knowledge, this is the first UK study to assess the association between primary school health promoting environments and child obesity measures longitudinally. The study involves a large sample, from an ethnically and socio-economically diverse population, over a three-year period. Outcomes were predominantly assessed by objective measures, using validated instruments and standardised procedures. Clustering and individual level confounders were accounted for by using multilevel models, which enables more confidence in the observed associations between the school health promoting environment and the outcomes.

For peer review only - http://bmjopen.bmj.com/site/about/guidelines.xhtml

Limitations

The sample includes only 50 primary schools that consented to be part of an obesity prevention trial and parental consent was only obtained for 60% of eligible children within these schools. Our study sample may therefore represent schools with more interest in health promotion and pupils who were healthier. However, obesity prevalence in the participating schools was compared with the prevalence estimated from the National Child Measurement Programme at the time and was found to be similar.[29] Comparisons of demographic characteristics of children with and without parental consent to participate in the WAVES trial (undertaken as part of the WAVES trial) also did not show major differences,[24] suggesting that the study sample is broadly representative. The questionnaire used to assess the school environment was developed for a different purpose by a multidisciplinary team for the WAVES trial, and was assessed for face validity, but not further validated. Schools completed the questionnaire with a varying degree of completeness; therefore, the resulting information may not fully reflect their health promoting activity. The scoring system was developed by the authors, based on past and present guidelines relating to health promoting schools; however, it has not undergone any validation. Furthermore, the categorisation process of schools was pragmatically determined based on statistical cut-offs, and there may be large variations in health promoting activities within each of the categories. It is possible that, due to limitations of the baseline questionnaire and the lack of validation of the scoring system, the schools' health promoting environments may not have been accurately represented, which in turn may have led to the equivocal findings of the study. Further limitations relate to the pupil outcome measures. The Child And Diet Evaluation Tool (CADET) was quick and practical with a relatively low response burden,[30] but it only measured dietary intake over a single day, so may not be an accurate assessment of habitual intake. There were significant missing data for PA measures, however this was comparable to other studies which objectively measured PA.[18]

For peer review only

For peer review only - http://bmjopen.bmj.com/site/about/guidelines.xhtml

Comparison with previous research

This study brings new information regarding the potential impact the school HE and PA environment has on children, as previous research has focused on the effectiveness of specific interventions and policies within schools.

Our findings are in line with those from two systematic reviews that found moderate evidence that school PA interventions are able to reduce BMI.[10,12] Our study included a range of school level activities, rather than focusing on specific interventions, and the effect size observed at 30 months was similar to the summary estimate from the trials.[10]

There is a large pool of research indicating PA interventions increase PA levels in children during the school day,[11-14] although overall PA levels may not be increased.[15] We found that children in schools with an increased focus on PA were less active and engaged in less MVPA at baseline compared with no health focus schools, but over time the trend was reversed, suggesting that school PA promotion may increase child PA levels over time. Our findings may also indicate that PA promotion in schools only affects PA levels as children get older, when their PA levels generally decrease.

Previous systematic reviews have not found promising results regarding the impact of interventions on dietary outcomes, although there is some evidence that they are able to increase fruit and vegetable intake.[12,16] This study did not find a relationship between the school health promotion environment and child dietary outcomes. The differences between the results of this study and previous research may be as a result of assessing overall environment, rather than specific targeted interventions. In 2018, the House of Commons (UK Parliament) stated that there was no convincing evidence the school healthy food standards had been implemented effectively.[31] The majority (n=32, 65%) schools in this study provided a break-time snack which was deemed unhealthy,

For peer review only - http://bmjopen.bmj.com/site/about/guidelines.xhtml

For peer review only

suggesting other school food provision may also be unhealthy. Furthermore, the limitations of the questionnaire in assessing school healthy eating environment and those related to the dietary assessment method may also have contributed to differences in findings.

In terms of health promoting environments, we found that school perception of parental support for HE in schools was not high (low parental support perceived by over a quarter of schools). Children consistently refer to home life being more significant in changing behaviour than schools and families have been shown to be pivotal in ensuring improvements to lifestyle in primary school aged children.[32-34] Our findings suggest that in relation to PA and HE, parent-school relationships need strengthening. Nevertheless, the complexity of promoting a healthy school environment and of including parents in such endeavours also need to be considered.[35]

Implications

Consistent with previous research, this study indicates that supporting PA in primary schools may have beneficial health effects in terms of increasing physical activity levels and reducing weight status. Although schools often prioritise academic attainment over PE and PA, increasing PE in the curriculum has not been found to have negative academic consequences and may improve school achievement.[36-37] Therefore, schools are recommended to increase their efforts to promote PA. In this study, school environments were not as supportive of healthy eating as expected, which is in line with UK Parliament's report stating that there was a lack of effective implementation of school food standards legislation.[31] Stronger enforcement of existing school food regulation and further regulatory measures may be needed to ensure healthy eating is fully supported in schools.

All associations between the school health promotion environment and health/behavioural outcomes were of small magnitude, which suggests it has an important but limited role in child health. Parents are key influencers of children's health,[33-34] but in this study the majority of schools did not

For peer review only - http://bmjopen.bmj.com/site/about/guidelines.xhtml

involve parents in their health promotion efforts. The development of school health promoting environments may be more effective if schools develop better relationships with parents and involve them in their health promotion efforts.

Conclusion

We found that over time, a school environment supportive of PA may positively influence zBMI. In addition, there is some indication that schools with a more health promoting environment may contribute to increased PA levels in children over time. More research is needed to further understand the influence of the school environment, but in the meantime, schools should be encouraged to provide an environment that is supportive of PA. However, schools cannot undertake health promotion in isolation. They need to be considered as part of a complex system, which requires intervention at multiple points to produce a sustained reduction in child overweight and obesity.

For peer review only

For peer review only - http://bmjopen.bmj.com/site/about/guidelines.xhtml

ACKNOWLEDGEMENTS

We would like to acknowledge the contribution of all the WAVES study trial investigators: University of Birmingham: Peymane Adab (Professor of Chronic Disease Epidemiology & Public Health and Chief Investigator), Tim Barrett (Leonard Parsons Professor of Paediatrics and Child Health), KK Cheng (Professor of Public Health and Primary Care), Jonathan J Deeks (Professor of Biostatistics), Joan L Duda (Professor of Sport and Exercise Psychology), Emma Frew (Professor in Health Economics), Karla Hemming (Professor of Biostatistics), Miranda J Pallan (Reader in Public Health & Epidemiology), Jayne Parry (Professor of Policy and Public Health); University of Cambridge, Cambridge MRC Epidemiology Unit / Norwegian School of Sport Sciences: Ulf Ekelund (Professor in Physical Activity and Health); University of Leeds: Janet E Cade (Professor of Nutritional Epidemiology and Public Health); Loughborough University: Amanda Daley (Professor of Behavioural Medicine); University of Edinburgh: Raj Bhopal (Emeritus Professor of Public Health); University of Warwick: Paramjit Gill (Professor of General Practice); Birmingham Community Healthcare NHS Trust: Eleanor McGee (Public Health Nutrition Lead); Birmingham Services for Education: Sandra Passmore (Education Advisor)

We thank the children, school staff, and parents who participated in the trial; the study team, including Behnoush Mohammadpoor Ahranjani and Emma Popo who helped in overseeing the study measurements and data collection; the administrative team who facilitated the running of the project; the research staff who undertook the study measurements; and Robert Lancashire who developed the trial database and oversaw data management. We also thank our collaborators at the Institute of Metabolic Science at the Medical Research Council (MRC) Epidemiology Unit (University of Cambridge) for their support and assistance, particularly Soren Brage, Kate Westgate, and Stefanie Hollidge, for training of field staff on use and maintenance of Actihearts, for overseeing data preparation, and for analysis and interpretation of the physical activity data; Timo Lakka (principal investigator of the PANIC study, University of Eastern Finland) for sharing

For peer review only

exercise data in a similarly aged cohort of children to allow translation of observed heart rate into activity energy expenditure; staff who are working, or have previously worked, at the Nutrition Epidemiology Group in Leeds, who have supported the trial team in terms of administration of the CADET food tick list and processing of the data to enable us to undertake dietary intake analysis, in particular Neil Hancock, Cristina Cleghorn, Meagan Christian, Jayne Hutchinson, Holly Rippin, and Catherine Rycroft. We also thank Sayeed Haque for his statistical advice on the analyses for this particular study.

HUMAN SUBJECTS APPROVAL STATEMENT

The WAVES trial was approved by NHS Research Ethics Service Committee West Midlands, The Black Country (NHS REC Number: 10/H1202/69) and sponsored by the University of Birmingham (Sponsor Number: RG_10-183). Parents gave written informed consent for children to participate and children gave verbal assent before measurements were taken. Permission to access the WAVES study data for this study was obtained in October 2018.

TRIAL MONITORING AND DATA MONITORING

The trial steering committee met annually and included: chair/statistician: Kelvin Jordan, Keele University; subject expert: Peter Whincup, St George's, University of London; health economist: Louise Longworth, Brunel University; public representative: John Bennett, PHSE advisor; and Chief Investigator: Peymane Adab, University of Birmingham.

FUNDING

This WAVES study/project was funded by the National Institute for Health Research (NIHR) Health Technology Assessment Programme (project reference 06/85/11). The views expressed are those of the authors and not necessarily those of the NIHR or the Department of Health and Social Care.

For peer review only - http://bmjopen.bmj.com/site/about/guidelines.xhtml

COMPETING INTERESTS

Miranda Pallan, Tania Griffin, Kiya Hurley, Emma Lancashire and Peymane Adab hold grants from the UK National Institute for Health Research, and Peymane Adab is Chair of the National Institute for Health Research Public Health Research Funding Committee. There are no other competing interests to declare.

AUTHOR CONTRIBUTION

Mairenn Garden (first author) designed and undertook the analysis reported in this manuscript, and drafted the manuscript. Miranda Pallan and Peymane Adab designed and oversaw the conduct of the trial from which the data is drawn, advised on the design and analysis of the reported study, and also contributed substantially to the manuscript. Joanne Clarke, Tania Griffin, Kiya Hurley and Emma Lancashire all designed school and pupil data collection tools and undertook data collection. They also substantially contributed to the manuscript. Sandra Passmore oversaw the main trial from which the data is drawn, advised on the development of the school data collection tool, and substantially contributed to the manuscript, bringing a school policy perspective. All authors approved the final version of the manuscript.

DATA SHARING STATEMENT

Requests for access to data from the WAVES trial should be addressed to the Principal Investigator at p.adab@bham.ac.uk. All proposals requesting data access will need to specify how it is planned to use the data, and all proposals will need approval of the trial co-investigator team before data release.

REFERENCES

For peer review only

1. Mead E, Brown T, Rees K, et al. Diet, physical activity and behavioural interventions for the treatment of overweight or obese children from the age of 6 to 11 years. *Cochrane Database Syst Rev* 2017; Issue 6. Art No.:CD012651 doi:10.1002/14651858.CD012651.
2. Sahoo K, Sahoo B, Choudhury AK, et al. Childhood obesity: causes and consequences. *J Family Med Prim Care* 2015;4(2):187–92 doi:10.4103/2249-4863.154628.
3. Stats Team. National Child Measurement Programme England, 2017/ 2018 School Year. 2018. Available from: https://files.digital.nhs.uk/9F/22AF4D/nati-chil-meas-prog-eng-2017-2018-rep.pdf. Accessed: 22 Jan 2019.
4. Butland B, Jebb S, Kopelman P, et al. Tackling Obesities: Future Choices-Project Report 2nd Edition. Government Office for Science. 2007. Available from: https://assets.publishing.service.gov.uk/government/uploads/system/uploads/attachment_data/file/287937/07-1184x-tackling-obesities-future-choices-report.pdf. Accessed: 25 Oct 2018.
5. Kipping RR, Payne C, Lawlor DA. Randomised controlled trial adapting US school obesity prevention to England. *Arch Dis Child* 2008;93(6):469-73 doi:10.1136/adc.2007.116970.
6. Public Health England. Health matters: obesity and the food environment. 2017. Available from: https://www.gov.uk/government/publications/health-matters-obesity-and-the-food-environment/health-matters-obesity-and-the-food-environment--2. Accessed: 12 Nov 2018.
7. Huang TT, Drewnosksi A, Kumanyika S, et al. A systems-oriented multilevel framework for addressing obesity in the 21st century. *Prev Chronic Dis* 2009;6(3):A82.
8. Rutter H. The single most important intervention to tackle obesity…. *Int J Public Health* 2012;57(4):657-8 doi:10.1007/s00038-012-0385-6.
9. Johnston L, Matteson C, Finegood D. Systems Science and Obesity Policy: A Novel Framework for Analyzing and Rethinking Population-Level Planning. *Am J Public Health* 2014;104(7):1270-8 doi:10.2105/AJPH.2014.301884.

For peer review only - http://bmjopen.bmj.com/site/about/guidelines.xhtml

10. Brown T, Moore THM, Hooper L, et al. Interventions for preventing obesity in children. *Cochrane Database Syst Rev*. 2019, Issue 7. Art No.:CD001871 doi:10.1002/14651858.CD001871.pub4.

11. Dobbins M, DeCorby K, Robeson P, et al. School-based physical activity programs for promoting physical activity and fitness in children and adolescents aged 6 – 18. *Cochrane Database Syst Rev* 2013, Issue 2. Art No.: CD007651 doi:10.1002/14651858.CD007651.pub2.

12. Langford R, Bonell C, Jones H, et al. The WHO Health Promoting School framework for improving the health and well-being of students and their academic achievement. *Cochrane Database Syst Rev* 2014, Issue 4. Art No.: CD008958 doi:10.1002/14651858.CD008958.pub2.

13. Drummy C, Murtagh EM, McKee DP, et al. The effect of a classroom activity break on physical activity levels and adiposity in primary school children. *J Paediatr Child Health* 2016;52(7):745–9 doi:10.1111/jpc.13182.

14. Wilkie HJ, Standage M, Gillison FB, et al. Correlates of intensity-specific physical activity in children aged 9-11 years: a multilevel analysis of UK data from the International Study of Childhood Obesity, Lifestyle and the Environment. *BMJ Open* 2018;8(2):e018373–e018373 doi:10.1136/bmjopen-2017-018373.

15. Love R, Adams J, can Sluijs EM. Are school-based physical activity interventions effective and equitable? A meta-analysis of cluster randomised controlled trials with accelerometer-assessed activity. *Obes Rev* 2019;20(6):859-870 doi:10.1111/obr.12823.

16. Micha R, Karageorgou D, Bakogianni I, et al. Effectiveness of school food environment policies on children's dietary behaviors: A systematic review and meta-analysis. *PLoS One* 2018;13(3):e0194555–e0194555 doi:10.1371/journal.pone.0194555.

17. Harrison F, Bentham G, Jones AP, et al. School level correlates with adiposity in 9-10 year old children. *Health Place* 2011;17(3):710-6 doi:10.1016/j.healthplace.2011.01.009.

For peer review only - http://bmjopen.bmj.com/site/about/guidelines.xhtml

18. Pallan MJ, Adab P, Sitch AJ, et al. Are school physical activity characteristics associated with weight status in primary school children? A multilevel cross-sectional analysis of routine surveillance data. *Arch Dis Child* 2014;99(2):135-41 doi:10.1136/archdischild-2013-303987.

19. Adab P, Pallan MJ, Lancashire ER, et al. Effectiveness of a childhood obesity prevention programme delivered through schools, targeting 6 and 7 year olds: cluster randomised controlled trial (WAVES study). *BMJ* 2018;360:k211 doi:10.1136/bmj.k211

20. Adab P, Pallan MJ, Lancashire ER, et al. A cluster-randomised controlled trial to assess the effectiveness and cost-effectiveness of a childhood obesity prevention programme delivered through schools, targeting 6–7 year old children: the WAVES study protocol. *BMC Public Health* 2015;15:488 doi:10.1186/s12889-015-1800-8

21. Department for Communities and Local Government (2011) The English Indices of Deprivation 2010. Available from: https://assets.publishing.service.gov.uk/government/uploads/system/uploads/attachment_data/file/6871/1871208.pdf. Accessed: 22 Jan 2019.

22. Cole TJ, Freeman J V, Preece MA. Body mass index reference curves for the UK, 1990. *Arch Dis Child* 1995;73(1):25–9 doi:10.1136/adc.73.1.25

23. World Health Organization. Physical status: the use and interpretation of anthropometry [WHO technical report series 854]. Geneva 1995. Available from: https://www.who.int/childgrowth/publications/physical_status/en/. Accessed: 8 Feb 2019.

24. Adab P, Barrett T, Bhopal R, et al. The West Midlands ActiVe lifestyle and healthy Eating in School children (WAVES) study: a cluster randomised controlled trial testing the clinical effectiveness and cost-effectiveness of a multifaceted obesity prevention intervention programme targeted at children aged 6–7 years. Southampton (UK): NIHR Journals Library; 2018 Feb. (Health Technology Assessment, No. 22.8.) doi:10.3310/hta22080

For peer review only - http://bmjopen.bmj.com/site/about/guidelines.xhtml

25. The Requirements of School Food Standards 2014 (SI 2014/1603). Available from: http://www.legislation.gov.uk/uksi/2014/1603/contents/made. Accessed: 22 Jan 2019.

26. Department of Health and Department for Children, Schools and Families. Introduction to the National Healthy Schools Programme. Available from: http://dera.ioe.ac.uk/6798/7/Introduction_Redacted.pdf. Accessed: 27 Oct 2018.

27. Evans C EL, Mandl V, Christian MS, et al. Impact of school lunch type on nutritional quality of English children's diets. *Public Health Nutr* 2016;19(1):36–45 doi:10.1017/S1368980015000853.

28. Rose G. Sick individuals and sick populations. Int J Epidemiol. 1985;14(1):32–8.

29. Conolly A. Health Survey for England 2015 Children's body mass index, overweight and obesity. NHS Digital [ISBN: 978-1-78386-896-4]. 2016. Available from: http://healthsurvey.hscic.gov.uk/media/37776/hse2015-child-obe.pdf. Accessed: 22 Jan 2019.

30. Zaborskis A, Moceviciene R, Iannotti RJ. The Influence of Chronological Period of Data Collection on Differences in Reported Dietary Intake Among School-Aged Children Surveyed in 39 Countries. *J Nutr Educ Behav* 2014;46(5):359–69 doi:10.1016/j.jneb.2013.11.017.

31. Health Committee. Childhood obesity: Time for action (HC 882). London: The Stationery Office; 2018.

32. Fairbrother H, Curtis P, Goyder E. Where are the schools? Children, families and food practices. *Health Place* 2016;40:51-7 doi:10.1016/j.healthplace.2016.04.013.

33. Golan M, Weizman A. Familial Approach To The Treatment Of Childhood Obesity: Conceptual Model. *J Nutr Educ* 2001;33(2):102–7 doi:10.1016/s1499-4046(06)60173-5.

34. Esposito L, Fisher JO, Mennella JA, et al. Developmental perspectives on nutrition and obesity from gestation to adolescence. *Prev Chronic Dis* 2009;6(3):A94.

For peer review only - http://bmjopen.bmj.com/site/about/guidelines.xhtml

35. S Passmore, L Jones. A review of the sustainability and impact of a healthy lifestyles programme in primary schools 2–5 years after the intervention phase. *Health Educ Res* 2019;34(1):72-83 doi:10.1093/her/cyy043.

36. Story M, Nanney MS, Schwartz MB. Schools and obesity prevention: creating school environments and policies to promote healthy eating and physical activity. *Milbank Q* 2009; 87(1):71–100 doi:10.1111/j.1468-0009.2009.00548.x.

37. Martin A, Booth JN, Laird Y, et al. Physical activity, diet and other behavioural intervention for improving cognition and school achievement in children and adolescents with obesity or overweight. *Cochrane Database Syst Rev* 2018, Issue 3. Art. No.: CD009728 doi:10.1002/14651858.CD009728.pub4.

Table references

38. Cole TJ, Freeman J V, Preece MA. Body mass index reference curves for the UK, 1990. Arch Dis Child 1995;73(1):25–9 doi:10.1136/adc.73.1.25

39. Cade JE, Frear L, Greenwood DC. Assessment of diet in young children with an emphasis on fruit and vegetable intake: using CADET – Child and Diet Evaluation Tool. *Public Health Nutr* 2006; 9(4):501–8 doi: 10.1079/phn2005871

40. Englyst HN, Cummings JH. Improved method for measurement of dietary fiber as non-starch polysaccharides in plant foods. *J Assoc Off Anal Chem* 1988; 71(4):808—814.

For peer review only

Relationship between primary school healthy eating and physical activity promoting environments and children's dietary intake, physical activity and weight status: a longitudinal study in the West Midlands, United Kingdom

Supplementary file

Table 1: School Environment and Policy Scoring

Description of variable	Score allocation
**HEALTHY EATING SCORE**	
School has a policy regarding HE	0 = No<br>2 = Yes
HE is promoted in school curriculum	0 = No<br>2 = Yes
School cookery club to promote HE	0 = No<br>2 = Yes
Involve parents in the promotion of HE	0 = No<br>2 = Yes
Provide a range of ways to promote HE	Split data into tertiles<br>0 = < 5<br>1 = $5 \leq x < 7$<br>2 = ≥ 7
Drinking water is provided throughout the day	0 = No<br>2 = Yes
Breakfast club is available for students	0 = No<br>2 = Yes
Food available at break deemed as healthy	0 = No†<br>2 = Yes†
Proportion of children choosing to eat school lunches	Split data into tertiles<br>0 = < 50%<br>1 = 50% ≤ x < 60%<br>2 = ≥ 60% of children
Governors support HE in school *	0.33 = Weakly support<br>0.67 = Support<br>1.00 = Strongly support
The Senior Leadership Team support HE in school *	0.33 = Weakly support<br>0.67 = Support<br>1.00 = Strongly support
Staff support HE in school *	0.33 = Weakly support<br>0.67 = Support<br>1.00 = Strongly support
School council support HE in school *	0.33 = Weakly support<br>0.67 = Support<br>1.00 = Strongly support
Pupils support HE in school *	0.33 = Weakly support<br>0.67 = Support<br>1.00 = Strongly support
Parents support HE in school *	0.33 = Weakly support<br>0.67 = Support<br>1.00 = Strongly support
**PHYSICAL ACTIVIY SCORE**	
School has a policy regarding PA	0 = No<br>2 = Yes
School has a PA coordinator	0 = No<br>2 = Yes
Staff are trained regarding PA	0 = No<br>2 = Yes

For peer review only

School has sport partnerships and community links	0 = No<br>2 = Yes
PA is promoted in the school curriculum	0 = No<br>2 = Yes
Involve parents in the promotion of PA	0 = No<br>2 = Yes
Have a walk to school campaign	0 = No<br>2 = Yes
Provide a range of ways to promote PA	Split data into tertiles<br>0 = < 5<br>1 = 5 ≤ x < 7<br>2 = ≥ 7
PE is taught by a specialist	0 = no<br>2 = yes
Range of activities offered in PE meet Department of Education recommendations	0 = no<br>2 = yes
Schools allocating two or more hours to PE	0 = for none of the school<br>1 = for some of the years<br>2 = for the whole school
Offer a variety of PA opportunities, other than PE	Split data into tertiles<br>0 = 0<br>1 = 1<br>2 = ≥ 2
Have a range of play equipment to be used	Split data into tertiles<br>0 = < 5<br>1 = 5<br>2 = ≥ 6
Provide a range of physically active/ sports clubs	Split data into tertiles<br>0 = < 4<br>1 = 4 ≤ x < 6<br>2 = ≥ 6
Governors support PA in school *	0.33 = Weakly support<br>0.67 = Support<br>1.00 = Strongly support
The Senior Leadership Team support PA in school *	0.33 = Weakly support<br>0.67 = Support<br>1.00 = Strongly support
Staff support PA in school *	0.33 = Weakly support<br>0.67 = Support<br>1.00 = Strongly support
School council support PA in school *	0.33 = Weakly support<br>0.67 = Support<br>1.00 = Strongly support
Pupils support PA in school *	0.33 = Weakly support<br>0.67 = Support<br>1.00 = Strongly support
Parents support PA in school *	0.33 = Weakly support<br>0.67 = Support<br>1.00 = Strongly support

* Given a maximum score of 1, to ensure that level of support by respective groups did not account for too great a proportion of overall score.
† No = Any food other than fruit, vegetables, milk and juice. Yes = Fruit, vegetables, milk and juice

For peer review only

Table 2: Multilevel models developed for Body Mass Index z-score at the three study time points: all fixed effect covariates are presented

	Baseline		Follow-up one		Follow-up two	
	**B (95% CI)**	**p-value**	**B (95% CI)**	**p-value**	**B (95% CI)**	**p-value**
**Trial arm**	N/A	N/A	0.072 (-0.049 to 0.193)	0.24	0.032 (-0.073 to 0.137)	0.55
**Baseline zBMI**	N/A	N/A	0.952 (0.925 to 0.978)	< 0.01	0.921 (0.885 to 0.956)	< 0.01
**Index of Multiple Deprivation score**	0.004 (-0.001 to 0.008)	0.09	0.000 (-0.003 to 0.002)	0.74	0.000 (-0.003 to 0.003)	0.80
**Female**	-0.082 (-0.216 to 0.052)	0.23	-0.110 (-0.175 to -0.045)	<0.01	-0.159 (-0.244 to -0.074)	< 0.01
**Ethnicity**						
**Asian**	-0.266 (-0.437 to -0.095)	<0.01	0.103 (0.009 to0.197)	0.03	0.161 (0.048 to 0.275)	0.01
**African Caribbean**	0.375 (0.105 to 0.645	0.01	0.076 (-0.064 to 0.216)	0.29	0.005 (-0.182 to 0.193)	0.96
**Other**	-0.022 (-0.225 to 0.181)	0.83	0.014 (-0.089 to 0.118)	0.78	0.035 (-0.099 to 0.169)	0.61
**Age**	0.034 (-0.186 to 0.254)	0.76	-0.019 (-0.126 to 0.088)	0.73	0.018 (-0.123 to 0.160)	0.80
**School health promotion environment category**						
**PA focus**	-0.055 (-0.247 to 0.137)	0.57	-0.058 (-0.220 to 0.104)	0.48	-0.166 (-0.305 to -0.028)	0.02
**HE focus**	-0.092 (-0.277 to 0.093)	0.33	-0.015 (-0.179 to 0.149)	0.86	-0.019 (-0.164 to 0.125)	0.80
**Complete health focus**	0.044 (-0.143 to 0.231)	0.64	0.067 (-0.092 to 0.226)	0.41	-0.106 (-0.245 to 0.034)	0.14

For peer review only

Table 3: Multilevel models developed for Waist Circumference Z-score at the three study time points: all fixed effect covariates are presented

	Baseline		Follow-up one		Follow-up two	
	B (95% CI)	p-value	B (95% CI)	p-value	B (95% CI)	p-value
**Trial arm**	N/A	N/A	-0.116 (-0.305 to 0.074)	0.23	-0.090 (-0.249 to 0.069)	0.27
**Baseline zWC**	N/A	N/A	0.898 (0.861 to 0.935)	< 0.01	0.845 (0.801 to 0.889)	< 0.01
**Index of Multiple Deprivation score**	0.002 (-0.003 to 0.007)	0.35	-0.001 (-0.004 to 0.003)	0.76	0.001 (-0.003 to 0.005)	0.48
**Female**	0.072 (-0.069 to 0.213)	0.32	-0.062 (-0.151 to 0.027)	0.17	0.110 (0.006 to 0.214)	0.04
**Ethnicity**						
**Asian**	-0.198 (-0.388 to -0.009)	0.04	0.193 (0.064 to 0.322)	<0.01	0.212 (0.068 to 0.356)	<0.01
**African Caribbean**	0.329 (0.042 to 0.617)	0.03	0.015 (-0.176 to 0.205)	0.88	0.070 (-0.169 to 0.310)	0.57
**Other**	0.004 (-0.211 to 0.219)	0.97	0.059 (-0.082 to 0.200)	0.41	0.046 (-0.117 to 0.210)	0.58
**Age**	0.054 (-0.180 to 0.288)	0.65	-0.059 (-0.206 to 0.089)	0.44	0.049 (-0.125 to 0.223)	0.58
**School health promotion environment category**						
**PA focus**	-0.102 (-0.360 to 0.156)	0.44	0.041 (-0.216 to 0.299)	0.75	-0.151 (-0.361 to 0.058)	0.16
**HE focus**	-0.236 (-0.480 to 0.007)	0.06	0.192 (-0.065 to 0.450)	0.14	0.067 (-0.154 to 0.289)	0.55
**Complete health focus**	0.104 (-0.137 to 0.344)	0.40	-0.009 (-0.255 to 0.238)	0.95	-0.175 (-0.377 to 0.027)	0.09

For peer review only

Table 4: Multilevel models developed for Dietary Total Energy Intake at the three study time points: all fixed effect covariates are presented

	Baseline		Follow-up one		Follow-up two	
	B (95% CI)	p-value	B (95% CI)	p-value	B (95% CI	p-value
**Trial arm**	N/A	N/A	18.5 (-364.3 to 401.4)	0.92	71.4 (-431.0 to 573.7)	0.78
**Baseline dietary total energy intake (kJ/ 24 hours)**	N/A	N/A	0.0 (0.0 to 0.1)	0.19	0.1 (0.0 to 0.1)	0.00
**Index of Multiple Deprivation score**	13.7 (-5.1 to 32.5)	0.15	2.7 (-7.6 to 13.0)	0.61	10.1 (-2.7 to 22.8)	0.12
**Female**	-586.9 (-1028.2 to -145.6)	0.01	-199.7 (-484.2 to 84.8)	0.17	-38.9 (-376.8 to 298.9)	0.82
**Ethnicity**						
**Asian**	-38.4 (-699.0 to 622.3)	0.91	-423.5 (-803.7 to -43.2)	0.03	304.4 (-153.3 to 762.0)	0.19
**African Caribbean**	439.2 (-518.6 to 1397.0)	0.37	97.2 (-520.6 to 715.1)	0.76	43.9 (-740.1 to 827.9)	0.91
**Other**	319.6 (-382.5 to 1021.7)	0.37	-505.4 (-944.4 to -66.3)	0.02	-120.8 (-654.6 to 412.9)	0.66
**Age**	621.5 (-119.3 to 1362.3)	0.10	423.3 (-49.1 to 895.6)	0.08	353.6 (-208.3 to 915.6)	0.22
**School health promotion environment category**						
**PA focus**	-903.5 (-2402.7 to 595.6)	0.24	433.7 (-70.5 to 937.9)	0.09	33.2 (-614.5 to 681.0)	0.92
**HE focus**	-847.8 (-2255.8 to 560.3)	0.24	-63.7 (-598.0 to 470.5)	0.82	260.8 (-443.1 to 964.7)	0.47
**Complete health focus**	-399.3 (-1801.3 to 1002.6)	0.58	32.8 (-464.7 to 530.3)	0.90	171.3 (-466.4 to 809.1)	0.60

For peer review only

Table 5: Multilevel models developed for Fruit and Vegetable Intake at the three study time points: all fixed effect covariates are presented

	Baseline		Follow-up one		Follow-up two	
	B (95% CI)	p-value	B (95% CI)	p-value	B (95% CI)	p-value
**Trial arm**	N/A	N/A	9.6 (-20.5 to 39.6)	0.53	-5.5 (-50.3 to 39.2)	0.81
**Baseline fruit and vegetable intake (g/ 24 hours)**	N/A	N/A	0.2 (0.1 to 0.2)	< 0.01	0.1 (0.0 to 0.1)	0.01
**Index of Multiple Deprivation score**	0.3 (-0.7 to 1.4)	0.55	-0.6 (-1.4 to 0.2)	0.15	-0.7 (-1.7 to 0.4)	0.24
**Female**	11.8 (-13.0 to 36.7)	0.35	36.9 (15.7 to 58.1)	<0.01	12.4 (-15.5 to 40.4)	0.38
**Ethnicity**						
**Asian**	17.0 (-19.9 to 53.8)	0.37	9.6 (-19.1 to 38.2)	0.51	46.5 (7.9 to 85.0)	0.02
**African Caribbean**	10.8 (-43.0 to 64.6)	0.69	64.8 (18.6 to 111.0)	0.01	33.2 (-32.1 to 98.4)	0.32
**Other**	41.6 (2.2 to 81.0)	0.04	26.7 (-6.2 to 59.6)	0.11	60.6 (16.1 to 105.1)	0.01
**Age**	38.4 (-3.4 to 80.1)	0.07	31.5 (-3.7 to 66.7)	0.08	16.2 (-30.4 to 62.7)	0.50
**School health promotion environment category**						
**PA focus**	-57.8 (-135.5 to 19.8)	0.14	11.8 (-27.9 to 51.5)	0.56	-16.0 (-73.9 to 41.9)	0.59
**HE focus**	-31.6 (-104.7 to 41.4)	0.40	3.0 (-38.6 to 44.7)	0.89	-4.4 (-66.9 to 58.0)	0.89
**Complete health focus**	-7.3 (-80.0 to 65.4)	0.84	27.1 (-11.9 to 66.0)	0.17	8.6 (-48.1 to 65.3)	0.77

For peer review only

Table 6: Multilevel models developed for Physical Activity Energy Expenditure at the three study time points: all fixed effect covariates are presented

	Baseline		Follow-up one		Follow-up two	
	**B (95% CI)**	**p-value**	**B (95% CI)**	**p-value**	**B (95% CI)**	**p-value**
**Trial arm**	N/A	N/A	0.3 (-4.6 to 5.1)	0.918	-1.7 (-6.0 to 2.6)	0.440
**Baseline PAEE (kJ/kg body weight/ 24 hours)**	N/A	N/A	0.5 (0.5 to 0.6)	< 0.001	0.4 (0.4 to 0.5)	< 0.001
**Index of Multiple Deprivation score**	0.1 (0.0 to 0.2)	0.091	-0.1 (-0.2 to 0.0)	0.093	0.0 (-0.1 to 0.1)	0.794
**Female**	-14.1 (-16.9 to -11.2)	< 0.001	-4.9 (-8.2 to -1.6)	0.004	-7.0 (-10.4 to -3.5)	< 0.001
**Ethnicity**						
**Asian**	-3.8 (-7.7 to 0.1)	0.058	-3.7 (-8.0 to 0.6)	0.093	-7.9 (-12.1 to -3.6)	< 0.001
**African Caribbean**	-17.8 (-23.8 to -11.8)	< 0.001	-6.9 (-14.0 to 0.2)	0.056	-12.8 (-21.0 to -4.6)	0.002
**Other**	-4.1 (-8.4 to 0.2)	0.064	-2.4 (-7.0 to 2.3)	0.320	-4.4 (-9.4 to 0.7)	0.093
**Age**	0.6 (-4.2 to 5.3)	0.813	2.0 (-3.2 to 7.3)	0.455	-6.6 (-12.1 to -1.1)	0.019
**School health promotion environment category**						
**PA focus**	-6.0 (-11.7 to -0.3)	0.040	-0.8 (-7.2 to 5.7)	0.811	1.9 (-3.8 to 7.6)	0.510
**HE focus**	-6.3 (-11.8 to -0.9)	0.023	3.8 (-3.1 to 10.7)	0.276	3.5 (-2.7 to 9.7)	0.269
**Complete health focus**	-6.3 (-11.6 to -1.0)	0.020	-2.6 (-8.8 to 3.6)	0.415	5.3 (0.0 to 10.6)	0.052

For peer review only

Table 7: Multilevel models developed for Estimated Time Spent in Moderate-to-Vigorous Physical Activity at the three study time points: all fixed effect covariates are presented

	Baseline		Follow-up one		Follow-up two	
	**B (95% CI)**	**p-value**	**B (95% CI)**	**p-value**	**B (95% CI)**	**p-value**
**Trial arm**	N/A	N/A	0.6 (-13.5 to 14.6)	0.94	3.8 (-7.1 to 14.7)	0.49
**Baseline estimated time spent in MVPA (min/ 24 hours)**	N/A	N/A	0.2 (0.1 to 0.3)	< 0.01	0.1 (0.1 to 0.2)	< 0.01
**Index of Multiple Deprivation score**	0.3 (0.0 to 0.5)	0.03	-0.2 (-0.5 to 0.2)	0.28	0.0 (-0.3 to 0.2)	0.72
**Female**	-13.6 (-19.6 to -7.6)	< 0.01	-13.6 (-22.4 to -4.7)	<0.01	-13.6 (-19.6 to -7.6)	< 0.01
**Ethnicity**						
**Asian**	-3.0 (-11.6 to 5.5)	0.49	-5.8 (-18.0 to 6.4)	0.35	-12.5 (-20.8 to -4.2)	<0.01
**African Caribbean**	2.6 (-10.2 to 15.5)	0.69	-3.1 (-23.1 to 17.0)	0.77	-7.3 (-22.3 to 7.6)	0.34
**Other**	5.8 (-3.4 to 15.1)	0.22	-3.6 (-16.8 to 9.6)	0.59	-3.4 (-13.0 to 6.1)	0.48
**Age**	-0.6 (-10.7 to 9.4)	0.90	8.4 (-6.4 to 23.2)	0.27	-9.1 (-19.1 to 1.0)	0.08
**School health promotion environment category**						
**PA focus**	-4.2 (-18.5 to 10.1)	0.57	-3.7 (-22.7 to 15.2)	0.70	3.8 (-10.5 to 18.2)	0.60
**HE focus**	-13.2 (-26.8 to 0.5)	0.06	-0.8 (-20.9 to 19.3)	0.94	0.8 (-14.5 to 16.1)	0.92
**Complete health focus**	-14.1 (-27.4 to -0.8)	0.04	-6.4 (-24.7 to 11.8)	0.49	7.2 (-6.4 to 20.9)	0.30

For peer review only

STROBE Statement—checklist of items that should be included in reports of observational studies

	Item No	Recommendation	Page No
**Title and abstract**	1	(*a*) Indicate the study's design with a commonly used term in the title or the abstract	1 -2
		(*b*) Provide in the abstract an informative and balanced summary of what was done and what was found	2
**Introduction**			
Background/rationale	2	Explain the scientific background and rationale for the investigation being reported	4-5
Objectives	3	State specific objectives, including any prespecified hypotheses	5
**Methods**			
Study design	4	Present key elements of study design early in the paper	6
Setting	5	Describe the setting, locations, and relevant dates, including periods of recruitment, exposure, follow-up, and data collection	6
Participants	6	(*a*) *Cohort study*—Give the eligibility criteria, and the sources and methods of selection of participants. Describe methods of follow-up<br>*Case-control study*—Give the eligibility criteria, and the sources and methods of case ascertainment and control selection. Give the rationale for the choice of cases and controls<br>*Cross-sectional study*—Give the eligibility criteria, and the sources and methods of selection of participants	6
		(*b*) *Cohort study*—For matched studies, give matching criteria and number of exposed and unexposed<br>*Case-control study*—For matched studies, give matching criteria and the number of controls per case	
Variables	7	Clearly define all outcomes, exposures, predictors, potential confounders, and effect modifiers. Give diagnostic criteria, if applicable	6-8
Data sources/ measurement	8*	For each variable of interest, give sources of data and details of methods of assessment (measurement). Describe comparability of assessment methods if there is more than one group	6-8
Bias	9	Describe any efforts to address potential sources of bias	NA
Study size	10	Explain how the study size was arrived at	NA
Quantitative variables	11	Explain how quantitative variables were handled in the analyses. If applicable, describe which groupings were chosen and why	8-9
Statistical methods	12	(*a*) Describe all statistical methods, including those used to control for confounding	9-10
		(*b*) Describe any methods used to examine subgroups and interactions	NA
		(*c*) Explain how missing data were addressed	
		(*d*) *Cohort study*—If applicable, explain how loss to follow-up was addressed<br>*Case-control study*—If applicable, explain how matching of cases and controls was addressed<br>*Cross-sectional study*—If applicable, describe analytical methods taking account of sampling strategy	10
		(*e*) Describe any sensitivity analyses	NA

Continued on next page

For peer review only - http://bmjopen.bmj.com/site/about/guidelines.xhtml

**Results**			
Participants	13*	(a) Report numbers of individuals at each stage of study—eg numbers potentially eligible, examined for eligibility, confirmed eligible, included in the study, completing follow-up, and analysed	6
		(b) Give reasons for non-participation at each stage	
		(c) Consider use of a flow diagram	
Descriptive data	14*	(a) Give characteristics of study participants (eg demographic, clinical, social) and information on exposures and potential confounders	11-15
		(b) Indicate number of participants with missing data for each variable of interest	11-14
		(c) *Cohort study*—Summarise follow-up time (eg, average and total amount)	6
Outcome data	15*	*Cohort study*—Report numbers of outcome events or summary measures over time	
		Case-control study—Report numbers in each exposure category, or summary measures of exposure	
		Cross-sectional study—Report numbers of outcome events or summary measures	
Main results	16	(*a*) Give unadjusted estimates and, if applicable, confounder-adjusted estimates and their precision (eg, 95% confidence interval). Make clear which confounders were adjusted for and why they were included	10-17
		(*b*) Report category boundaries when continuous variables were categorized	9
		(*c*) If relevant, consider translating estimates of relative risk into absolute risk for a meaningful time period	
Other analyses	17	Report other analyses done—eg analyses of subgroups and interactions, and sensitivity analyses	
**Discussion**			
Key results	18	Summarise key results with reference to study objectives	19
Limitations	19	Discuss limitations of the study, taking into account sources of potential bias or imprecision. Discuss both direction and magnitude of any potential bias	20
Interpretation	20	Give a cautious overall interpretation of results considering objectives, limitations, multiplicity of analyses, results from similar studies, and other relevant evidence	19-23
Generalisability	21	Discuss the generalisability (external validity) of the study results	19
**Other information**			
Funding	22	Give the source of funding and the role of the funders for the present study and, if applicable, for the original study on which the present article is based	24

*Give information separately for cases and controls in case-control studies and, if applicable, for exposed and unexposed groups in cohort and cross-sectional studies.

Note: An Explanation and Elaboration article discusses each checklist item and gives methodological background and published examples of transparent reporting. The STROBE checklist is best used in conjunction with this article (freely available on the Web sites of PLoS Medicine at http://www.plosmedicine.org/, Annals of Internal Medicine at http://www.annals.org/, and Epidemiology at http://www.epidem.com/). Information on the STROBE Initiative is available at www.strobe-statement.org.

For peer review only

BMJ Open

Relationship between primary school healthy eating and physical activity promoting environments and children's dietary intake, physical activity and weight status: a longitudinal study in the West Midlands, United Kingdom

Journal:	*BMJ Open*
Manuscript ID	bmjopen-2020-040833.R1
Article Type:	Original research
Date Submitted by the Author:	21-Oct-2020

Complete List of Authors:	Garden, Elizabeth; University of Birmingham, Medical School<br>Pallan, Miranda; University of Birmingham, Institute of Applied Health Research<br>Clarke, Joanne; University of Birmingham, Institute of Applied Health Research<br>Griffin, Tania ; University of Bath,<br>Hurley, Kiya; University of Birmingham, Cancer Research UK Clinical Trials Unit<br>Lancashire, Emma; University of Birmingham, Institute of Applied Health Research<br>Sitch, Alice; University of Birmingham, Institute of Applied Health Research; University Hospitals Birmingham NHS Foundation Trust, NIHR Birmingham Biomedical Research Centre<br>Passmore, Sandra; Services for Education Ltd,<br>Adab, Peymane; University of Birmingham, Institute of Applied Health Research

**Primary Subject Heading**:	Pharmacology and therapeutics
Secondary Subject Heading:	Epidemiology
Keywords:	PUBLIC HEALTH, NUTRITION & DIETETICS, PREVENTIVE MEDICINE

SCHOLARONE™
Manuscripts

I, the Submitting Author has the right to grant and does grant on behalf of all authors of the Work (as defined in the below author licence), an exclusive licence and/or a non-exclusive licence for contributions from authors who are: i) UK Crown employees; ii) where BMJ has agreed a CC-BY licence shall apply, and/or iii) in accordance with the terms applicable for US Federal Government officers or employees acting as part of their official duties; on a worldwide, perpetual, irrevocable, royalty-free basis to BMJ Publishing Group Ltd ("BMJ") its licensees and where the relevant Journal is co-owned by BMJ to the co-owners of the Journal, to publish the Work in this journal and any other BMJ products and to exploit all rights, as set out in our licence.

The Submitting Author accepts and understands that any supply made under these terms is made by BMJ to the Submitting Author unless you are acting as an employee on behalf of your employer or a postgraduate student of an affiliated institution which is paying any applicable article publishing charge ("APC") for Open Access articles. Where the Submitting Author wishes to make the Work available on an Open Access basis (and intends to pay the relevant APC), the terms of reuse of such Open Access shall be governed by a Creative Commons licence – details of these licences and which Creative Commons licence will apply to this Work are set out in our licence referred to above.

Other than as permitted in any relevant BMJ Author's Self Archiving Policies, I confirm this Work has not been accepted for publication elsewhere, is not being considered for publication elsewhere and does not duplicate material already published. I confirm all authors consent to publication of this Work and authorise the granting of this licence.

For peer review only

Relationship between primary school healthy eating and physical activity promoting environments and children's dietary intake, physical activity and weight status: a longitudinal study in the West Midlands, United Kingdom

Elizabeth Mairenn Garden, Miranda Pallan, Joanne Clarke, Tania Griffin, Kiya Hurley, Emma Lancashire, Alice Sitch, Sandra Passmore, Peymane Adab 4435

Affiliations:

Elizabeth Mairenn Garden: Student, University of Birmingham, Birmingham, UK

Miranda Pallan: Institute of Applied Health Research, University of Birmingham, Birmingham, UK

Joanne Clarke: Institute of Applied Health Research, University of Birmingham, Birmingham, UK

Tania Griffin: Department for Health, University of Bath; Bath, UK

Kiya Hurley: Cancer Clinical Trials Unit, University of Birmingham; Birmingham, UK

Emma Lancashire: Institute of Applied Health Research, University of Birmingham, Birmingham, UK

Alice Sitch: NIHR Birmingham Biomedical Research Centre, University Hospitals Birmingham NHS Foundation Trust and University of Birmingham, UK & Institute of Applied Health Research, University of Birmingham, UK

Sandra Passmore: Services for Education; Birmingham, UK

Peymane Adab: Institute of Applied Health Research, University of Birmingham, Birmingham, UK

Corresponding author:

Miranda Pallan: Institute of Applied Health Research, Murray Learning Centre, University of Birmingham, Edgbaston, Birmingham, UK. B15 2TT

Email: M.J.Pallan@bham.ac.uk

Word count: 4,435 (excluding title page, abstract, references, figures and tables)

Abstract word count: 264

For peer review only - http://bmjopen.bmj.com/site/about/guidelines.xhtml

ABSTRACT

Objectives: This study aims to examine the association between food and physical activity environments in primary schools and child anthropometric, healthy eating and physical activity measures.

Design: Secondary data analysis.

Setting: State-primary schools in the West Midlands region, UK.

Participants: 1,392 pupils who participated in the West Midlands ActiVe lifestyle and healthy Eating in School children (WAVES) childhood obesity prevention trial (2011-2015).

Outcome measures: School environment; was categorised according to questionnaire responses indicating their support for healthy eating and/or physical activity. Child measures; undertaken at 3 time-points (age 5-to-6, 7-to-8 and 8-to-9 years), included: body mass index z-scores, dietary intake (using a 24-hour food-tick-list) and physical activity (using an Actiheart monitor over 5 days).

Analyses: Primarily, associations between school food and physical activity environment categories and outcomes were explored through multilevel models at each time point.

Results: Data were available for 1,304 children (94% of the study sample). At age 8-to-9 years, children in 10 schools with healthy eating and physical activity-supportive environments had a higher physical activity energy expenditure than those in 22 schools with less supportive healthy eating/physical activity environments (mean difference=5.3kJ/kg body weight/24 hours, p=0.05). Children in schools with supportive physical activity environments (n=8) had a lower body mass index z-score than those in schools with less supportive healthy eating/physical activity environments (n=22; mean difference=-0.17, p=0.02). School food and physical activity promoting environments were not significantly associated with dietary outcomes.

Conclusions: School environments that support healthy food and physical activity behaviours may positively influence physical activity and childhood obesity.

Trial registration: The WAVES trial was registered on 19/05/2010, registration number: ISRCTN97000586.

For peer review only - http://bmjopen.bmj.com/site/about/guidelines.xhtml

KEY WORDS

Obesity, Child, Primary school, Environment, Diet, Healthy eating, Physical activity

ARTICLE SUMMARY

Strengths and limitations of this study

- The study was conducted with a large ethnically and socio-economically diverse sample of children over a 3 year period.
- Outcomes were assessed by objective measures and validated instruments, using standardised procedures.
- The school questionnaire and scoring system used to assess school food and physical activity promoting environments may not give a true representation of the extent to which schools promote health.
- Associations between weight, dietary and physical activity outcomes, and 4 categories of school food/physical activity environment were explored, but this does not fully take into account the large variation in environments seen across schools.
- The Child And Diet Evaluation Tool (CADET) only measured dietary intake over a single day, so may not be an accurate assessment of habitual intake.

For peer review only

BACKGROUND

Childhood obesity is a global public health crisis,[1] affecting countries of all income levels.[2] Since 2005 the overall trend has stabilised,[3] but its prevalence remains high in England, and a substantial increase in overweight and obesity prevalence is seen across the primary school years.[4] Approximately 23% of children age 4-to-5 years are living with overweight and obesity, and this rises to 34% by age 10-to-11 years.[4] Overweight and obesity in childhood is associated with a range of physical, psychological and social challenges,[5] and is likely to persist into adulthood.[6] It also results in significant healthcare costs, estimated to be around £6 billion per year in the UK, and projected to continue to rise in the coming years.[7] Therefore, prevention strategies targeting primary school-aged children are essential to address this significant health and economic burden.

Over the last decade, prevention strategies have shifted from focusing on individuals to a systems approach;[5,8-10] recognising the multiple interconnecting physical, economic, political, social and cultural influences. Within this context, the environments in which children are situated are critical to consider.

Schools provide a physical, social and educational environment for children and have the ability to shape physical activity (PA) and eating behaviours.[5] However, to date, our understanding of how the whole school environment influences these behaviours, and impacts on childhood obesity, is relatively limited. Research has mainly focused on evaluating specific intervention programmes introduced into schools to target weight-related behaviours. A recent Cochrane systematic review of childhood obesity prevention interventions reported that in the primary school-aged population there is some evidence to suggest that combined dietary and PA interventions in schools had a small positive effect on body mass index z-scores (mean difference between intervention and control groups of −0.05, 95% CI −0.10 to −0.01; based on meta-analysis of 20 randomised controlled trials,

For peer review only

but with low certainty of evidence).[11] However, there was no evidence to suggest that PA or dietary interventions on their own influenced body mass index z-scores.[11]

Although previous trials, using objective outcomes, suggest that school based interventions have the ability to increase PA levels;[12-15] a recent systematic review found no evidence of an effect of activity interventions on increasing whole-day moderate-to-vigorous physical activity (MVPA; standardised mean difference 0.02, 95% CI -0.07 to 0.11).[16]

2 systematic reviews have explored the effectiveness of school food environment interventions and policies on dietary behaviours. These have reported some beneficial effects, such as increased vegetable consumption, but have found no evidence of effect of these interventions on total energy intake.[13,17] However, there was significant heterogeneity of included studies in terms of the differences between schools and educational systems, which makes it challenging to draw general conclusions.

In general, whilst useful for considering specific intervention programmes designed to target diet and/or PA, the existing research evidence gives little information on how the combination of different policies, initiatives and activities in schools (which together can be termed 'the school environment') promotes PA, healthy eating (HE), and consequently impacts obesity in children. Among the few studies that have examined the relationship between school level characteristics and obesity-related behaviours, the findings have been mixed and concluded that further research is needed.[18,19]

Objectives

In this study, using data collected as part of a large childhood obesity prevention trial,[20] we aimed to explore the association between school environments promoting HE and/or PA, and

For peer review only - http://bmjopen.bmj.com/site/about/guidelines.xhtml

anthropometric, PA and dietary outcomes in UK primary school children. We hypothesised that school environments promoting HE or PA would positively influence the corresponding behavioural, and anthropometric outcomes in children.

METHODS

Design

This longitudinal, observational study uses data obtained from the West Midlands ActiVe lifestyle and healthy Eating in School children (WAVES) trial; a UK-based cluster randomised controlled trial, conducted between 2011 and 2015, that evaluated the clinical and cost-effectiveness of a 12-month obesity-prevention intervention programme delivered in primary schools.[20] The study reported here used school-level data obtained at trial baseline, and participant outcome data obtained at baseline and 2 subsequent time points.

Participants

All state primary schools in the West Midlands which included Year groups 1 to 5 and were within a 50km radius of the University of Birmingham, were eligible for inclusion in the WAVES trial. The WAVES sampling strategy sought to over-recruit schools with a high minority ethnic population through use of a weighted random sample, as the original objective of the trial was to evaluate a childhood obesity prevention intervention in an ethnically diverse population. Schools above the 80th percentile for proportion of pupils of South Asian or Black ethnicity had an increased chance of being sampled, with a 3:1 ratio. To ensure representation of a range of school characteristics, the sampling strategy was further balanced to take into account the proportion of children eligible for free school meals, school size and urban/ rural location. With this sampling method we selected 200 schools, which we randomly ordered and sequentially invited to participate in the trial. To achieve the required sample size of 54 schools, we approached 148 schools; 4 did not respond and 90 declined to participate.

For peer review only - http://bmjopen.bmj.com/site/about/guidelines.xhtml

All Year 1 pupils (age 5-to-6 years) in participating schools were eligible to take part (n=2,462 children). Parental consent was obtained for 60% of children (n=1,470), of whom 1,392 were measured at baseline (57% of those eligible). Child demographic data were obtained from parent questionnaires, or if these were not available, from school records. Home postcodes were obtained for children and mapped to Index of Multiple Deprivation 2010 scores,[21] to give an indicator of socio-economic status.

For peer review only

Pupil outcome measures

Participating pupils took part in assessments undertaken by trained researchers following standardised operating procedures. Apart from demographic data, which was only obtained at baseline, all outcome measures were obtained at 3 time points: baseline (age 5-to-6 years), follow-up-1 (age 7-to-8 years) and follow-up-2 (age 8-to-9 years).

The outcomes of interest in this study were: body mass index z-score (zBMI); waist circumference z-score (zWC) (both z-scores were calculated using UK 1990 reference curves for children to account for age and sex);[22] total energy intake (kJ/24 hours); fruit and vegetable intake (g/24 hours and portions); PA energy expenditure (PAEE; kJ/kg body weight/24 hours); and estimated time spent in MVPA (min/24 hours; defined as time spent above the acceleration threshold of 1.75 m/second).[23] Measurement instruments and processes are summarised in Table 1.

For peer review only - http://bmjopen.bmj.com/site/about/guidelines.xhtml

Table 1: Summary of measurements undertaken and their associated outcome variables

Measurement	Instrument	Number of measurements	Method of assessment	Outcome variable
**Weight (to nearest 0.1 kg)**	Tanita bioimpedance monitor (Tanita SC-331S; Tanita, Tokyo, Japan)	Once	Barefoot and in light clothing	**zBMI:** BMI was calculated by dividing weight (kg) by height (m^2). UK 1990 growth reference charts were used to produce an age- and sex-specific zBMI and define weight categories.[22] Overweight is defined as $\geq$ 85th centile and obese as $\geq$ 95th centile.[22]
**Height (to nearest 0.1 cm)**	Leicester height measure	Twice (third measure if difference >0.4cm)†	Barefoot and in light clothing	
**Child demographic data: sex, ethnicity. postcode**	Parent questionnaire (or school records if parent questionnaire not completed)	Once	Parent report or school record	
**Waist circumference (to nearest 0.1cm)**	Flexible, non-stretch, cloth tape measure	Twice (third measure if difference >0.4cm)†	Measure at iliac crest	**zWC:** calculated from WC using UK reference curves.[22]
**Dietary intake**	CADET: A validated 115 item 24-hour food tick list completed for 7 distinct time periods.[24] The CADET tool was developed for use in children aged 3-7 years.[24]	Once (24 hours)	Trained researchers recorded all food and drinks consumed by children during 1 school day. On the same day, children were given a home food diary for completion by a parent/ carer. The following day, trained researchers collected home food diaries and reviewed these with the children. When 1 or more uncompleted sections were identified, the researcher tried to complete these through child dietary recall. Data were processed through the CADET nutrient analysis program at the University of Leeds.	The food and nutrient intake data was used to calculate: **dietary total energy intake (kJ),** and **fruit and vegetable intake (g)** based on the Englyst method.[25]

For peer review only

For peer review only - http://bmjopen.bmj.com/site/about/guidelines.xhtml

**Physical activity**	Actiheart (Cambridge Neurotechnology, Papworth, UK)	Once (worn continuously for 5 days, including 1 weekend)	Attached to the child's chest with 2 ECG electrodes in school by a trained researcher, initialised to record in 30-second epochs	Total movement volume was summarised as average acceleration, along with its underlying movement intensity distribution, using the acceleration and heart rate signals. This was used to calculate: **PA energy expenditure** ¶ and **estimated time spent in MVPA.**

† Where 2 values within ≤ 0.4cm, a definitive measurement value was calculated as the average of the 2. For individuals with 3 values recorded, a definitive measurement value was calculated as the average of the closest pair (within ≤ 0.4cm), or average of all 3 reading (if there were no 2 closest readings, but the differences between values were ≤ 0.4cm). When none of the 3 values were within 0.4cm of each other, no definitive measurement value was calculated.

¶ Children with < 24 hours of valid data over the 5-day measurement period were excluded. To ensure representation across the whole 24-hour period, for those with 24 hours of valid data, only those with a distribution of at least 6 hours in each quadrant (morning 3 am – 9 am; noon 9 am – 3 pm; afternoon 3 pm – 9 pm; and midnight 9 pm – 3 am) were included.

WC, waist-circumference. CADET, Child and Diet Evaluation Tool. zBMI, body mass index z-score. BMI, body mass index. zWC, waist circumference z-score. PA, physical activity. MVPA, moderate-to-vigorous-physical -activity

For peer review only

Assessment of the school environment

Schools participating in the WAVES trial were asked to complete a baseline questionnaire, which enquired about the school's HE and PA environments. The questionnaire was developed to capture the variation in these school environments across the participating schools to assist in interpretation of the main trial findings. Question items were initially developed by the research team and then sent to a health education adviser for review. Questions were refined following feedback from the adviser. The questionnaire was completed by the head teacher or a nominated staff representative and included tick-box questions, followed by open-ended questions to gain further detail regarding policies and practices in the school.

The Health Promoting School framework requirements, government guidelines and prior research were used to develop a scoring system to categorise schools regarding their food/PA environment based on their questionnaire responses. (Supplementary File Table 1).[17,26-29] The scoring system was developed in an iterative way, by the study team, until a consensus was reached on the scoring method and weighting. The use of government publications ensured that the scoring system encompassed key items that have been determined as important for national policy relating to child health at school (for example the provision of 2 hours or more of physical education (PE) per week).[29]

Each question contributed to the final scores. Questions with continuous response variables (such as number of active after school clubs on offer), were categorised into 3 groups: high; medium; low, using tertile cut-offs with a score allocation of 2;1;0, respectively. Questions with categorical responses were either ordinal or binary (yes/no) questions. Binary questions, such as schools having a healthy eating policy, were scored as 2/0. For the ordinal variables: schools allocating more than 2 hours to PE was considered of high importance, they were grouped into schools providing this for all years; some years; no years with a score of 2;1;0; while level of support from stakeholders

For peer review only - http://bmjopen.bmj.com/site/about/guidelines.xhtml

(strongly support; support; weakly support) was deemed as less important, so was scored out of 1 (1;0.67;0.33 respectively).

We developed separate HE and PA scores based on a sum of scores from 15 and 20 questions (range of possible scores 44%-92% and 24%-92%), respectively. Using the range of scores across all schools, we used tertile cut-offs to split HE and PA scores into 3 groups. Schools were then categorised according to their environment: i) complete health focus, supporting both HE and PA (top tertile for both HE and PA scores); ii) HE focus (top tertile for HE scores only); iii) PA focus (top tertile for PA scores only); iv) minimal health focus (not in the top tertile for either HE or PA scores).

Data analysis

All analyses were undertaken using STATA Version 15.[30] Descriptive analyses were used to describe pupil characteristics at baseline and both follow-up time points. Categorical variables were summarised as numbers (percentage). Continuous data were summarised as mean (standard deviation) and median (interquartile range (IQR)), as appropriate.

Due to the clustered nature of the data, multilevel linear regression models were used, with school included as a random effect. Primary analysis explored the relationship between the school environment category and pupil outcome measures at the 3-study time-points separately. Additionally, a model was fitted using all time-points, allowing for the repeated measures and including a time covariate. Variables included in the model were: baseline age, sex, ethnicity, Index of Multiple Deprivation score and school environment category. Models of follow-up time-points included adjustment for baseline outcome value and the arm of the WAVES trial. The trial arm covariate refers to whether schools were allocated to intervention or control arms in the main WAVES trial. Trial arm was not included in the baseline analyses as participant outcome data at this time point were collected before trial arm allocation. Model assumptions were checked using

For peer review only - http://bmjopen.bmj.com/site/about/guidelines.xhtml

For peer review only

residual plots and the appropriate inclusion of the school random effect was confirmed through likelihood ratio tests. Covariate data were missing for less than 5% of participants, therefore imputation was not performed and a complete case analysis was used.

Patient and public involvement

The WAVES trial research team included a health education advisor (SP) who advised on engagement with schools and school questionnaire design. Input was sought from a head teacher on methods of participant recruitment and participant information materials. The oversight committee for the trial also included a head teacher, who advised on trial conduct throughout.

RESULTS

Of the 54 schools participating in the WAVES trial, 50 (93%) returned the baseline questionnaire. It is these schools and the pupil participants attending them (n=1,304, 94% of the WAVES trial sample) that were included in the current analyses.

Pupil characteristics

Baseline characteristics for pupils are shown in Table 2. The sociodemographic characteristics of the WAVES trial participants excluded from these analyses (due to non-return of school questionnaires) were compared to the study sample and did not differ significantly by age or gender. Those excluded from the analyses were less likely to be of White British ethnicity, and more likely to be of South Asian ethnicity, or in the most deprived Index of Multiple Deprivation score quintile. Anthropometric, dietary and PA characteristics did not differ between those included and those excluded from the analyses.

For peer review only - http://bmjopen.bmj.com/site/about/guidelines.xhtml
For peer review only

Table 2: Summary of pupil demographic information at baseline

	Baseline value for those analysed in this study (n=1304)
**Age in years: mean (SD) ; missing data (n)***	6.3 (0.31); 61
**Gender: n (%)**	
**Boys**	654 (50.2)
**Girls**	650 (49.9)
**Missing data***	0
**Ethnicity: n (%)**	
**White British**	656 (50.8)
**South Asian**	333 (25.8)
**African-Caribbean**	100 (7.8)
**Other**	202 (15.7)
**Missing data* (n)**	13
**Deprivation quintile: n (%)†**	
**1 (most deprived)**	639 (50.0)
**2**	266 (20.8)
**3**	146 (11.4)
**4**	119 (9.3)
**5 (least deprived)**	109 (8.5)
**Missing data* (n)**	25
**Deprivation score: mean (SD); missing data (n)***	33.8 (17.9); 25

* Missing data values are not included in the denominator.

† Index of multiple deprivation scores categorised into 5 groups using the national quintile cut-offs for England.

IQR, interquartile range.

Outcome measures are summarised for all 3 time-points in Table 3. Missing data at follow-up 1 and 2 are in part due to attrition of participants as the WAVES trial progressed. The main reasons for attrition were pupils moving away from the school or being absent on the day of data collection. The prevalence of overweight/obesity in the study population was 20.8% at baseline, 26.8% at follow-up-1 (15 months) and 31.1% at follow-up-2 (30 months). Prevalence varied across schools from 8–32% at baseline, 9–47% at follow-up-1 and 9–53% at follow-up-2. There was a statistically significant increase between baseline and follow-up-2 for zBMI and zWC ($p<0.001$). As would be expected, total energy intake increased over the study period as the participating children grew and increased their energy requirement. However, a statistically significant decrease in PAEE and estimated time spent in MVPA ($p<0.001$) was also observed during this period. There was no significant difference in fruit and vegetable intake over time ($p=0.20$).

For peer review only - http://bmjopen.bmj.com/site/about/guidelines.xhtml

Table 3: Summary of pupil outcome measures at 3 timepoints

	Baseline (mean (SD) age = 6.3 (0.3) years)	Follow-up 1 (mean (SD) age = 7.7 (0.3) years)	Follow-up 2 (mean (SD) age = 9.0 (0.3) years)
**Anthropometric**			
**Mean (SD) zBMI; missing data***	0.21 (1.2); 64	0.32 (1.3); 201	0.39(1.3); 293
**BMI group category: n (%)†**			
**Underweight (≤2nd centile)**	29 (2.3)	23 (2.1)	23 (2.3)
**Healthy (>2nd and <85th centiles)**	953 (76.9)	784 (71.1)	674 (66.7)
**Overweight (≥85th and <95th centiles)**	109 (8.8)	126 (11.4)	117 (11.6)
**Obese (≥95th centile)**	149 (12.0)	170 (15.4)	197 (19.5)
**Missing data***	64	201	293
**Median (IQR) zWC; missing data***	0.76 (1.2); 167	0.99 (1.3); 275	1.1 (1.3); 416
**Dietary intake**			
**Median (IQR) dietary total energy intake (kJ/ 24 hours); missing data***	6976.9 (5933.9 – 8101.8); 212	7150.3 (6100.0 – 8279.0); 301	7728.8 (6595.0 – 9128.9); 393
**Median (IQR) fruit and vegetable intake (g/ 24 hours); missing data***	241.8 (149.6 – 352.6); 212	207.8 (111.5 – 320.2); 301	219.5 (117.0 – 346.5); 393
**≥ 5 portion fruit and vegetables: n (%)**			
**Yes**	705 (64.6)	502 (50.1)	519 (57.0)
**No**	387 (35.4)	501 (50.0)	392 (43.0)
**Missing data***	212	301	393
**Physical activity**			
**Mean (SD) PAEE (kJ/kg body weight/ 24 hours); missing data***	94.8 (23.8); 341	91.5 (24.5); 521	79.4 (22.4); 680
**Median (IQR) estimated time spent in MVPA (min/ 24 hours); missing data***	58.1 (41.9 – 81.7); 345	62.0 (39.8 – 101.9); 523	43.1 (31.6 – 64.2); 672

* Missing data values are not included in the denominator.

† Based on UK 1990 reference centile curves and applying cut-offs used for population monitoring.

IQR, interquartile range. zBMI, body mass index z-score. BMI, body mass index. zWC, waist circumference z-score. PAEE, physical activity energy expenditure. MVPA, moderate-to-vigorous physical activity.

For peer review only - http://bmjopen.bmj.com/site/about/guidelines.xhtml

School characteristics

All schools had some activities or policies that promoted HE or PA. Overall schools gained higher scores for PA than HE promotion, with the mean normalised score (adjusted for number and type of questions) being 76% (IQR: 66–85%) for PA and 67% (IQR: 51–74%) for HE. 8 (16%) schools were classed as PA focus, 10 (20%) as HE focus, 10 (20%) as complete health focus and 22 (44%) as minimal health focus. The application of the scoring system to schools based on their baseline questionnaire responses, including information on missing data, is shown in Table 4.

For peer review only

Table 4: Summary of the scoring of schools on healthy eating and physical activity based on baseline questionnaire responses

Description of variable	Number of schools (%)		
**Healthy eating score**			
	**No (score=0)**	**Yes (score =2)**	
School has a policy regarding HE*	11 (22.5)	38 (77.6)	
HE is promoted in school curriculum	10 (20.0)	40 (80.0)	
School cookery club to promote HE	29 (58.0)	21 (42.0)	
Involve parents in the promotion of HE	27 (54.0)	23 (46.0)	
Drinking water is provided throughout the day*	0 (0.0)	49 (100.0)	
Breakfast club is available for students	15 (30.0)	35 (70.0)	
Food provided by the school at break deemed as healthy*	32 (65.3)	17 (34.7)	
	**Low (score=0)**	**Medium (score =1)**	**High (score =2)**
Provide a range of ways to promote HE	12 (24.0)	13 (26.0)	25 (50.0)
Proportion of children choosing to eat school lunches†	12 (32.4)	11 (29.7)	14 (37.8)
	**Weakly support (score=0.33)**	**Support (score=0.67)**	**Strongly support (score=1)**
Governors support HE in school	2 (4.0)	31 (62.0)	17 (34.0)
Senior Leadership Team support HE in school*	0 (0.0)	21 (42.9)	28 (57.1)
Staff support HE in school*	0 (0.0)	27 (55.1)	22 (44.9)
School council support HE in school*	2 (4.1)	25 (51.0)	22 (44.9)
Pupils support HE in school*	5 (10.2)	35 (71.4)	9 (18.4)
Parents support HE in school*	14 (28.6)	31 (63.3)	4 (8.2)

Description of variable	Number of schools (%)		
**Physical activity score**			
	**No (score=0)**	**Yes (score=2)**	
School has a policy regarding PA**	3 (6.3)	45 (93.8)	
School has a PA co-ordinator***	4 (8.5)	43 (91.5)	
Staff are trained regarding PA***	17 (36.2)	30 (63.8)	
Has sport partnerships and community links***	11 (23.4)	36 (76.6)	
PA is promoted in the school curriculum**	6 (12.5)	42 (87.5)	
Involve parents in the promotion of PA**	32 (66.7)	16 (33.3)	
School has a walk to school campaign**	13 (27.1)	35 (72.9)	
PE is taught by a specialist**	21 (43.8)	27 (56.3)	
Range of activities offered in PE meet Department of Education recommendations**	3 (6.3)	45 (93.8)	
	**Low (score=0)**	**Medium (score=1)**	**High (score=2)**
Provide a range of ways to promote PA**	7 (14.6)	20 (41.7)	21 (43.8)
Variety of PA opportunities other than PE**	10 (20.8)	15 (31.3)	23 (47.9)
Have a range of play equipment to be used**	12 (25.0)	18 (37.5)	18 (37.5)
Provide a range of sports clubs**	12 (25.0)	11 (22.9)	25 (52.1)
	**For no years (score=0)**	**For some years (score=1)**	**For all years (score=2)**
Schools allocating 2 or more hours to PE per week **	7 (14.6)	2 (4.2)	39 (81.3)
	**Weakly support (score=0.33)**	**Support (score=0.67)**	**Strongly support (score=1)**
Governors support PA in school**	0 (0.0)	28 (58.3)	20 (41.7)
Senior Leadership Team support PA in school**	0 (0.0)	18 (37.5)	30 (62.5)
Staff support PA in school**	0 (0.0)	22 (45.8)	26 (54.2)
School council support PA in school***	0 (0.0)	24 (51.1)	23 (48.9)
Pupils support PA in school**	0 (0.0)	22 (45.8)	26 (54.2)
Parents support PA in school**	4 (8.3)	33 (68.8)	11 (22.9)

* Missing data from 1 school, ** Missing data from 2 schools, *** Missing data from 3 schools, † Missing data from 13 schools.

Missing data values are not included in the denominator used for percentage calculations.

HE, healthy eating. PA, physical activity. PE, Physical Education.

Not all schools reported having HE or PA policies (11 (22%) with no HE policy; 3 (6%) with no PA policy); however, several of these schools were placed in the PA focus, HE focus or complete health focus categories as they scored highly in other aspects of healthy food and PA promotion activity. All schools provided drinking water throughout the school day. 35 (70%) had a breakfast

For peer review only - http://bmjopen.bmj.com/site/about/guidelines.xhtml

club available to students. However, it was difficult to assess how healthy the food provided was from the information available and only 17 schools (35%) provided a healthy break snack. 45 schools (94%) offered a range of activities during PE that met the Department of Education recommendations and 39 (81%) allocated 2 or more hours to PE per week. However, PE was taught by a specialist in only 27 schools (56%). Parents were perceived to support the HE and PA environment less than other stakeholders in the school. In addition, only 23 (46%) and 16 (33%) of schools involved parents in HE and PA promotion, respectively.

Associations between the school food/PA environment category and childhood anthropometric, diet and PA outcomes

For all models (i.e. models at each time-point and repeated measures models for each outcome), the regression coefficients (B) for the school environment categories are presented in Table 5, with the minimal health focus category as the reference. Regression coefficients for all fixed-effect covariates in the models are presented in Supplementary File Tables 2-8. School environment category was not statistically significantly associated with child zBMI at baseline or follow-up-1. At follow-up-2, compared with minimal health focus schools, pupils in PA focus schools had a lower zBMI (B=-0.17, 95% CI -0.31 to -0.03, p=0.02). Although not statistically significant, compared to those in schools with minimal health focus, children in schools with a complete health focus also had a lower zBMI at follow-up-2 (B=-0.11, 95% CI -0.25 to 0.03, p=0.14). At follow-up-2, PA focus and complete health focus schools also had a lower zWC than minimal health focus schools, but this was non-significant (PA focus: B=-0.15, 95% CI -0.36 to 0.06, p=0.16 and complete health focus: B=-0.18, 95% CI -0.38 to 0.03, p=0.09). Compared with minimal health focus schools, PA focus and the HE focus schools had lower zBMI and zWC in the repeated measures models, but these were non-significant.

For peer review only - http://bmjopen.bmj.com/site/about/guidelines.xhtml

Children in schools in HE, PA and complete health focus categories had significantly lower daily PAEE at baseline than those in minimal health focus schools (PA focus: B=-6.0, 95% CI -11.7 to -0.28, p=0.04; HE focus: B=- 6.3, 95% CI -11.8 to – 0.86, p=0.02; and complete health focus: B=-6.3, 95% CI -11.6 to -1.0, p=0.02). They also did less MVPA at baseline, however, this was only significant for schools with a complete health focus (B=-14.1, 95% CI -27.4 to -0.75, p=0.04). By follow-up-2, this trend reversed, with higher levels of both PAEE and MVPA in the HE, PA and complete health focus schools, compared with minimal health focus schools, although these findings were not significant. We found no significant differences in PAEE across school environment categories in the repeated measures model.

School environment category was not significantly associated with total dietary energy intake or fruit and vegetable intake in any of the models.

For peer review only

Table 5: The association between the school food/physical activity environment category and pupil obesity, dietary and physical activity outcome measures, estimated using multilevel modelling

Outcome variable	School environment category *	Outcome time point							
		Baseline		Follow-up-1		Follow-up-2		Repeated measures model	
		Regression coefficient (95% CI)	p-value	Regression coefficient (95% CI)	p-value	Regression coefficient (95% CI)	p-value	Regression coefficient (95% CI)	p-value
**zBMI**	**PA focus**	-0.06 (-0.25, 0.14)	0.57	-0.06 (-0.22, 0.10)	0.48	**-0.17 (-0.31, -0.03)**	**0.02**	-0.10 (-0.29, 0.10)	0.32
	**HE focus**	-0.09 (-0.28, 0.09)	0.33	-0.02 (-0.18, 0.15)	0.86	-0.02 (-0.16, 0.13)	0.80	-0.07 (-0.27, 0.13)	0.47
	**Complete health focus**	0.04 (-0.14, 0.23)	0.64	0.07 (-0.09, 0.23)	0.41	-0.11 (-0.25, 0.03)	0.14	0.07 (-0.12, 0.26)	0.49
**zWC**	**PA focus**	-0.10 (-0.36, 0.16)	0.44	0.04 (-0.22, 0.30)	0.75	-0.15 (-0.36, 0.06)	0.16	-0.11 (-0.32, 0.11)	0.33
	**HE focus**	-0.24 (-0.48, 0.01)	0.06	0.19 (-0.07, 0.45)	0.14	0.07 (-0.15, 0.29)	0.55	-0.14 (-0.37, 0.08)	0.21
	**Complete health focus**	0.10 (-0.14, 0.34)	0.40	-0.01 (-0.26, 0.24)	0.95	-0.18 (-0.38, 0.03)	0.09	0.07 (-0.14, 0.28)	0.52
**Dietary total energy intake (kJ/24 hours)**	**PA focus**	-903.5 (-2402.7, 595.6)	0.24	433.7 (-70.5, 937.9)	0.09	33.2 (-614.5, 681.0)	0.92	-220.9 (-815.1, 373.4)	0.47
	**HE focus**	-847.8 (-2255.8, 560.3)	0.24	-63.7 (-598.0, 470.5)	0.82	260.8 (-443.1, 964.7)	0.47	-483.8 (-1087.4, 119.9)	0.12
	**Complete health focus**	-399.3 (-1801.3, 1002.6)	0.58	32.8 (-464.7, 530.3)	0.90	171.3 (-466.4, 809.1)	0.60	-200.0 (-773.9, 373.8)	0.49
**Fruit and vegetable intake (g/24 hours)**	**PA focus**	-57.8 (-135.5, 19.8)	0.14	11.8 (-27.9, 51.5)	0.56	-16.0 (-73.9, 41.9)	0.59	-22.6 (-64.9, 19.6)	0.29
	**HE focus**	-31.6 (-104.7, 41.4)	0.40	3.0 (-38.6, 44.7)	0.89	-4.4 (-66.9, 58.0)	0.89	-21.6 (-64.4, 21.2)	0.32
	**Complete health focus**	-7.3 (-80.0, 65.4)	0.84	27.1 (-11.9, 66.0)	0.17	8.6 (-48.1, 65.3)	0.77	5.2 (-35.6, 45.9)	0.80

For peer review only - http://bmjopen.bmj.com/site/about/guidelines.xhtml

Outcome variable	School environment category *	Outcome time point							
		Baseline		Follow-up-1		Follow-up-2		Repeated measures model	
		Regression coefficient (95% CI)	p-value	Regression coefficient (95% CI)	p-value	Regression coefficient (95% CI)	p-value	Regression coefficient (95% CI)	p-value
PAEE (kJ/kg body weight/24 hours)	PA focus	**-6.0 (-11.7, -0.28)**	**0.04**	-0.79 (-7.2, 5.7)	0.81	1.9 (-3.8, 7.6)	0.51	-2.8 (-7.2, 1.6)	0.21
	HE focus	**-6.3 (-11.8, -0.86)**	**0.02**	3.8 (-3.1, 10.7)	0.28	3.5 (-2.7, 9.7)	0.27	-0.83 (-5.3, 3.7)	0.72
	Complete health focus	**-6.3 (-11.6, -1.0)**	**0.02**	-2.6 (-8.8, 3.6)	0.42	**5.3 (0.04, 10.6)**	**0.05**	-3.0 (-7.2, 1.2)	0.16
Estimated time spent in MVPA (min/24 hours)	PA focus	-4.2 (-18.5, 10.1)	0.57	-3.7 (-22.7, 15.2)	0.70	3.8 (-10.5, 18.2)	0.60	-1.4 (-11.9, 9.1)	0.80
	HE focus	-13.2 (-26.8, 0.47)	0.06	-0.80 (-20.9, 19.3)	0.94	0.78 (-14.5, 16.1)	0.92	-5.0 (-15.7, 5.7)	0.36
	Complete health focus	**-14.1 (-27.4, -0.75)**	**0.04**	-6.4 (-24.7, 11.8)	0.49	7.2 (-6.4, 20.9)	0.30	-6.4 (-16.5, 3.7)	0.21

* Minimal health focus schools are the reference category.

All models with outcomes measured at a single time point include school as a random effect and age at baseline, sex, ethnicity, and index of multiple deprivation score as fixed-effect covariates. Models with outcomes at follow-up-1 and follow-up-2 also include the outcome variable at baseline and the WAVES trial arm as fixed-effect covariates.

Repeated measures models include school and pupil as a random effect, and measurement time point, age at baseline, sex, ethnicity, index of multiple deprivation score and WAVES trial arm as fixed-effect covariates.

Results shown in bold are statistically significant.

zBMI, body mass index z-score. zWC, waist circumference z-score. PAEE, physical activity energy expenditure. MVPA, moderate-to-vigorous physical activity. PA, physical activity. HE, healthy eating.

For peer review only

For peer review only - http://bmjopen.bmj.com/site/about/guidelines.xhtml

DISCUSSION

The questionnaire responses indicated that all schools promoted healthy behaviours to some degree, but there was variation in the extent of their efforts. The school scores indicated that schools tended to focus more on promoting PA than HE. This may relate to increased government focus and spending on PA in schools during the study period. Overall, we found little relationship between the school food/PA environment and pupil anthropometric measures, diet or PA levels. Most associations between the school environment category and the outcome measures were non-significant, and those that were significant were small in magnitude. However, by the second follow-up time point (30 months after baseline) there was a statistically significant lower mean zBMI among pupils in schools with a PA focus, compared with those in schools with minimal health focus. Although the effect size is small (mean difference= -0.17; 95% CI -0.31 to -0.03; p= 0.02), if successfully sustained it has the potential to produce public health benefits at a population level.[31] A similar finding was observed for zWC, although this was non-significant. In line with these associations between school environment activity and obesity-related outcomes, we found that at the second follow-up time point PAEE levels were higher in schools with a complete health focus compared with those with minimal health focus. Therefore, the findings may suggest that a healthy school environment could have an effect over time. In this study, we used baseline data collected when the participants had already been at school for a year, so it is possible that if we had baseline data from before school entry, we would have seen a larger association between school environment and obesity and physical activity outcomes.

Strengths

To our knowledge, this is the first UK study to assess the association between primary school food and PA promoting environments and child obesity measures longitudinally. The study involves a large sample, from an ethnically and socio-economically diverse population, over a 3-year period. Outcomes were predominantly assessed by objective measures, using validated instruments and

For peer review only - http://bmjopen.bmj.com/site/about/guidelines.xhtml

standardised procedures. Clustering and individual level confounders were accounted for by using multilevel models, which enables more confidence in the observed associations between the school food and PA promoting environment and the outcomes.

Limitations

The sample includes only 50 primary schools that consented to be part of an obesity prevention trial and parental consent was only obtained for 60% of eligible children within these schools. Our study sample may therefore represent schools with more interest in health promotion and pupils who were healthier. However, obesity prevalence in the participating schools was compared with the prevalence estimated from the National Child Measurement Programme at the time and was found to be similar.[32] Comparisons of demographic characteristics of children with and without parental consent to participate in the WAVES trial (undertaken as part of the WAVES trial) also did not show major differences,[33] suggesting that the study sample is broadly representative.

Further limitations relate to participant outcome measurement and attrition. There was attrition between each measurement time point. A higher proportion of participants of African/Caribbean ethnicity and participants from more deprived groups had missing outcome data at the follow up time points, which may partially account for different findings in the follow-up models compared with the baseline models.

The questionnaire used to assess the school environment was developed by the WAVES trial research team with input from a health education adviser to capture variation in the environments of the participating schools. It was assessed for face validity, but not further validated. Schools completed the questionnaire with a varying degree of completeness; therefore, the resulting information may not fully reflect their activity relating to the promotion of healthy eating and PA. The methods for scoring schools in relation to HE and PA were developed by the authors, based on past and present guidelines relating to health promoting schools. However, the construct validity of

For peer review only - http://bmjopen.bmj.com/site/about/guidelines.xhtml

these could not be meaningfully explored through confirmatory factor analysis due to the small number of schools in our sample.[34] Furthermore, the categorisation process of schools was pragmatically determined based on statistical cut-offs, and there may be large variations in health promoting activities within each of the categories. It is possible that, due to limitations of the baseline questionnaire and the lack of validation of the scoring system, the schools' food and PA promoting environments may not have been accurately represented, which in turn may have led to the equivocal findings of the study. In addition, the school environment was only assessed at 1 time point, and it is possible that support for HE or PA in the schools changed over time. Therefore, we have not been able to assess longitudinal changes in the school environment and how these are associated with obesity, diet and PA outcomes.

We explored associations between the school environment and multiple outcomes in this study. It is therefore possible that the statistically significant associations that we found could be due to multiple testing. However, as this was an exploratory, hypothesis generating study, we did not apply a correction for multiple testing as these tend to be conservative and may have led us to miss potential differences between groups that warrant further research.[35] The Child And Diet Evaluation Tool (CADET) was quick and practical with a relatively low response burden,[36] but it only measured dietary intake over a single day, so may not be an accurate assessment of habitual intake.

Finally, the data used in this study were drawn from a randomised controlled trial evaluating a childhood obesity prevention intervention and included data from both intervention and control arms. Although there was no evidence of effectiveness of the intervention from the main trial,[20] we adjusted for the intervention/control status of participants in our analyses. However, even with these adjustments we cannot rule out the possibility of marginal intervention effects influencing the

For peer review only - http://bmjopen.bmj.com/site/about/guidelines.xhtml

For peer review only

association between school food and PA environments and anthropometric, physical activity and dietary outcomes.

Comparison with previous research

This study brings new information regarding the potential impact the school HE and PA environment has on children, as previous research has focused on the effectiveness of specific interventions and policies within schools.

Our findings are in line with those from 2 systematic reviews that found moderate evidence that school PA interventions are able to reduce BMI.[11,13] Our study included a range of school level activities, rather than focusing on specific interventions, and the effect size observed at 30 months was similar to the summary estimate from the trials.[11]

There is a large pool of research indicating PA interventions increase PA levels in children during the school day,[12-15] although overall PA levels may not be increased.[16] We found that children in schools with an increased focus on PA were less active and engaged in less MVPA at baseline compared with minimal health focus schools, but over time the trend was reversed, suggesting that school PA promotion may increase child PA levels over time. Our findings may also indicate that PA promotion in schools only affects PA levels as children get older, when their PA levels generally decrease.

Previous systematic reviews have not found promising results regarding the impact of interventions on dietary outcomes, although there is some evidence that they are able to increase fruit and vegetable intake.[13,17] This study did not find a relationship between the school environment and child dietary outcomes. The differences between the results of this study and previous research may be as a result of assessing overall environment, rather than specific targeted interventions. In 2018,

For peer review only - http://bmjopen.bmj.com/site/about/guidelines.xhtml

the House of Commons (UK Parliament) stated that there was no convincing evidence the school healthy food standards had been implemented effectively.[37] The majority (n=32, 65%) of schools in this study provided a break-time snack which was deemed unhealthy, suggesting other school food provision may also be unhealthy. Furthermore, the limitations of the questionnaire in assessing school healthy eating environment and those related to the dietary assessment method may also have contributed to differences in findings.

In terms of healthy food and PA promoting environments, we found that school perception of parental support for HE in schools was not high (low parental support perceived by over a quarter of schools). Children consistently refer to home life being more significant in changing behaviour than schools and families have been shown to be pivotal in ensuring improvements to lifestyle in primary school aged children.[38-40] Our findings suggest that in relation to PA and HE, parent-school relationships need strengthening. Nevertheless, the complexity of promoting a healthy school environment and of including parents in such endeavours also need to be considered.[41]

Implications

Consistent with previous research, this study indicates that supporting PA in primary schools may have beneficial health effects in terms of increasing PA levels and reducing weight status. Although schools often prioritise academic attainment over PE and PA, increasing PE in the curriculum has not been found to have negative academic consequences and may improve school achievement.[42,43] Therefore, schools are recommended to increase their efforts to promote PA.

In this study, school environments were not as supportive of HE as expected, which is in line with UK Parliament's report stating that there was a lack of effective implementation of school food standards legislation.[37] Stronger enforcement of existing school food regulation and further regulatory measures may be needed to ensure HE is fully supported in schools.

For peer review only - http://bmjopen.bmj.com/site/about/guidelines.xhtml

All associations between the school food/PA environment and health/behavioural outcomes were of small magnitude, which suggests it has an important but limited role in child health. Parents are key influencers of children's health,[39,40] but in this study the majority of schools did not involve parents in their health promotion efforts. The development of school food and PA promoting environments may be more effective if schools develop better relationships with parents and involve them in their health promotion efforts.

Conclusion

We found that over time, a school environment supportive of PA may positively influence zBMI and that schools with a more food and PA promoting environment may contribute to increased PA levels in children over time. More research is needed to further understand the influence of the school environment, but in the meantime, schools should be encouraged to provide an environment that is supportive of PA and HE. However, schools cannot undertake health promotion in isolation. They need to be considered as part of a complex system, which requires intervention at multiple points to produce a sustained reduction in child overweight and obesity.

For peer review only

For peer review only - http://bmjopen.bmj.com/site/about/guidelines.xhtml

ACKNOWLEDGEMENTS

We would like to acknowledge the contribution of all the WAVES study trial investigators: University of Birmingham: Peymane Adab (Professor of Chronic Disease Epidemiology & Public Health and Chief Investigator), Tim Barrett (Leonard Parsons Professor of Paediatrics and Child Health), KK Cheng (Professor of Public Health and Primary Care), Jonathan J Deeks (Professor of Biostatistics), Joan L Duda (Professor of Sport and Exercise Psychology), Emma Frew (Professor in Health Economics), Karla Hemming (Professor of Biostatistics), Miranda J Pallan (Reader in Public Health & Epidemiology), Jayne Parry (Professor of Policy and Public Health); University of Cambridge, Cambridge MRC Epidemiology Unit / Norwegian School of Sport Sciences: Ulf Ekelund (Professor in Physical Activity and Health); University of Leeds: Janet E Cade (Professor of Nutritional Epidemiology and Public Health); Loughborough University: Amanda Daley (Professor of Behavioural Medicine); University of Edinburgh: Raj Bhopal (Emeritus Professor of Public Health); University of Warwick: Paramjit Gill (Professor of General Practice); Birmingham Community Healthcare NHS Trust: Eleanor McGee (Public Health Nutrition Lead); Birmingham Services for Education: Sandra Passmore (Education Advisor)

We thank the children, school staff, and parents who participated in the trial; the study team, including Behnoush Mohammadpoor Ahranjani and Emma Popo who helped in overseeing the study measurements and data collection; the administrative team who facilitated the running of the project; the research staff who undertook the study measurements; and Robert Lancashire who developed the trial database and oversaw data management. We also thank our collaborators at the Institute of Metabolic Science at the Medical Research Council (MRC) Epidemiology Unit (University of Cambridge) for their support and assistance, particularly Soren Brage, Kate Westgate, and Stefanie Hollidge, for training of field staff on use and maintenance of Actihearts, for overseeing data preparation, and for analysis and interpretation of the physical activity data; Timo Lakka (principal investigator of the PANIC study, University of Eastern Finland) for sharing

For peer review only - http://bmjopen.bmj.com/site/about/guidelines.xhtml

exercise data in a similarly aged cohort of children to allow translation of observed heart rate into activity energy expenditure; staff who are working, or have previously worked, at the Nutrition Epidemiology Group in Leeds, who have supported the trial team in terms of administration of the CADET food tick list and processing of the data to enable us to undertake dietary intake analysis, in particular Neil Hancock, Cristina Cleghorn, Meagan Christian, Jayne Hutchinson, Holly Rippin, and Catherine Rycroft. We also thank Sayeed Haque for his statistical advice on the analyses for this particular study.

HUMAN SUBJECTS APPROVAL STATEMENT

The WAVES trial was approved by NHS Research Ethics Service Committee West Midlands, The Black Country (NHS REC Number: 10/H1202/69) and sponsored by the University of Birmingham (Sponsor Number: RG_10-183). Parents gave written informed consent for children to participate and children gave verbal assent before measurements were taken. Permission to access the WAVES study data for this study was obtained in October 2018.

TRIAL MONITORING AND DATA MONITORING

The trial steering committee met annually and included: chair/statistician: Kelvin Jordan, Keele University; subject expert: Peter Whincup, St George's, University of London; health economist: Louise Longworth, Brunel University; public representative: John Bennett, PHSE advisor; and Chief Investigator: Peymane Adab, University of Birmingham.

FUNDING

This WAVES trial was funded by the National Institute for Health Research (NIHR) Health Technology Assessment Programme (project reference 06/85/11). The views expressed are those of the authors and not necessarily those of the NIHR or the Department of Health and Social Care.

For peer review only - http://bmjopen.bmj.com/site/about/guidelines.xhtml

For peer review only

COMPETING INTERESTS

Miranda Pallan, Tania Griffin, Kiya Hurley, Emma Lancashire, Alice Sitch and Peymane Adab hold grants from the UK National Institute for Health Research, and Peymane Adab is Chair of the National Institute for Health Research Public Health Research Funding Committee. There are no other competing interests to declare.

AUTHOR CONTRIBUTION

Elizabeth Mairenn Garden (first author) designed and undertook the analysis reported in this manuscript, and drafted the manuscript. Miranda Pallan and Peymane Adab designed and oversaw the conduct of the trial from which the data is drawn, advised on the design and analysis of the reported study, and also contributed substantially to the manuscript. Joanne Clarke, Tania Griffin, Kiya Hurley and Emma Lancashire all designed school and pupil data collection tools and undertook data collection. They also substantially contributed to the manuscript. Alice Sitch assisted in developing the analysis plan, conducted parts of the analyses and substantially contributed to the manuscript. Sandra Passmore oversaw the main trial from which the data is drawn, advised on the development of the school data collection tool, and substantially contributed to the manuscript, bringing a school policy perspective. All authors approved the final version of the manuscript.

DATA SHARING STATEMENT

Requests for access to data from the WAVES trial should be addressed to the Principal Investigator at p.adab@bham.ac.uk. All proposals requesting data access will need to specify how it is planned to use the data, and all proposals will need approval of the trial co-investigator team before data release.

For peer review only

REFERENCES

1. Mead E, Brown T, Rees K, et al. Diet, physical activity and behavioural interventions for the treatment of overweight or obese children from the age of 6 to 11 years. *Cochrane Database Syst Rev* 2017; Issue 6. Art No.:CD012651 doi:10.1002/14651858.CD012651.
2. Sahoo K, Sahoo B, Choudhury AK, et al. Childhood obesity: causes and consequences. *J Family Med Prim Care* 2015;4(2):187–92 doi:10.4103/2249-4863.154628.
3. Rutter H. Overweigh and obesity: where are we and where are we heading?. National Obesity Observatory. 2012. Available from: https://www.england.nhs.uk/wp-content/uploads/2012/05/Obesity-Review-Group-19-April-2012-Background-Slide-Set-1-Prevalence-and-Trends.pdf. Accessed: 10 Oct 2020.
4. Stats Team. National child measurement programme England, 2017/ 2018 School Year. 2018. Available from: https://files.digital.nhs.uk/9F/22AF4D/nati-chil-meas-prog-eng-2017-2018-rep.pdf. Accessed: 22 Jan 2019.
5. Butland B, Jebb S, Kopelman P, et al. Tackling obesities: future choices-project report 2nd Edition. Government Office for Science. 2007. Available from: https://assets.publishing.service.gov.uk/government/uploads/system/uploads/attachment_data/file/287937/07-1184x-tackling-obesities-future-choices-report.pdf. Accessed: 25 Oct 2018.
6. Kipping RR, Payne C, Lawlor DA. Randomised controlled trial adapting US school obesity prevention to England. *Arch Dis Child* 2008;93(6):469-73 doi:10.1136/adc.2007.116970.
7. Public Health England. Health matters: obesity and the food environment. 2017. Available from: https://www.gov.uk/government/publications/health-matters-obesity-and-the-food-environment/health-matters-obesity-and-the-food-environment--2. Accessed: 12 Nov 2018.
8. Huang TT, Drewnosksi A, Kumanyika S, et al. A systems-oriented multilevel framework for addressing obesity in the 21st century. *Prev Chronic Dis* 2009;6(3):A82.

For peer review only - http://bmjopen.bmj.com/site/about/guidelines.xhtml

9. Rutter H. The single most important intervention to tackle obesity…. *Int J Public Health* 2012;57(4):657-8 doi:10.1007/s00038-012-0385-6.

10. Johnston L, Matteson C, Finegood D. Systems science and obesity policy: a novel framework for analyzing and rethinking population-level planning. *Am J Public Health* 2014;104(7):1270-8 doi:10.2105/AJPH.2014.301884.

11. Brown T, Moore THM, Hooper L, et al. Interventions for preventing obesity in children. *Cochrane Database Syst Rev* 2019, Issue 7. Art No.:CD001871 doi:10.1002/14651858.CD001871.pub4.

12. Dobbins M, DeCorby K, Robeson P, et al. School-based physical activity programs for promoting physical activity and fitness in children and adolescents aged 6 – 18. *Cochrane Database Syst Rev* 2013, Issue 2. Art No.: CD007651 doi:10.1002/14651858.CD007651.pub2.

13. Langford R, Bonell C, Jones H, et al. The WHO health promoting school framework for improving the health and well-being of students and their academic achievement. *Cochrane Database Syst Rev* 2014, Issue 4. Art No.: CD008958 doi:10.1002/14651858.CD008958.pub2.

14. Drummy C, Murtagh EM, McKee DP, et al. The effect of a classroom activity break on physical activity levels and adiposity in primary school children. *J Paediatr Child Health* 2016;52(7):745–9 doi:10.1111/jpc.13182.

15. Wilkie HJ, Standage M, Gillison FB, et al. Correlates of intensity-specific physical activity in children aged 9-11 years: a multilevel analysis of UK data from the international study of childhood obesity, lifestyle and the environment. *BMJ Open* 2018;8(2):e018373–e018373 doi:10.1136/bmjopen-2017-018373.

16. Love R, Adams J, van Sluijs EM. Are school-based physical activity interventions effective and equitable? A meta-analysis of cluster randomised controlled trials with accelerometer-assessed activity. *Obes Rev* 2019;20(6):859-70 doi:10.1111/obr.12823.

For peer review only

17. Micha R, Karageorgou D, Bakogianni I, et al. Effectiveness of school food environment policies on children's dietary behaviors: a systematic review and meta-analysis. *PLoS One* 2018;13(3):e0194555–e0194555 doi:10.1371/journal.pone.0194555.
18. Harrison F, Bentham G, Jones AP, et al. School level correlates with adiposity in 9-10 year old children. *Health Place* 2011;17(3):710-6 doi:10.1016/j.healthplace.2011.01.009.
19. Pallan MJ, Adab P, Sitch AJ, et al. Are school physical activity characteristics associated with weight status in primary school children? A multilevel cross-sectional analysis of routine surveillance data. *Arch Dis Child* 2014;99(2):135-41 doi:10.1136/archdischild-2013-303987.
20. Adab P, Pallan MJ, Lancashire ER, et al. Effectiveness of a childhood obesity prevention programme delivered through schools, targeting 6 and 7 year olds: cluster randomised controlled trial (WAVES study). *BMJ* 2018;360:k211 doi:10.1136/bmj.k211
21. Department for Communities and Local Government. The English indices of deprivation 2010. 2011. Available from: https://assets.publishing.service.gov.uk/government/uploads/system/uploads/attachment_data/file/6871/1871208.pdf. Accessed: 22 Jan 2019.
22. Cole TJ, Freeman JV, Preece MA. Body mass index reference curves for the UK, 1990. *Arch Dis Child* 1995;73(1):25–9 doi:10.1136/adc.73.1.25
23. World Health Organization. Physical status: the use and interpretation of anthropometry [WHO technical report series 854]. Geneva 1995. Available from: https://www.who.int/childgrowth/publications/physical_status/en/. Accessed: 8 Feb 2019.
24. Cade JE, Frear L, Greenwood DC. Assessment of diet in young children with an emphasis on fruit and vegetable intake: using CADET – Child and Diet Evaluation Tool. *Public Health Nutr* 2006; 9(4):501–8 doi: 10.1079/phn2005871.
25. Englyst HN, Cummings JH. Improved method for measurement of dietary fiber as non-starch polysaccharides in plant foods. *J Assoc Off Anal Chem* 1988; 71(4):808—814.

For peer review only - http://bmjopen.bmj.com/site/about/guidelines.xhtml

26. The requirements of school food standards 2014 (SI 2014/1603). 2014. Available from: http://www.legislation.gov.uk/uksi/2014/1603/contents/made. Accessed: 22 Jan 2019.

27. Department of Health and Department for Children, Schools and Families. Introduction to the national healthy schools programme. Available from: http://dera.ioe.ac.uk/6798/7/Introduction_Redacted.pdf. Accessed: 27 Oct 2018.

28. Evans C EL, Mandl V, Christian MS, et al. Impact of school lunch type on nutritional quality of English children's diets. *Public Health Nutr* 2016;19(1):36–45 doi:10.1017/S1368980015000853.

29. Department for Education and Skills. Learning through PE and sport. Available from: https://webarchive.nationalarchives.gov.uk/20130402163246/https:/www.education.gov.uk/publications/eOrderingDownload/LTPES.pdf.pdf. Accessed 10 Oct 2020.

30. Stata Corp. Stata statistical software: release 15. College station, TX: Stata Corp LLC; 2017.

31. Rose G. Sick individuals and sick populations. *Int J Epidemiol* 1985;14(1):32–8 doi: 10.1093/ije/14.1.32.

32. Conolly A. Health survey for England 2015 children's body mass index, overweight and obesity. NHS Digital [ISBN: 978-1-78386-896-4]. 2016. Available from: http://healthsurvey.hscic.gov.uk/media/37776/hse2015-child-obe.pdf. Accessed: 22 Jan 2019.

33. Adab P, Barrett T, Bhopal R, et al. The West Midlands ActiVe lifestyle and healthy Eating in School children (WAVES) study: a cluster randomised controlled trial testing the clinical effectiveness and cost-effectiveness of a multifaceted obesity prevention intervention programme targeted at children aged 6–7 years. Southampton (UK): NIHR Journals Library; 2018 Feb. (Health Technology Assessment, No. 22.8.) doi:10.3310/hta22080.

34. Wolf EJ, Harrington KM, Clark SL, et al. Sample size requirements for structural equation models: an evaluation of power, bias, and solution propriety. *Educ Psychol Meas* 2013;73:913-34 doi: 10.1177/0013164413495237.

For peer review only - http://bmjopen.bmj.com/site/about/guidelines.xhtml

35. Rothman KJ. No adjustments are needed for multiple comparisons. *Epidemiology* 1990;1(1):43-6.

36. Zaborskis A, Moceviciene R, Iannotti RJ. The influence of chronological period of data collection on differences in reported dietary intake among school-aged children surveyed in 39 countries. *J Nutr Educ Behav* 2014;46(5):359–69 doi:10.1016/j.jneb.2013.11.017.

37. Health committee. Childhood obesity: time for action (HC 882). London: The Stationery Office; 2018.

38. Fairbrother H, Curtis P, Goyder E. Where are the schools? Children, families and food practices. *Health Place* 2016;40:51-7 doi:10.1016/j.healthplace.2016.04.013.

39. Golan M, Weizman A. Familial approach to the treatment of childhood obesity: conceptual model. *J Nutr Educ* 2001;33(2):102–7 doi:10.1016/s1499-4046(06)60173-5.

40. Esposito L, Fisher JO, Mennella JA, et al. Developmental perspectives on nutrition and obesity from gestation to adolescence. *Prev Chronic Dis* 2009;6(3):A94.

41. Passmore S, Jones L. A review of the sustainability and impact of a healthy lifestyles programme in primary schools 2–5 years after the intervention phase. *Health Educ Res* 2019;34(1):72-83 doi:10.1093/her/cyy043.

42. Story M, Nanney MS, Schwartz MB. Schools and obesity prevention: creating school environments and policies to promote healthy eating and physical activity. *Milbank Q* 2009; 87(1):71–100 doi:10.1111/j.1468-0009.2009.00548.x.

43. Martin A, Booth JN, Laird Y, et al. Physical activity, diet and other behavioural intervention for improving cognition and school achievement in children and adolescents with obesity or overweight. *Cochrane Database Syst Rev* 2018, Issue 3. Art. No.: CD009728 doi:10.1002/14651858.CD009728.pub4.

For peer review only

Relationship between primary school healthy eating and physical activity promoting environments and children's dietary intake, physical activity and weight status: a longitudinal study in the West Midlands, United Kingdom

Supplementary file

Table 1: School environment and policy scoring

Description of variable	Score allocation
**Healthy eating score**	
School has a policy regarding HE	0 = No<br>2 = Yes
HE is promoted in school curriculum	0 = No<br>2 = Yes
School cookery club to promote HE	0 = No<br>2 = Yes
Involve parents in the promotion of HE	0 = No<br>2 = Yes
Provide a range of ways to promote HE	Split data into tertiles<br>0 = < 5<br>1 = 5 ≤ x < 7<br>2 = ≥ 7
Drinking water is provided throughout the day	0 = No<br>2 = Yes
Breakfast club is available for students	0 = No<br>2 = Yes
Food available at break deemed as healthy	0 = No†<br>2 = Yes†
Proportion of children choosing to eat school lunches	Split data into tertiles<br>0 = < 50%<br>1 = 50% ≤ x < 60%<br>2 = ≥ 60% of children
Governors support HE in school *	0.33 = Weakly support<br>0.67 = Support<br>1.00 = Strongly support
The Senior Leadership Team support HE in school *	0.33 = Weakly support<br>0.67 = Support<br>1.00 = Strongly support
Staff support HE in school *	0.33 = Weakly support<br>0.67 = Support<br>1.00 = Strongly support
School council support HE in school *	0.33 = Weakly support<br>0.67 = Support<br>1.00 = Strongly support
Pupils support HE in school *	0.33 = Weakly support<br>0.67 = Support<br>1.00 = Strongly support
Parents support HE in school *	0.33 = Weakly support<br>0.67 = Support<br>1.00 = Strongly support
**Physical activity score**	
School has a policy regarding PA	0 = No<br>2 = Yes
School has a PA coordinator	0 = No<br>2 = Yes
Staff are trained regarding PA	0 = No<br>2 = Yes

For peer review only - http://bmjopen.bmj.com/site/about/guidelines.xhtml

Description of variable	Score allocation
School has sport partnerships and community links	0 = No<br>2 = Yes
PA is promoted in the school curriculum	0 = No<br>2 = Yes
Involve parents in the promotion of PA	0 = No<br>2 = Yes
Have a walk to school campaign	0 = No<br>2 = Yes
Provide a range of ways to promote PA	Split data into tertiles<br>0 = < 5<br>1 = 5 ≤ x < 7<br>2 = ≥ 7
PE is taught by a specialist	0 = no<br>2 = yes
Range of activities offered in PE meet Department of Education recommendations	0 = no<br>2 = yes
Schools allocating two or more hours to PE per week	0 = for none of the school<br>1 = for some of the years<br>2 = for the whole school
Offer a variety of PA opportunities, other than PE	Split data into tertiles<br>0 = 0<br>1 = 1<br>2 = ≥ 2
Have a range of play equipment to be used	Split data into tertiles<br>0 = < 5<br>1 = 5<br>2 = ≥ 6
Provide a range of physically active/ sports clubs	Split data into tertiles<br>0 = < 4<br>1 = 4 ≤ x < 6<br>2 = ≥ 6
Governors support PA in school *	0.33 = Weakly support<br>0.67 = Support<br>1.00 = Strongly support
The Senior Leadership Team support PA in school *	0.33 = Weakly support<br>0.67 = Support<br>1.00 = Strongly support
Staff support PA in school *	0.33 = Weakly support<br>0.67 = Support<br>1.00 = Strongly support
School council support PA in school *	0.33 = Weakly support<br>0.67 = Support<br>1.00 = Strongly support
Pupils support PA in school *	0.33 = Weakly support<br>0.67 = Support<br>1.00 = Strongly support
Parents support PA in school *	0.33 = Weakly support<br>0.67 = Support<br>1.00 = Strongly support

* Given a maximum score of 1, to ensure that level of support by respective groups did not account for too great a proportion of overall score.
† No = Any food other than fruit, vegetables, milk and juice. Yes = Fruit, vegetables, milk and juice

For peer review only

Table 2: Multilevel models developed for BMI z-score at the three study time points: all fixed effect covariates are presented

	Baseline		Follow-up one		Follow-up two	
	**B (95% CI)**	**p-value**	**B (95% CI)**	**p-value**	**B (95% CI)**	**p-value**
**Trial arm**	N/A	N/A	0.07 (-0.05, 0.19)	0.24	0.03 (-0.07, 0.14)	0.55
**Baseline zBMI**	N/A	N/A	0.95 (0.93, 0.98)	< 0.001	0.92 (0.89, 0.96)	< 0.001
**Index of Multiple Deprivation score**	0.004 (-0.001, 0.008)	0.09	<0.001 (-0.003, 0.002)	0.74	0.0004 (-0.003, 0.003)	0.80
**Female**	-0.08 (-0.22, 0.05)	0.23	-0.11 (-0.18, -0.05)	0.001	-0.16 (-0.24, -0.07)	< 0.001
**Ethnicity (reference White)**						
**Asian**	-0.27 (-0.44, -0.10)	0.002	0.10 (0.01, 0.20)	0.03	0.16 (0.05, 0.28)	0.01
**African Caribbean**	0.38 (0.11, 0.65)	0.01	0.08 (-0.06, 0.22)	0.29	0.01 (-0.18, 0.19)	0.96
**Other**	-0.02 (-0.23, 0.18)	0.83	0.01 (-0.09, 0.12)	0.78	0.04 (-0.10, 0.17)	0.61
**Age**	0.03 (-0.19, 0.25)	0.76	-0.02 (-0.12, 0.09)	0.73	0.02 (-0.12, 0.16)	0.80
**School environment category (reference = minimal health focus)**						
**PA focus**	-0.06 (-0.25, 0.14)	0.57	-0.06 (-0.22, 0.10)	0.48	-0.17 (-0.31, -0.03)	0.02
**HE focus**	-0.09 (-0.28, 0.09)	0.33	-0.02 (-0.18, 0.15)	0.86	-0.02 (-0.16, 0.13)	0.80
**Complete health focus**	0.04 (-0.14, 0.23)	0.64	0.07 (-0.09, 0.23)	0.41	-0.11 (-0.25. 0.03)	0.14

For peer review only

Table 3: Multilevel models developed for waist circumference z-score at the three study time points: all fixed effect covariates are presented

	Baseline		Follow-up one		Follow-up two	
	**B (95% CI)**	**p-value**	**B (95% CI)**	**p-value**	**B (95% CI)**	**p-value**
**Trial arm**	N/A	N/A	-0.12 (-0.31, 0.07)	0.23	-0.09 (-0.25, 0.07)	0.27
**Baseline zWC**	N/A	N/A	0.90 (0.86, 0.94)	< 0.001	0.85 (0.80, 0.89)	< 0.001
**Index of Multiple Deprivation score**	0.002 (-0.003, 0.007)	0.35	-0.001 (-0.004, 0.003)	0.76	0.001 (-0.003, 0.005)	0.48
**Female**	0.07 (-0.07, 0.21)	0.32	-0.06 (-0.15, 0.03)	0.17	0.11 (0.01, 0.21)	0.04
**Ethnicity (reference White)**						
**Asian**	-0.20 (-0.39, -0.01)	0.04	0.19 (0.06, 0.32)	0.003	0.21 (0.07, 0.36)	< 0.01
**African Caribbean**	0.33 (0.04, 0.62)	0.03	0.02 (-0.18, 0.21)	0.88	0.07 (-0.17, 0.31)	0.57
**Other**	0.004 (-0.21, 0.22)	0.97	0.06 (-0.08, 0.20)	0.41	0.05 (-0.18, 0.21)	0.58
**Age**	0.05 (-0.18, 0.29)	0.65	-0.06 (-0.21, 0.09)	0.44	0.05 (-0.13, 0.22)	0.58
**School environment category (reference = minimal health focus)**						
**PA focus**	-0.10 (-0.36, 0.16)	0.44	0.04 (-0.22, 0.30)	0.75	-0.15 (-0.36, 0.06)	0.16
**HE focus**	-0.24 (-0.48, 0.01)	0.06	0.19 (-0.07, 0.45)	0.14	0.07 (-0.15, 0.29)	0.55
**Complete health focus**	0.10 (-0.14, 0.34)	0.40	-0.01 (-0.26, 0.24)	0.95	-0.18 (-0.38, 0.03)	0.09

For peer review only

Table 4: Multilevel models developed for dietary total energy intake at the three study time points: all fixed effect covariates are presented

	Baseline		Follow-up one		Follow-up two	
	**B (95% CI)**	**p-value**	**B (95% CI)**	**p-value**	**B (95% CI**	**p-value**
**Trial arm**	N/A	N/A	18.5 (-364.3, 401.4)	0.92	71.4 (-431.0, 573.7)	0.78
**Baseline dietary total energy intake (kJ/ 24 hours)**	N/A	N/A	0.02 (0.01, 0.06)	0.19	0.06 (0.02, 0.11)	0.002
**Index of Multiple Deprivation score**	13.7 (-5.1, 32.5)	0.15	2.7 (-7.6, 13.0)	0.61	10.1 (-2.7, 22.8)	0.12
**Female**	-586.9 (-1028.2, -145.6)	0.01	-199.7 (-484.2, 84.8)	0.17	-38.9 (-376.8, 298.9)	0.82
**Ethnicity (reference White)**						
**Asian**	-38.4 (-699.0, 622.3)	0.91	-423.5 (-803.7, -43.2)	0.03	304.4 (-153.3, 762.0)	0.19
**African Caribbean**	439.2 (-518.6, 1397.0)	0.37	97.2 (-520.6, 715.1)	0.76	43.9 (-740.1, 827.9)	0.91
**Other**	319.6 (-382.5, 1021.7)	0.37	-505.4 (-944.4, -66.3)	0.02	-120.8 (-654.6, 412.9)	0.66
**Age**	621.5 (-119.3, 1362.3)	0.10	423.3 (-49.1, 895.6)	0.08	353.6 (-208.3, 915.6)	0.22
**School environment category (reference = minimal health focus)**						
**PA focus**	-903.5 (-2402.7, 595.6)	0.24	433.7 (-70.5, 937.9)	0.09	33.2 (-614.5, 681.0)	0.92
**HE focus**	-847.8 (-2255.8, 560.3)	0.24	-63.7 (-598.0, 470.5)	0.82	260.8 (-443.1, 964.7)	0.47
**Complete health focus**	-399.3 (-1801.3, 1002.6)	0.58	32.8 (-464.7, 530.3)	0.90	171.3 (-466.4, 809.1)	0.60

For peer review only

Table 5: Multilevel models developed for fruit and vegetable intake at the three study time points: all fixed effect covariates are presented

	Baseline		Follow-up one		Follow-up two	
	**B (95% CI)**	**p-value**	**B (95% CI)**	**p-value**	**B (95% CI)**	**p-value**
**Trial arm**	N/A	N/A	9.6 (-20.5, 39.6)	0.53	-5.5 (-50.3, 39.2)	0.81
**Baseline fruit and vegetable intake (g/ 24 hours)**	N/A	N/A	0.17 (0.12, 0.22)	< 0.001	0.08 (0.02, 0.15)	0.01
**Index of Multiple Deprivation score**	0.31 (-0.72, 1.4)	0.55	-0.58 (-1.4, 0.20)	0.15	-0.66 (-1.7, 0.43)	0.24
**Female**	11.8 (-13.0, 36.7)	0.35	36.9 (15.7, 58.1)	0.001	12.4 (-15.5, 40.4)	0.38
**Ethnicity (reference White)**						
**Asian**	17.0 (-19.9, 53.8)	0.37	9.6 (-19.1, 38.2)	0.51	46.5 (7.9, 85.0)	0.02
**African Caribbean**	10.8 (-43.0, 64.6)	0.69	64.8 (18.6, 111.0)	0.01	33.2 (-32.1, 98.4)	0.32
**Other**	41.6 (2.2, 81.0)	0.04	26.7 (-6.2, 59.6)	0.11	60.6 (16.1, 105.1)	0.01
**Age**	38.4 (-3.4, 80.1)	0.07	31.5 (-3.7, 66.7)	0.08	16.2 (-30.4, 62.7)	0.50
**School environment category (reference = minimal health focus)**						
**PA focus**	-57.8 (-135.5, 19.8)	0.14	11.8 (-27.9, 51.5)	0.56	-16.0 (-73.9, 41.9)	0.59
**HE focus**	-31.6 (-104.7, 41.4)	0.40	3.0 (-38.6, 44.7)	0.89	-4.4 (-66.9, 58.0)	0.89
**Complete health focus**	-7.3 (-80.0, 65.4)	0.84	27.1 (-11.9, 66.0)	0.17	8.6 (-48.1, 65.3)	0.77

For peer review only

Table 6: Multilevel models developed for PAEE at the three study time points: all fixed effect covariates are presented

	Baseline		Follow-up one		Follow-up two	
	**B (95% CI)**	**p-value**	**B (95% CI)**	**p-value**	**B (95% CI)**	**p-value**
**Trial arm**	N/A	N/A	0.25 (-4.6, 5.1)	0.92	-1.7 (-6.0, 2.6)	0.44
**Baseline PAEE (kJ/kg body weight/ 24 hours)**	N/A	N/A	0.53 (0.45, 0.60)	< 0.001	0.43 (0.36, 0.51)	< 0.001
**Index of Multiple Deprivation score**	0.09 (0.01, 0.19)	0.09	-0.10 (-0.22, 0.02)	0.09	-0.02 (-0.13, 0.10)	0.79
**Female**	-14.1 (-16.9, -11.2)	< 0.001	-4.9 (-8.2, -1.6)	0.004	-7.0 (-10.4, -3.5)	< 0.001
**Ethnicity (reference White)**						
**Asian**	-3.8 (-7.7, 0.13)	0.06	-3.7 (-8.0, 0.62)	0.09	-7.9 (-12.1, -3.6)	< 0.001
**African Caribbean**	-17.8 (-23.8, -11.8)	< 0.001	-6.9 (-14.0, 0.18)	0.06	-12.8 (-21.0, -4.6)	0.002
**Other**	-4.1 (-8.4, 0.24)	0.06	-2.4 (-7.0, 2.3)	0.32	-4.4 (-9.4, 0.73)	0.09
**Age**	0.57 (-4.2, 5.3)	0.81	2.0 (-3.2, 7.3)	0.46	-6.6 (-12.1, -1.1)	0.02
**School environment category (reference = minimal health focus)**						
**PA focus**	-6.0 (-11.7, -0.28)	0.04	-0.79 (-7.2, 5.7)	0.81	1.9 (-3.8, 7.6)	0.51
**HE focus**	-6.3 (-11.8, -0.86)	0.02	3.8 (-3.1, 10.7)	0.28	3.5 (-2.7, 9.7)	0.27
**Complete health focus**	-6.3 (-11.6, -1.0)	0.02	-2.6 (-8.8, 3.6)	0.42	5.3 (0.04, 10.6)	0.05

For peer review only

Table 7: Multilevel models developed for estimated time spent in MVPA at the three study time points: all fixed effect covariates are presented

	Baseline		Follow-up one		Follow-up two	
	**B (95% CI)**	**p-value**	**B (95% CI)**	**p-value**	**B (95% CI)**	**p-value**
**Trial arm**	N/A	N/A	0.57 (-13.5, 14.6)	0.94	3.8 (-7.1, 14.7)	0.49
**Baseline estimated time spent in MVPA (min/ 24 hours)**	N/A	N/A	0.21 (0.11, 0.31)	< 0.001	0.13 (0.06, 0.20)	< 0.001
**Index of Multiple Deprivation score**	0.26 (0.03, 0.50)	0.03	-0.18 (-0.52, 0.15)	0.28	0.04 (-0.29, 0.20)	0.72
**Female**	-13.6 (-19.6, -7.6)	< 0.001	-13.6 (-22.4, -4.7)	0.003	-13.6 (-19.6, -7.6)	< 0.001
**Ethnicity (reference White)**						
**Asian**	-3.0 (-11.6, 5.5)	0.49	-5.8 (-18.0, 6.4)	0.35	-12.5 (-20.8, -4.2)	0.003
**African Caribbean**	2.6 (-10.2, 15.5)	0.69	-3.1 (-23.1, 17.0)	0.77	-7.3 (-22.3, 7.6)	0.34
**Other**	5.8 (-3.4, 15.1)	0.22	-3.6 (-16.8, 9.6)	0.59	-3.4 (-13.0, 6.1)	0.48
**Age**	-0.62 (-10.7, 9.4)	0.90	8.4 (-6.4, 23.2)	0.27	-9.1 (-19.1, 1.0)	0.08
**School environment category (reference = minimal health focus)**						
**PA focus**	-4.2 (-18.5, 10.1)	0.57	-3.7 (-22.7, 15.2)	0.70	3.8 (-10.5, 18.2)	0.60
**HE focus**	-13.2 (-26.8, 0.47)	0.06	-0.80 (-20.9, 19.3)	0.94	0.78 (-14.5, 16.1)	0.92
**Complete health focus**	-14.1 (-27.4, -0.75)	0.04	-6.4 (-24.7, 11.8)	0.49	7.2 (-6.4, 20.9)	0.30

For peer review only - http://bmjopen.bmj.com/site/about/guidelines.xhtml

Table 8: Repeated measures models for outcomes of BMI z-score, waist circumference z-score, dietary total energy intake, fruit and vegetable intake, PAEE and time spent in MVPA: all fixed covariates are presented

	Outcome											
	zBMI		zWC		Dietary total energy intake (kJ/24 hrs)		Fruit and vegetable intake (g/24 hrs)		PAEE (kJ/kg body weight/24 hrs)		Estimated time in MVPA (min/24 hrs)	
	B (95% CI)	p-value	B (95% CI)	p-value	B (95% CI)	p-value	B (95% CI)	p-value	B (95% CI)	p-value	B (95% CI)	p-value
**Trial arm**	-0.04 (-0.19, 0.10)	0.55	-0.11 (-0.28, 0.05)	0.17	322.7 (-120.8, 766.2)	0.15	20.1 (-11.4, 51.6)	0.21	-1.9 (-5.1, 1.4)	0.26	0.27 (-7.5, 8.1)	0.95
**Time point (reference baseline)**												
**Follow-up 1**	0.11 (0.08, 0.15)	<0.001	0.25 (0.20, 0.29)	<0.001	-229.2 (-491.6, 33.2)	0.09	-52.5 (-68.4, -36.6)	<0.001	-2.7 (-4.4, -1.0)	0.001	10.4 (6.0, 14.8)	<0.001
**Follow-up 2**	0.19 (0.15, 0.22)	<0.001	0.30 (0.25, 0.35)	<0.001	445.1 (174.9, 715.2)	0.001	-29.5 (-45.9, -13.1)	<0.001	-15.1 (-16.9, -13.3)	<0.001	-16.5 (-21.1, -11.8)	<0.001
**Index of Multiple Deprivation score**	0.004 (-0.001, 0.01)	0.12	0.003 (-0.002, 0.01)	0.26	10.6 (1.2, 19.9)	0.02	0.05 (-0.62, 0.71)	0.89	0.0002 (-0.8, 0.8)	0.99	0.04 (-0.14, 0.21)	0.67
**Female**	-0.15 (-0.29, -0.01)	0.03	0.06 (-0.07, 0.20)	0.36	-316.1 (-554.0, -87.1)	0.01	20.3 (4.1, 36.5)	0.01	-13.3 (-15.6, -11.0)	<0.001	-14.3 (-18.7, -9.9)	<0.001
**Ethnicity (reference White)**												
**Asian**	-0.19 (-0.36, -0.02)	0.03	-0.09 (-0.26, 0.09)	0.34	-18.8 (-349.0, 311.5)	0.91	19.9 (-3.6, 43.3)	0.10	-6.7 (-9.8, -3.5)	<0.001	-8.1 (-14.3, -1.9)	0.01
**African Caribbean**	0.38 (0.11, 0.65)	0.01	0.31 (0.03, 0.59)	0.03	198.2 (-299.7, 696.0)	0.44	26.1 (-8.9, 61.0)	0.14	-16.9 (-21.9, -12.0)	<0.001	0.80 (-8.8, 10.4)	0.87
**Other**	-0.004 (-0.21, 0.20)	0.97	0.02 (-0.19, 0.23)	0.87	-38.9 (-401.4, 323.5)	0.83	41.4 (15.8, 67.1)	0.002	-5.7 (-9.3, -2.2)	0.002	0.52 (-6.3, 7.3)	0.88
**Age**	0.03 (-0.19, 0.25)	0.49	0.11 (-0.12, 0.33)	0.35	529.3 (148.7, 909.9)	0.01	37.9 (11.0, 64.9)	0.01	-0.70 (-4.6, 3.2)	0.73	-1.8 (-9.2, 5.5)	0.62

For peer review only - http://bmjopen.bmj.com/site/about/guidelines.xhtml

	Outcome											
	**zBMI**		**zWC**		**Dietary total energy intake (kJ/24 hrs)**		**Fruit and vegetable intake (g/24 hrs)**		**PAEE (kJ/kg body weight/24 hrs)**		**Estimated time in MVPA (min/24 hrs)**	
	**B (95% CI)**	**p-value**	**B (95% CI)**	**p-value**	**B (95% CI)**	**p-value**	**B (95% CI)**	**p-value**	**B (95% CI)**	**p-value**	**B (95% CI)**	**p-value**
**School environment category (reference = minimal health focus)**												
**PA focus**	-0.10 (-0.29, 0.10)	0.32	-0.11 (-0.32, 0.11)	0.33	-220.9 (-815.1, 373.4)	0.47	-22.6 (-64.9, 19.6)	0.29	-2.8 (-7.2, 1.6)	0.21	-1.4 (-11.9, 9.1)	0.80
**HE focus**	-0.07 (-0.27, 0.13)	0.47	-0.14 (-0.37, 0.08)	0.21	-483.8 (-1087.4, 119.9)	0.12	-21.6 (-64.4, 21.2)	0.32	-0.83 (-5.3, 3.7)	0.72	-5.0 (-15.7, 5.7)	0.36
**Complete health focus**	0.07 (-0.12, 0.26)	0.49	0.07 (-0.14, 0.28)	0.52	-200.0 (-773.9, 373.8)	0.49	5.16 (-35.6, 45.9)	0.80	-3.0 (-7.2, 1.2)	0.16	-6.4 (-16.5, 3.7)	0.21

For peer review only

STROBE Statement—checklist of items that should be included in reports of observational studies

	Item No	Recommendation	Page No
**Title and abstract**	1	(*a*) Indicate the study's design with a commonly used term in the title or the abstract	1-2
		(*b*) Provide in the abstract an informative and balanced summary of what was done and what was found	2-3
**Introduction**			
Background/rationale	2	Explain the scientific background and rationale for the investigation being reported	4-5
Objectives	3	State specific objectives, including any prespecified hypotheses	5
**Methods**			
Study design	4	Present key elements of study design early in the paper	6
Setting	5	Describe the setting, locations, and relevant dates, including periods of recruitment, exposure, follow-up, and data collection	6-7
Participants	6	(*a*) *Cohort study*—Give the eligibility criteria, and the sources and methods of selection of participants. Describe methods of follow-up *Case-control study*—Give the eligibility criteria, and the sources and methods of case ascertainment and control selection. Give the rationale for the choice of cases and controls *Cross-sectional study*—Give the eligibility criteria, and the sources and methods of selection of participants	6-7
		(*b*) *Cohort study*—For matched studies, give matching criteria and number of exposed and unexposed *Case-control study*—For matched studies, give matching criteria and the number of controls per case	NA
Variables	7	Clearly define all outcomes, exposures, predictors, potential confounders, and effect modifiers. Give diagnostic criteria, if applicable	7-8
Data sources/ measurement	8*	For each variable of interest, give sources of data and details of methods of assessment (measurement). Describe comparability of assessment methods if there is more than one group	7-8
Bias	9	Describe any efforts to address potential sources of bias	6-7, 11-12
Study size	10	Explain how the study size was arrived at	6-7
Quantitative variables	11	Explain how quantitative variables were handled in the analyses. If applicable, describe which groupings were chosen and why	7-12
Statistical methods	12	(*a*) Describe all statistical methods, including those used to control for confounding	10-13
		(*b*) Describe any methods used to examine subgroups and interactions	NA
		(*c*) Explain how missing data were addressed	13
		(*d*) *Cohort study*—If applicable, explain how loss to follow-up was addressed *Case-control study*—If applicable, explain how matching of cases and controls was addressed *Cross-sectional study*—If applicable, describe analytical methods taking account of sampling strategy	13, 25
		(*e*) Describe any sensitivity analyses	NA

For peer review only - http://bmjopen.bmj.com/site/about/guidelines.xhtml

Continued on next page

For peer review only

For peer review only - http://bmjopen.bmj.com/site/about/guidelines.xhtml

**Results**			
Participants	13*	(a) Report numbers of individuals at each stage of study—eg numbers potentially eligible, examined for eligibility, confirmed eligible, included in the study, completing follow-up, and analysed	7, 13
		(b) Give reasons for non-participation at each stage	14
		(c) Consider use of a flow diagram	Flow diagram for trial is reported in main trial paper
Descriptive data	14*	(a) Give characteristics of study participants (eg demographic, clinical, social) and information on exposures and potential confounders	13-14
		(b) Indicate number of participants with missing data for each variable of interest	14, 16 (Tables 2 &3)
		(c) *Cohort study*—Summarise follow-up time (eg, average and total amount)	7
Outcome data	15*	*Cohort study*—Report numbers of outcome events or summary measures over time	16 (Table 3)
		Case-control study—Report numbers in each exposure category, or summary measures of exposure	
		Cross-sectional study—Report numbers of outcome events or summary measures	
Main results	16	(*a*) Give unadjusted estimates and, if applicable, confounder-adjusted estimates and their precision (eg, 95% confidence interval). Make clear which confounders were adjusted for and why they were included	20-23
		(*b*) Report category boundaries when continuous variables were categorized	11-12
		(*c*) If relevant, consider translating estimates of relative risk into absolute risk for a meaningful time period	NA
Other analyses	17	Report other analyses done—eg analyses of subgroups and interactions, and sensitivity analyses	NA
**Discussion**			
Key results	18	Summarise key results with reference to study objectives	24
Limitations	19	Discuss limitations of the study, taking into account sources of potential bias or imprecision. Discuss both direction and magnitude of any potential bias	25-27
Interpretation	20	Give a cautious overall interpretation of results considering objectives, limitations, multiplicity of analyses, results from similar studies, and other relevant evidence	29-30
Generalisability	21	Discuss the generalisability (external validity) of the study results	24-25
**Other information**			
Funding	22	Give the source of funding and the role of the funders for the present study and, if applicable, for the original study on which the present article is based	32

*Give information separately for cases and controls in case-control studies and, if applicable, for exposed and unexposed groups in cohort and cross-sectional studies.

For peer review only

Note: An Explanation and Elaboration article discusses each checklist item and gives methodological background and published examples of transparent reporting. The STROBE checklist is best used in conjunction with this article (freely available on the Web sites of PLoS Medicine at http://www.plosmedicine.org/, Annals of Internal Medicine at http://www.annals.org/, and Epidemiology at http://www.epidem.com/). Information on the STROBE Initiative is available at www.strobe-statement.org.

For peer review only

For peer review only - http://bmjopen.bmj.com/site/about/guidelines.xhtml

BMJ Open

Relationship between primary school healthy eating and physical activity promoting environments and children's dietary intake, physical activity and weight status: a longitudinal study in the West Midlands, United Kingdom

Journal:	*BMJ Open*
Manuscript ID	bmjopen-2020-040833.R2
Article Type:	Original research
Date Submitted by the Author:	23-Nov-2020
Complete List of Authors:	Garden, Elizabeth; University of Birmingham, Medical School<br>Pallan, Miranda; University of Birmingham, Institute of Applied Health Research<br>Clarke, Joanne; University of Birmingham, Institute of Applied Health Research<br>Griffin, Tania ; University of Bath,<br>Hurley, Kiya; University of Birmingham, Cancer Research UK Clinical Trials Unit<br>Lancashire, Emma; University of Birmingham, Institute of Applied Health Research<br>Sitch, Alice; University of Birmingham, Institute of Applied Health Research; University Hospitals Birmingham NHS Foundation Trust, NIHR Birmingham Biomedical Research Centre<br>Passmore, Sandra; Services for Education Ltd,<br>Adab, Peymane; University of Birmingham, Institute of Applied Health Research
**Primary Subject Heading**:	Pharmacology and therapeutics
Secondary Subject Heading:	Epidemiology
Keywords:	PUBLIC HEALTH, NUTRITION & DIETETICS, PREVENTIVE MEDICINE

SCHOLARONE™
Manuscripts

I, the Submitting Author has the right to grant and does grant on behalf of all authors of the Work (as defined in the below author licence), an exclusive licence and/or a non-exclusive licence for contributions from authors who are: i) UK Crown employees; ii) where BMJ has agreed a CC-BY licence shall apply, and/or iii) in accordance with the terms applicable for US Federal Government officers or employees acting as part of their official duties; on a worldwide, perpetual, irrevocable, royalty-free basis to BMJ Publishing Group Ltd ("BMJ") its licensees and where the relevant Journal is co-owned by BMJ to the co-owners of the Journal, to publish the Work in this journal and any other BMJ products and to exploit all rights, as set out in our licence.

The Submitting Author accepts and understands that any supply made under these terms is made by BMJ to the Submitting Author unless you are acting as an employee on behalf of your employer or a postgraduate student of an affiliated institution which is paying any applicable article publishing charge ("APC") for Open Access articles. Where the Submitting Author wishes to make the Work available on an Open Access basis (and intends to pay the relevant APC), the terms of reuse of such Open Access shall be governed by a Creative Commons licence – details of these licences and which Creative Commons licence will apply to this Work are set out in our licence referred to above.

Other than as permitted in any relevant BMJ Author's Self Archiving Policies, I confirm this Work has not been accepted for publication elsewhere, is not being considered for publication elsewhere and does not duplicate material already published. I confirm all authors consent to publication of this Work and authorise the granting of this licence.

For peer review only

Relationship between primary school healthy eating and physical activity promoting environments and children's dietary intake, physical activity and weight status: a longitudinal study in the West Midlands, United Kingdom

Elizabeth Mairenn Garden, Miranda Pallan, Joanne Clarke, Tania Griffin, Kiya Hurley, Emma Lancashire, Alice J Sitch, Sandra Passmore, Peymane Adab 4517

Affiliations:

Elizabeth Mairenn Garden: Student, University of Birmingham, Birmingham, UK

Miranda Pallan: Institute of Applied Health Research, University of Birmingham, Birmingham, UK

Joanne Clarke: Institute of Applied Health Research, University of Birmingham, Birmingham, UK

Tania Griffin: Department for Health, University of Bath; Bath, UK

Kiya Hurley: Cancer Clinical Trials Unit, University of Birmingham; Birmingham, UK

Emma Lancashire: Institute of Applied Health Research, University of Birmingham, Birmingham, UK

Alice J Sitch: NIHR Birmingham Biomedical Research Centre, University Hospitals Birmingham NHS Foundation Trust and University of Birmingham, UK & Institute of Applied Health Research, University of Birmingham, UK

Sandra Passmore: Services for Education; Birmingham, UK

Peymane Adab: Institute of Applied Health Research, University of Birmingham, Birmingham, UK

Corresponding author:

Miranda Pallan: Institute of Applied Health Research, Murray Learning Centre, University of Birmingham, Edgbaston, Birmingham, UK. B15 2TT

Email: M.J.Pallan@bham.ac.uk

Word count: 4,517 (excluding title page, abstract, references, figures and tables)

Abstract word count: 270

For peer review only - http://bmjopen.bmj.com/site/about/guidelines.xhtml

ABSTRACT

Objectives: We aimed to examine the association between food and physical activity environments in primary schools and child anthropometric, healthy eating and physical activity measures.

Design: Observational longitudinal study using data from a childhood obesity prevention trial.

Setting: State-primary schools in the West Midlands region, UK.

Participants: 1,392 pupils who participated in the West Midlands ActiVe lifestyle and healthy Eating in School children (WAVES) childhood obesity prevention trial (2011-2015).

Primary and secondary outcome measures: School environment (exposure) was categorised according to questionnaire responses indicating their support for healthy eating and/or physical activity. Child outcome measures, undertaken at three time-points (age 5-to-6, 7-to-8 and 8-to-9 years), included: body mass index z-scores, dietary intake (using a 24-hour food-tick-list) and physical activity (using an Actiheart monitor over five days). Associations between school food and physical activity environment categories and outcomes were explored through multilevel models.

Results: Data were available for 1,304 children (94% of the study sample). At age 8-to-9 years, children in ten schools with healthy eating and physical activity-supportive environments had a higher physical activity energy expenditure than those in 22 schools with less supportive healthy eating/physical activity environments (mean difference=5.3kJ/kg body weight/24 hours, p=0.05). Children in schools with supportive physical activity environments (n=8) had a lower body mass index z-score than those in schools with less supportive healthy eating/physical activity environments (n=22; mean difference=-0.17, p=0.02). School food and physical activity promoting environments were not significantly associated with dietary outcomes.

Conclusions: School environments that support healthy food and physical activity behaviours may positively influence physical activity and childhood obesity.

Trial registration: The WAVES trial was registered on 19/05/2010, registration number: ISRCTN97000586.

For peer review only - http://bmjopen.bmj.com/site/about/guidelines.xhtml

KEY WORDS

Obesity, Child, Primary school, Environment, Diet, Healthy eating, Physical activity

ARTICLE SUMMARY

Strengths and limitations of this study

- The study was conducted with a large ethnically and socio-economically diverse sample of children over a three year period.
- Outcomes were assessed by objective measures and validated instruments, using standardised procedures.
- The school questionnaire and scoring system used to assess school food and physical activity promoting environments may not give a true representation of the extent to which schools promote health.
- Associations between weight, dietary and physical activity outcomes, and four categories of school food/physical activity environment were explored, but this does not fully take into account the large variation in environments seen across schools.
- The Child And Diet Evaluation Tool (CADET) only measured dietary intake over a single day, so may not be an accurate assessment of habitual intake.

For peer review only

BACKGROUND

Childhood obesity is a global public health crisis,[1] affecting countries of all income levels.[2] Since 2005 the overall trend has stabilised,[3] but its prevalence remains high in England, and a substantial increase in overweight and obesity prevalence is seen across the primary school years.[4] Approximately 23% of children age 4-to-5 years are living with overweight and obesity, and this rises to 34% by age 10-to-11 years.[4] Overweight and obesity in childhood is associated with a range of physical, psychological and social challenges,[5] and is likely to persist into adulthood.[6] It also results in significant healthcare costs, estimated to be around £6 billion per year in the UK, and projected to continue to rise in the coming years.[7] Therefore, prevention strategies targeting primary school-aged children are essential to address this significant health and economic burden.

Over the last decade, prevention strategies have shifted from focusing on individuals to systems approaches;[5,8-10] recognising the multiple interconnecting physical, economic, political, social and cultural influences. Within this context, the environments in which children are situated are critical to consider.

Schools provide a physical, social and educational environment for children and have the ability to shape physical activity (PA) and eating behaviours.[5] However, to date, our understanding of how the whole school environment influences these behaviours, and impacts on childhood obesity, is relatively limited. Research has mainly focused on evaluating specific intervention programmes introduced into schools to target weight-related behaviours. A recent Cochrane systematic review of childhood obesity prevention interventions reported that in the primary school-aged population there is some evidence to suggest that combined dietary and PA interventions in schools had a small positive effect on body mass index z-scores (mean difference between intervention and control groups of −0.05, 95% CI −0.10 to −0.01; based on meta-analysis of 20 randomised controlled trials,

For peer review only

For peer review only - http://bmjopen.bmj.com/site/about/guidelines.xhtml

but with low certainty of evidence).[11] However, there was no evidence to suggest that PA or dietary interventions on their own influenced body mass index z-scores.[11]

Although previous trials, using objective outcomes, suggest that school based interventions have the ability to increase PA levels;[12-15] a recent systematic review found no evidence of an effect of activity interventions on increasing whole-day moderate-to-vigorous physical activity (MVPA; standardised mean difference 0.02, 95% CI -0.07 to 0.11).[16]

Two systematic reviews have explored the effectiveness of school food environment interventions and policies on dietary behaviours. These have reported some beneficial effects, such as increased vegetable consumption, but have found no evidence of effect of these interventions on total energy intake.[13,17] However, there was significant heterogeneity of included studies in terms of the differences between schools and educational systems, which makes it challenging to draw general conclusions.

In general, whilst useful for considering specific intervention programmes designed to target diet and/or PA, the existing evidence gives little information on how the combination of different policies, initiatives and activities in schools (which together can be termed ‘the school environment’) promotes PA, healthy eating (HE), and consequently impacts obesity in children. Among the few studies that have examined the relationship between school level characteristics and obesity-related behaviours, the findings have been mixed and concluded that further research is needed.[18,19]

Objectives

In this study, using data collected as part of a large childhood obesity prevention trial,[20] we aimed to explore the association between school environments promoting HE and/or PA, and

For peer review only - http://bmjopen.bmj.com/site/about/guidelines.xhtml

anthropometric, PA and dietary outcomes in UK primary school children. We hypothesised that school environments promoting HE or PA would positively influence the corresponding behavioural, and anthropometric outcomes in children.

METHODS

Design

This longitudinal, observational study uses data obtained from the West Midlands ActiVe lifestyle and healthy Eating in School children (WAVES) trial; a UK-based cluster randomised controlled trial, conducted between 2011 and 2015, that evaluated the clinical and cost-effectiveness of a 12-month obesity-prevention intervention programme delivered in primary schools.[20] The study reported here used school-level data obtained at trial baseline, and participant outcome data obtained at baseline and two subsequent time points.

Participants

All state primary schools in the West Midlands which included Year groups 1 (age 5-to-6 years) to 5 (age 9-to-10 years) and were within a 50km radius of the University of Birmingham, were eligible for inclusion in the WAVES trial. The WAVES sampling strategy sought to over-recruit schools with a high minority ethnic population through use of a weighted random sample, as the original objective of the trial was to evaluate a childhood obesity prevention intervention in an ethnically diverse population. Schools above the 80th percentile for proportion of pupils of South Asian or Black ethnicity had an increased chance of being sampled, with a 3:1 ratio. To ensure representation of a range of school characteristics, the sampling strategy was further balanced to take into account the proportion of children eligible for free school meals, school size and urban/ rural location. With this sampling method we selected 200 schools, which we randomly ordered and sequentially invited to participate in the trial. To achieve the required sample size of 54 schools, we approached 148 schools; 4 did not respond and 90 declined to participate.

For peer review only - http://bmjopen.bmj.com/site/about/guidelines.xhtml

All Year 1 pupils (age 5-to-6 years) in participating schools were eligible to take part (n=2,462 children). Parental consent was obtained for 60% of children (n=1,470), of whom 1,392 were measured at baseline (57% of those eligible). Child demographic data were obtained from parent questionnaires, or if these were not available, from school records. Home postcodes were obtained for children and mapped to Index of Multiple Deprivation 2010 scores,[21] to give an indicator of socio-economic status.

Pupil outcome measures

Participating pupils took part in assessments undertaken by trained researchers following standardised operating procedures. Apart from demographic data, which was only obtained at baseline, all outcome measures were obtained at three time points: baseline (age 5-to-6 years), follow-up-1 (age 7-to-8 years) and follow-up-2 (age 8-to-9 years).

The outcomes of interest in this study were: body mass index z-score (zBMI); waist circumference z-score (zWC) (both z-scores were calculated using UK 1990 reference curves for children to account for age and sex);[22] total energy intake (kJ/24 hours); fruit and vegetable intake (g/24 hours and portions); PA energy expenditure (PAEE; kJ/kg body weight/24 hours); and estimated time spent in MVPA (min/24 hours; defined as time spent above the acceleration threshold of 1.75 m/second).[23] Measurement instruments and processes are summarised in Table 1.

For peer review only

Table 1: Summary of measurements undertaken and their associated outcome variables

Measurement	Instrument	Number of measurements	Method of assessment	Outcome variable
**Weight (to nearest 0.1 kg)**	Tanita bioimpedance monitor (Tanita SC-331S; Tanita, Tokyo, Japan)	Once	Barefoot and in light clothing	**zBMI:** BMI was calculated by dividing weight (kg) by height (m^2). UK 1990 growth reference charts were used to produce an age- and sex-specific zBMI and define weight categories.[22] Overweight is defined as ≥ 85th centile and obese as ≥ 95th centile.[22]
**Height (to nearest 0.1 cm)**	Leicester height measure	Twice (third measure if difference >0.4cm)†	Barefoot and in light clothing	
**Child demographic data: sex, ethnicity. postcode**	Parent questionnaire (or school records if parent questionnaire not completed)	Once	Parent report or school record	
**Waist circumference (to nearest 0.1cm)**	Flexible, non-stretch, cloth tape measure	Twice (third measure if difference >0.4cm)†	Measure at iliac crest	**zWC:** calculated from WC using UK reference curves.[22]
**Dietary intake**	CADET: A validated 115 item 24-hour food tick list completed for 7 distinct time periods.[24] The CADET tool was developed for use in children aged 3-7 years.[24]	Once (24 hours)	Trained researchers recorded all food and drinks consumed by children during 1 school day. On the same day, children were given a home food diary for completion by a parent/ carer. The following day, trained researchers collected home food diaries and reviewed these with the children. When 1 or more uncompleted sections were identified, the researcher tried to complete these through child dietary recall. Data were processed through the CADET nutrient analysis program at the University of Leeds.	The food and nutrient intake data was used to calculate: **dietary total energy intake (kJ),** and **fruit and vegetable intake (g)** based on the Englyst method.[25]

For peer review only - http://bmjopen.bmj.com/site/about/guidelines.xhtml

**Physical activity**	Actiheart (Cambridge Neurotechnology, Papworth, UK)	Once (worn continuously for 5 days, including 1 weekend)	Attached to the child's chest with 2 ECG electrodes in school by a trained researcher, initialised to record in 30-second epochs	Total movement volume was summarised as average acceleration, along with its underlying movement intensity distribution, using the acceleration and heart rate signals. This was used to calculate: **PA energy expenditure** ¶ and **estimated time spent in MVPA.**

† Where 2 values within ≤ 0.4cm, a definitive measurement value was calculated as the average of the 2. For individuals with 3 values recorded, a definitive measurement value was calculated as the average of the closest pair (within ≤ 0.4cm), or average of all 3 reading (if there were no 2 closest readings, but the differences between values were ≤ 0.4cm). When none of the 3 values were within 0.4cm of each other, no definitive measurement value was calculated.

¶ Children with < 24 hours of valid data over the 5-day measurement period were excluded. To ensure representation across the whole 24-hour period, for those with 24 hours of valid data, only those with a distribution of at least 6 hours in each quadrant (morning 3 am – 9 am; noon 9 am – 3 pm; afternoon 3 pm – 9 pm; and midnight 9 pm – 3 am) were included.

WC, waist-circumference. CADET, Child and Diet Evaluation Tool. zBMI, body mass index z-score. BMI, body mass index. zWC, waist circumference z-score. PA, physical activity. MVPA, moderate-to-vigorous-physical -activity

For peer review only

For peer review only - http://bmjopen.bmj.com/site/about/guidelines.xhtml

Assessment of the school environment

Schools participating in the WAVES trial were asked to complete a baseline questionnaire, which enquired about the school's HE and PA environments. The questionnaire was developed to capture the variation in these school environments across the participating schools to assist in interpretation of the main trial findings. Question items were initially developed by the research team and then sent to a health education adviser for review. Questions were refined following feedback from the adviser. The questionnaire was completed by the head teacher or a nominated staff representative and included tick-box questions, followed by open-ended questions to gain further detail regarding policies and practices in the school.

The Health Promoting School framework requirements, government guidelines and prior research were used to develop a scoring system to categorise schools regarding their food/PA environment based on their questionnaire responses. (Supplementary File Table 1).[17,26-29] The scoring system was developed in an iterative way, by the study team, until a consensus was reached on the scoring method and weighting. The use of government publications ensured that the scoring system encompassed key items that have been determined as important for national policy relating to child health at school (for example the provision of 2 hours or more of physical education (PE) per week).[29]

Each question contributed to the final scores. Questions with continuous response variables (such as number of active after school clubs on offer), were categorised into three groups: high; medium; low, using tertile cut-offs with a score allocation of 2;1;0, respectively. Questions with categorical responses were either ordinal or binary (yes/no) questions. Binary questions, such as schools having a healthy eating policy, were scored as 2/0. For the ordinal variables: schools allocating more than two hours to PE was considered of high importance, they were grouped into schools providing this for all years; some years; no years with a score of 2;1;0; while level of support from stakeholders

For peer review only - http://bmjopen.bmj.com/site/about/guidelines.xhtml

(strongly support; support; weakly support) was deemed as less important, so was scored out of 1 (1;0.67;0.33 respectively).

We developed separate HE and PA scores based on a sum of scores from 15 and 20 questions, with the maximum possible score 24 and 34 respectively. The scores for HE and PA were then converted into percentages. The scores ranged from 44-92% for HE and 24-92% for PA. Using the range of scores across all schools, we used tertile cut-offs to split HE and PA scores into three groups. Schools were then categorised according to their environment: i) complete health focus, supporting both HE and PA (top tertile for both HE and PA scores); ii) HE focus (top tertile for HE scores only); iii) PA focus (top tertile for PA scores only); iv) minimal health focus (not in the top tertile for either HE or PA scores).

Data analysis

All analyses were undertaken using STATA Version 15.[30] Descriptive analyses were used to describe pupil characteristics at baseline and both follow-up time points. Categorical variables were summarised as numbers (percentage). Continuous data were summarised as mean (standard deviation) and median (interquartile range (IQR)), as appropriate.

Due to the clustered nature of the data, multilevel linear regression models were used, with school included as a random effect. Primary analysis explored the relationship between the school environment category and pupil outcome measures at the three-study time-points separately. Additionally, a model was fitted using all time-points, allowing for the repeated measures and including a time covariate. Variables included in the model were: baseline age, sex, ethnicity, Index of Multiple Deprivation score and school environment category. Models of follow-up time-points included adjustment for baseline outcome value and the arm of the WAVES trial. The trial arm covariate refers to whether schools were allocated to intervention or control arms in the main WAVES trial. Trial arm was not included in the baseline analyses as participant outcome data at

For peer review only - http://bmjopen.bmj.com/site/about/guidelines.xhtml

this time point were collected before trial arm allocation. Model assumptions were checked using residual plots and the appropriate inclusion of the school random effect was confirmed through likelihood ratio tests. Covariate data were missing for less than 5% of participants, therefore imputation was not performed and a complete case analysis was used.

Patient and public involvement

The WAVES trial research team included a health education advisor (SP) who advised on engagement with schools and school questionnaire design. Input was sought from a head teacher on methods of participant recruitment and participant information materials. The oversight committee for the trial also included a head teacher, who advised on trial conduct throughout.

RESULTS

Of the 54 schools participating in the WAVES trial, 50 (93%) returned the baseline questionnaire. It is these schools and the pupil participants attending them (n=1,304, 94% of the WAVES trial sample) that were included in the current analyses.

Pupil characteristics

Baseline characteristics for pupils are shown in Table 2. The sociodemographic characteristics of the WAVES trial participants excluded from these analyses (due to non-return of school questionnaires) were compared to the study sample and did not differ significantly by age or gender. Those excluded from the analyses were less likely to be of White British ethnicity, and more likely to be of South Asian ethnicity, or in the most deprived Index of Multiple Deprivation score quintile. Anthropometric, dietary and PA characteristics did not differ between those included and those excluded from the analyses.

For peer review only - http://bmjopen.bmj.com/site/about/guidelines.xhtml

Table 2: Summary of pupil demographic information at baseline

	Baseline value for those analysed in this study (n=1304)
**Age in years: mean (SD) ; missing data (n)***	6.3 (0.31); 61
**Gender: n (%)**	
**Boys**	654 (50.2)
**Girls**	650 (49.9)
**Missing data***	0
**Ethnicity: n (%)**	
**White British**	656 (50.8)
**South Asian**	333 (25.8)
**African-Caribbean**	100 (7.8)
**Other**	202 (15.7)
**Missing data* (n)**	13
**Deprivation quintile: n (%)†**	
**1 (most deprived)**	639 (50.0)
**2**	266 (20.8)
**3**	146 (11.4)
**4**	119 (9.3)
**5 (least deprived)**	109 (8.5)
**Missing data* (n)**	25
**Deprivation score: mean (SD); missing data (n)***	33.8 (17.9); 25

* Missing data values are not included in the denominator.

† Index of multiple deprivation scores categorised into 5 groups using the national quintile cut-offs for England.

IQR, interquartile range.

Outcome measures are summarised for all three time-points in Table 3. Missing data at follow-up 1 and 2 are in part due to attrition of participants as the WAVES trial progressed. The main reasons for attrition were pupils moving away from the school or being absent on the day of data collection. The prevalence of overweight/obesity in the study population was 20.8% at baseline, 26.8% at follow-up-1 (15 months) and 31.1% at follow-up-2 (30 months). Prevalence varied across schools from 8–32% at baseline, 9–47% at follow-up-1 and 9–53% at follow-up-2. There was a statistically significant increase between baseline and follow-up-2 for zBMI and zWC ($p<0.001$). As would be expected, total energy intake increased over the study period as the participating children grew and increased their energy requirement. However, a statistically significant decrease in PAEE and estimated time spent in MVPA ($p<0.001$) was also observed during this period. There was no significant difference in fruit and vegetable intake over time ($p=0.20$).

For peer review only - http://bmjopen.bmj.com/site/about/guidelines.xhtml

Table 3: Summary of pupil outcome measures at 3 timepoints

	Baseline (mean (SD) age = 6.3 (0.3) years)	Follow-up 1 (mean (SD) age = 7.7 (0.3) years)	Follow-up 2 (mean (SD) age = 9.0 (0.3) years)
**Anthropometric**			
**Mean (SD) zBMI; missing data***	0.21 (1.2); 64	0.32 (1.3); 201	0.39(1.3); 293
**BMI group category: n (%)†**			
**Underweight (≤2nd centile)**	29 (2.3)	23 (2.1)	23 (2.3)
**Healthy (>2nd and <85th centiles)**	953 (76.9)	784 (71.1)	674 (66.7)
**Overweight (≥85th and <95th centiles)**	109 (8.8)	126 (11.4)	117 (11.6)
**Obese (≥95th centile)**	149 (12.0)	170 (15.4)	197 (19.5)
**Missing data***	64	201	293
**Median (IQR) zWC; missing data***	0.76 (1.2); 167	0.99 (1.3); 275	1.1 (1.3); 416
**Dietary intake**			
**Median (IQR) dietary total energy intake (kJ/ 24 hours); missing data***	6976.9 (5933.9 – 8101.8); 212	7150.3 (6100.0 – 8279.0); 301	7728.8 (6595.0 – 9128.9); 393
**Median (IQR) fruit and vegetable intake (g/ 24 hours); missing data***	241.8 (149.6 – 352.6); 212	207.8 (111.5 – 320.2); 301	219.5 (117.0 – 346.5); 393
**≥ 5 portion fruit and vegetables: n (%)**			
**Yes**	705 (64.6)	502 (50.1)	519 (57.0)
**No**	387 (35.4)	501 (50.0)	392 (43.0)
**Missing data***	212	301	393
**Physical activity**			
**Mean (SD) PAEE (kJ/kg body weight/ 24 hours); missing data***	94.8 (23.8); 341	91.5 (24.5); 521	79.4 (22.4); 680
**Median (IQR) estimated time spent in MVPA (min/ 24 hours); missing data***	58.1 (41.9 – 81.7); 345	62.0 (39.8 – 101.9); 523	43.1 (31.6 – 64.2); 672

* Missing data values are not included in the denominator.

† Based on UK 1990 reference centile curves and applying cut-offs used for population monitoring.

IQR, interquartile range. zBMI, body mass index z-score. BMI, body mass index. zWC, waist circumference z-score. PAEE, physical activity energy expenditure. MVPA, moderate-to-vigorous physical activity.

For peer review only - http://bmjopen.bmj.com/site/about/guidelines.xhtml

School characteristics

All schools had some activities or policies that promoted HE or PA. Overall schools gained higher scores for PA than HE promotion, with the mean normalised score (adjusted for number and type of questions) being 76% (IQR: 66–85%) for PA and 67% (IQR: 51–74%) for HE. 8 (16%) schools were classed as PA focus, 10 (20%) as HE focus, 10 (20%) as complete health focus and 22 (44%) as minimal health focus. The application of the scoring system to schools based on their baseline questionnaire responses, including information on missing data, is shown in Table 4.

Table 4: Summary of the scoring of schools on healthy eating and physical activity based on baseline questionnaire responses

Description of variable	Number of schools (%)		
**Healthy eating score**			
	**No (score=0)**	**Yes (score =2)**	
School has a policy regarding HE*	11 (22.5)	38 (77.6)	
HE is promoted in school curriculum	10 (20.0)	40 (80.0)	
School cookery club to promote HE	29 (58.0)	21 (42.0)	
Involve parents in the promotion of HE	27 (54.0)	23 (46.0)	
Drinking water is provided throughout the day*	0 (0.0)	49 (100.0)	
Breakfast club is available for students	15 (30.0)	35 (70.0)	
Food provided by the school at break deemed as healthy*	32 (65.3)	17 (34.7)	
	**Low (score=0)**	**Medium (score =1)**	**High (score =2)**
Provide a range of ways to promote HE	12 (24.0)	13 (26.0)	25 (50.0)
Proportion of children choosing to eat school lunches†	12 (32.4)	11 (29.7)	14 (37.8)
	**Weakly support (score=0.33)**	**Support (score=0.67)**	**Strongly support (score=1)**
Governors support HE in school	2 (4.0)	31 (62.0)	17 (34.0)
Senior Leadership Team support HE in school*	0 (0.0)	21 (42.9)	28 (57.1)
Staff support HE in school*	0 (0.0)	27 (55.1)	22 (44.9)
School council support HE in school*	2 (4.1)	25 (51.0)	22 (44.9)
Pupils support HE in school*	5 (10.2)	35 (71.4)	9 (18.4)
Parents support HE in school*	14 (28.6)	31 (63.3)	4 (8.2)

For peer review only - http://bmjopen.bmj.com/site/about/guidelines.xhtml

Description of variable	Number of schools (%)		
**Physical activity score**			
	**No (score=0)**		**Yes (score=2)**
School has a policy regarding PA**	3 (6.3)		45 (93.8)
School has a PA co-ordinator***	4 (8.5)		43 (91.5)
Staff are trained regarding PA***	17 (36.2)		30 (63.8)
Has sport partnerships and community links***	11 (23.4)		36 (76.6)
PA is promoted in the school curriculum**	6 (12.5)		42 (87.5)
Involve parents in the promotion of PA**	32 (66.7)		16 (33.3)
School has a walk to school campaign**	13 (27.1)		35 (72.9)
PE is taught by a specialist**	21 (43.8)		27 (56.3)
Range of activities offered in PE meet Department of Education recommendations**	3 (6.3)		45 (93.8)
	**Low (score=0)**	**Medium (score=1)**	**High (score=2)**
Provide a range of ways to promote PA**	7 (14.6)	20 (41.7)	21 (43.8)
Variety of PA opportunities other than PE**	10 (20.8)	15 (31.3)	23 (47.9)
Have a range of play equipment to be used**	12 (25.0)	18 (37.5)	18 (37.5)
Provide a range of sports clubs**	12 (25.0)	11 (22.9)	25 (52.1)
	**For no years (score=0)**	**For some years (score=1)**	**For all years (score=2)**
Schools allocating 2 or more hours to PE per week **	7 (14.6)	2 (4.2)	39 (81.3)
	**Weakly support (score=0.33)**	**Support (score=0.67)**	**Strongly support (score=1)**
Governors support PA in school**	0 (0.0)	28 (58.3)	20 (41.7)
Senior Leadership Team support PA in school**	0 (0.0)	18 (37.5)	30 (62.5)
Staff support PA in school**	0 (0.0)	22 (45.8)	26 (54.2)
School council support PA in school***	0 (0.0)	24 (51.1)	23 (48.9)
Pupils support PA in school**	0 (0.0)	22 (45.8)	26 (54.2)
Parents support PA in school**	4 (8.3)	33 (68.8)	11 (22.9)

* Missing data from 1 school, ** Missing data from 2 schools, *** Missing data from 3 schools, † Missing data from 13 schools.

Missing data values are not included in the denominator used for percentage calculations.

HE, healthy eating. PA, physical activity. PE, Physical Education.

Not all schools reported having HE or PA policies (11 (22%) with no HE policy; 3 (6%) with no PA policy); however, several of these schools were placed in the PA focus, HE focus or complete health focus categories as they scored highly in other aspects of healthy food and PA promotion activity. All schools provided drinking water throughout the school day. Thirty-five (70%) had a

For peer review only - http://bmjopen.bmj.com/site/about/guidelines.xhtml

breakfast club available to students. However, it was difficult to assess how healthy the food provided was from the information available and only 17 schools (35%) provided a healthy break snack. Forty-five schools (94%) offered a range of activities during PE that met the Department of Education recommendations and 39 (81%) allocated two or more hours to PE per week. However, PE was taught by a specialist in only 27 schools (56%). Parents were perceived to support the HE and PA environment less than other stakeholders in the school. In addition, only 23 (46%) and 16 (33%) of schools involved parents in HE and PA promotion, respectively.

Associations between the school food/PA environment category and childhood anthropometric, diet and PA outcomes

For all models (i.e. models at each time-point and repeated measures models for each outcome), the regression coefficients (B) for the school environment categories are presented in Table 5, with the minimal health focus category as the reference. Regression coefficients for all fixed-effect covariates in the models are presented in Supplementary File Tables 2-8. School environment category was not statistically significantly associated with child zBMI at baseline or follow-up-1. At follow-up-2, compared with minimal health focus schools, pupils in PA focus schools had a lower zBMI (B=-0.17, 95% CI -0.31 to -0.03, p=0.02). Although not statistically significant, compared to those in schools with minimal health focus, children in schools with a complete health focus also had a lower zBMI at follow-up-2 (B=-0.11, 95% CI -0.25 to 0.03, p=0.14). At follow-up-2, PA focus and complete health focus schools also had a lower zWC than minimal health focus schools, but this was non-significant (PA focus: B=-0.15, 95% CI -0.36 to 0.06, p=0.16 and complete health focus: B=-0.18, 95% CI -0.38 to 0.03, p=0.09). Compared with minimal health focus schools, PA focus and the HE focus schools had lower zBMI and zWC in the repeated measures models, but these were non-significant.

For peer review only - http://bmjopen.bmj.com/site/about/guidelines.xhtml

Children in schools in HE, PA and complete health focus categories had significantly lower daily PAEE at baseline than those in minimal health focus schools (PA focus: B=-6.0, 95% CI -11.7 to -0.28, p=0.04; HE focus: B=- 6.3, 95% CI -11.8 to – 0.86, p=0.02; and complete health focus: B=-6.3, 95% CI -11.6 to -1.0, p=0.02). They also did less MVPA at baseline, however, this was only significant for schools with a complete health focus (B=-14.1, 95% CI -27.4 to -0.75, p=0.04). By follow-up-2, this trend reversed, with higher levels of both PAEE and MVPA in the HE, PA and complete health focus schools, compared with minimal health focus schools, although these findings were not significant. We found no significant differences in PAEE across school environment categories in the repeated measures model.

School environment category was not significantly associated with total dietary energy intake or fruit and vegetable intake in any of the models.

For peer review only

For peer review only - http://bmjopen.bmj.com/site/about/guidelines.xhtml

Table 5: The association between the school food/physical activity environment category and pupil obesity, dietary and physical activity outcome measures, estimated using multilevel modelling

Outcome variable	School environment category *	Outcome time point							
		Baseline		Follow-up-1		Follow-up-2		Repeated measures model	
		Regression coefficient (95% CI)	p-value	Regression coefficient (95% CI)	p-value	Regression coefficient (95% CI)	p-value	Regression coefficient (95% CI)	p-value
**zBMI**	**PA focus**	-0.06 (-0.25, 0.14)	0.57	-0.06 (-0.22, 0.10)	0.48	**-0.17 (-0.31, -0.03)**	**0.02**	-0.10 (-0.29, 0.10)	0.32
	**HE focus**	-0.09 (-0.28, 0.09)	0.33	-0.02 (-0.18, 0.15)	0.86	-0.02 (-0.16, 0.13)	0.80	-0.07 (-0.27, 0.13)	0.47
	**Complete health focus**	0.04 (-0.14, 0.23)	0.64	0.07 (-0.09, 0.23)	0.41	-0.11 (-0.25, 0.03)	0.14	0.07 (-0.12, 0.26)	0.49
**zWC**	**PA focus**	-0.10 (-0.36, 0.16)	0.44	0.04 (-0.22, 0.30)	0.75	-0.15 (-0.36, 0.06)	0.16	-0.11 (-0.32, 0.11)	0.33
	**HE focus**	-0.24 (-0.48, 0.01)	0.06	0.19 (-0.07, 0.45)	0.14	0.07 (-0.15, 0.29)	0.55	-0.14 (-0.37, 0.08)	0.21
	**Complete health focus**	0.10 (-0.14, 0.34)	0.40	-0.01 (-0.26, 0.24)	0.95	-0.18 (-0.38, 0.03)	0.09	0.07 (-0.14, 0.28)	0.52
**Dietary total energy intake (kJ/24 hours)**	**PA focus**	-903.5 (-2402.7, 595.6)	0.24	433.7 (-70.5, 937.9)	0.09	33.2 (-614.5, 681.0)	0.92	-220.9 (-815.1, 373.4)	0.47
	**HE focus**	-847.8 (-2255.8, 560.3)	0.24	-63.7 (-598.0, 470.5)	0.82	260.8 (-443.1, 964.7)	0.47	-483.8 (-1087.4, 119.9)	0.12
	**Complete health focus**	-399.3 (-1801.3, 1002.6)	0.58	32.8 (-464.7, 530.3)	0.90	171.3 (-466.4, 809.1)	0.60	-200.0 (-773.9, 373.8)	0.49
**Fruit and vegetable intake (g/24 hours)**	**PA focus**	-57.8 (-135.5, 19.8)	0.14	11.8 (-27.9, 51.5)	0.56	-16.0 (-73.9, 41.9)	0.59	-22.6 (-64.9, 19.6)	0.29
	**HE focus**	-31.6 (-104.7, 41.4)	0.40	3.0 (-38.6, 44.7)	0.89	-4.4 (-66.9, 58.0)	0.89	-21.6 (-64.4, 21.2)	0.32
	**Complete health focus**	-7.3 (-80.0, 65.4)	0.84	27.1 (-11.9, 66.0)	0.17	8.6 (-48.1, 65.3)	0.77	5.2 (-35.6, 45.9)	0.80

For peer review only - http://bmjopen.bmj.com/site/about/guidelines.xhtml

Outcome variable	School environment category *	Outcome time point							
		Baseline		Follow-up-1		Follow-up-2		Repeated measures model	
		Regression coefficient (95% CI)	p-value	Regression coefficient (95% CI)	p-value	Regression coefficient (95% CI)	p-value	Regression coefficient (95% CI)	p-value
PAEE (kJ/kg body weight/24 hours)	PA focus	**-6.0 (-11.7, -0.28)**	**0.04**	-0.79 (-7.2, 5.7)	0.81	1.9 (-3.8, 7.6)	0.51	-2.8 (-7.2, 1.6)	0.21
	HE focus	**-6.3 (-11.8, -0.86)**	**0.02**	3.8 (-3.1, 10.7)	0.28	3.5 (-2.7, 9.7)	0.27	-0.83 (-5.3, 3.7)	0.72
	Complete health focus	**-6.3 (-11.6, -1.0)**	**0.02**	-2.6 (-8.8, 3.6)	0.42	**5.3 (0.04, 10.6)**	**0.05**	-3.0 (-7.2, 1.2)	0.16
Estimated time spent in MVPA (min/24 hours)	PA focus	-4.2 (-18.5, 10.1)	0.57	-3.7 (-22.7, 15.2)	0.70	3.8 (-10.5, 18.2)	0.60	-1.4 (-11.9, 9.1)	0.80
	HE focus	-13.2 (-26.8, 0.47)	0.06	-0.80 (-20.9, 19.3)	0.94	0.78 (-14.5, 16.1)	0.92	-5.0 (-15.7, 5.7)	0.36
	Complete health focus	**-14.1 (-27.4, -0.75)**	**0.04**	-6.4 (-24.7, 11.8)	0.49	7.2 (-6.4, 20.9)	0.30	-6.4 (-16.5, 3.7)	0.21

* Minimal health focus schools are the reference category.

All models with outcomes measured at a single time point include school as a random effect and age at baseline, sex, ethnicity, and index of multiple deprivation score as fixed-effect covariates. Models with outcomes at follow-up-1 and follow-up-2 also include the outcome variable at baseline and the WAVES trial arm as fixed-effect covariates.

Repeated measures models include school and pupil as a random effect, and measurement time point, age at baseline, sex, ethnicity, index of multiple deprivation score and WAVES trial arm as fixed-effect covariates.

Results shown in bold are statistically significant.

zBMI, body mass index z-score. zWC, waist circumference z-score. PAEE, physical activity energy expenditure. MVPA, moderate-to-vigorous physical activity. PA, physical activity. HE, healthy eating.

For peer review only - http://bmjopen.bmj.com/site/about/guidelines.xhtml

DISCUSSION

The questionnaire responses indicated that all schools promoted healthy behaviours to some degree, but there was variation in the extent of their efforts. The school scores indicated that schools tended to focus more on promoting PA than HE. This may relate to increased government focus and spending on PA in schools during the study period.[31] Overall, we found little relationship between the school food/PA environment and pupil anthropometric measures, diet or PA levels. Most associations between the school environment category and the outcome measures were non-significant, and those that were significant were small in magnitude. However, by the second follow-up time point (30 months after baseline) there was a statistically significant lower mean zBMI among pupils in schools with a PA focus, compared with those in schools with minimal health focus. Although the effect size is small (mean difference= -0.17; 95% CI -0.31 to -0.03; p= 0.02), if successfully sustained it has the potential to produce public health benefits at a population level.[32] A similar finding was observed for zWC, although this was non-significant. In line with these associations between school environment activity and obesity-related outcomes, we found that at the second follow-up time point PAEE levels were higher in schools with a complete health focus compared with those with minimal health focus. Therefore, the findings may suggest that a healthy school environment could have an effect over time. In this study, we used baseline data collected when the participants had already been at school for a year, so it is possible that if we had baseline data from before school entry, we would have seen a larger association between school environment and obesity and physical activity outcomes.

Strengths

To our knowledge, this is the first UK study to assess the association between primary school food and PA promoting environments and child obesity measures longitudinally. The study involves a large sample, from an ethnically and socio-economically diverse population, over a three-year period. Outcomes were predominantly assessed by objective measures, using validated instruments

For peer review only - http://bmjopen.bmj.com/site/about/guidelines.xhtml

and standardised procedures. We accounted for clustering and individual level confounders by using multilevel models.

Limitations

The sample includes only 50 primary schools that consented to be part of an obesity prevention trial and parental consent was only obtained for 60% of eligible children within these schools. Our study sample may therefore represent schools with more interest in health promotion and pupils who were healthier. However, obesity prevalence in the participating schools was compared with the prevalence estimated from the National Child Measurement Programme at the time and was found to be similar.[33] Comparisons of demographic characteristics of children with and without parental consent to participate in the WAVES trial (undertaken as part of the WAVES trial) also did not show major differences,[34] suggesting that the study sample is broadly representative.
Further limitations relate to participant outcome measurement and attrition. There was attrition between each measurement time point. A higher proportion of participants of African/Caribbean ethnicity and participants from more deprived groups had missing outcome data at the follow up time points, which may partially account for different findings in the follow-up models compared with the baseline models. Limiting analyses to participants with data at all time points would potentially help us explore this, however, we have not conducted these analyses as they would only involve a small, unrepresentative sample.

The questionnaire used to assess the school environment was developed by the WAVES trial research team with input from a health education adviser to capture variation in the environments of the participating schools. It was assessed for face validity, but not further validated. Schools completed the questionnaire with a varying degree of completeness; therefore, the resulting information may not fully reflect their activity relating to the promotion of healthy eating and PA. The methods for scoring schools in relation to HE and PA were developed by the authors, based on

For peer review only - http://bmjopen.bmj.com/site/about/guidelines.xhtml

past and present guidelines relating to health promoting schools. However, the construct validity of these could not be meaningfully explored through confirmatory factor analysis due to the small number of schools in our sample.[35] Furthermore, the categorisation process of schools was pragmatically determined based on statistical cut-offs, and there may be large variations in health promoting activities within each of the categories. It is possible that, due to limitations of the baseline questionnaire and the lack of validation of the scoring system, the schools' food and PA promoting environments may not have been accurately represented, which in turn may have led to the equivocal findings of the study. In addition, the school environment was only assessed at 1 time point, and it is possible that support for HE or PA in the schools changed over time. Therefore, we have not been able to assess longitudinal changes in the school environment and how these are associated with obesity, diet and PA outcomes.

We explored associations between the school environment and multiple outcomes in this study. It is therefore possible that the statistically significant associations that we found could be due to multiple testing. However, as this was an exploratory, hypothesis generating study, we did not apply a correction for multiple testing as these tend to be conservative and may have led us to miss potential differences between groups that warrant further research.[36] The Child And Diet Evaluation Tool (CADET) was quick and practical with a relatively low response burden,[37] but it only measured dietary intake over a single day, so may not be an accurate assessment of habitual intake.

Finally, the data used in this study were drawn from a randomised controlled trial evaluating a childhood obesity prevention intervention and included data from both intervention and control arms. Although there was no evidence of effectiveness of the intervention from the main trial,[20] we adjusted for the intervention/control status of participants in our analyses. However, even with these adjustments we cannot rule out the possibility of marginal intervention effects influencing the

For peer review only - http://bmjopen.bmj.com/site/about/guidelines.xhtml

association between school food and PA environments and anthropometric, physical activity and dietary outcomes.

Comparison with previous research

This study brings new information regarding the potential impact the school HE and PA environment has on children, as previous research has focused on the effectiveness of specific interventions and policies within schools.

Our findings are in line with those from two systematic reviews that found moderate evidence that school PA interventions are able to reduce BMI.[11,13] Our study included a range of school level activities, rather than focusing on specific interventions, and the effect size observed at 30 months was similar to the summary estimate from the trials.[11]

There is a large pool of research indicating PA interventions increase PA levels in children during the school day,[12-15] although overall PA levels may not be increased.[16] We found that children in schools with an increased focus on PA were less active and engaged in less MVPA at baseline compared with minimal health focus schools, but over time the trend was reversed, suggesting that school PA promotion may increase child PA levels over time. Our findings may also indicate that PA promotion in schools only affects PA levels as children get older, when their PA levels generally decrease.

Previous systematic reviews have not found promising results regarding the impact of interventions on dietary outcomes, although there is some evidence that they are able to increase fruit and vegetable intake.[13,17] This study did not find a relationship between the school environment and child dietary outcomes. The differences between the results of this study and previous research may be as a result of assessing overall environment, rather than specific targeted interventions. In 2018,

For peer review only - http://bmjopen.bmj.com/site/about/guidelines.xhtml

the House of Commons (UK Parliament) stated that there was no convincing evidence the school healthy food standards had been implemented effectively.[38] The majority (n=32, 65%) of schools in this study provided a break-time snack which was deemed unhealthy, suggesting other school food provision may also be unhealthy. Furthermore, the limitations of the questionnaire in assessing school healthy eating environment and those related to the dietary assessment method may also have contributed to differences in findings.

In terms of healthy food and PA promoting environments, we found that school perception of parental support for HE in schools was not high (low parental support perceived by over a quarter of schools). Children consistently refer to home life being more significant in changing behaviour than schools and families have been shown to be pivotal in ensuring improvements to lifestyle in primary school aged children.[39-41] Our findings suggest that in relation to PA and HE, parent-school relationships need strengthening. Nevertheless, the complexity of promoting a healthy school environment and of including parents in such endeavours also need to be considered.[42]

Implications

Consistent with previous research, this study indicates that supporting PA in primary schools may have beneficial health effects in terms of increasing PA levels and reducing weight status. Although schools often prioritise academic attainment over PE and PA, increasing PE in the curriculum has not been found to have negative academic consequences and may improve school achievement.[43,44] Therefore, schools are recommended to increase their efforts to promote PA.

In this study, school environments were not as supportive of HE as expected, which is in line with UK Parliament's report stating that there was a lack of effective implementation of school food standards legislation.[38] Stronger enforcement of existing school food regulation and further regulatory measures may be needed to ensure HE is fully supported in schools.

For peer review only - http://bmjopen.bmj.com/site/about/guidelines.xhtml

All associations between the school food/PA environment and health/behavioural outcomes were of small magnitude, which suggests it has an important but limited role in child health. Parents are key influencers of children's health,[40,41] but in this study the majority of schools did not involve parents in their health promotion efforts. The development of school food and PA promoting environments may be more effective if schools develop better relationships with parents and involve them in their health promotion efforts.

Finally, the changes in health behaviours that we observed were at follow-up-2, once the children had been exposed to the school environment for three years. This may indicate that future intervention studies should have a longer follow-up periods to evaluate their effectiveness.

Conclusion

We found that over time, a school environment supportive of PA may positively influence zBMI and that schools with a more food and PA promoting environment may contribute to increased PA levels in children over time. More research is needed to further understand the influence of the school environment, but in the meantime, schools should be encouraged to provide an environment that is supportive of PA and HE. However, schools cannot undertake health promotion in isolation. They need to be considered as part of a complex system, which requires intervention at multiple points to produce a sustained reduction in child overweight and obesity.

For peer review only

For peer review only - http://bmjopen.bmj.com/site/about/guidelines.xhtml

ACKNOWLEDGEMENTS

We would like to acknowledge the contribution of all the WAVES study trial investigators: University of Birmingham: Peymane Adab (Professor of Chronic Disease Epidemiology & Public Health and Chief Investigator), Tim Barrett (Leonard Parsons Professor of Paediatrics and Child Health), KK Cheng (Professor of Public Health and Primary Care), Jonathan J Deeks (Professor of Biostatistics), Joan L Duda (Professor of Sport and Exercise Psychology), Emma Frew (Professor in Health Economics), Karla Hemming (Professor of Biostatistics), Miranda J Pallan (Reader in Public Health & Epidemiology), Jayne Parry (Professor of Policy and Public Health); University of Cambridge, Cambridge MRC Epidemiology Unit / Norwegian School of Sport Sciences: Ulf Ekelund (Professor in Physical Activity and Health); University of Leeds: Janet E Cade (Professor of Nutritional Epidemiology and Public Health); Loughborough University: Amanda Daley (Professor of Behavioural Medicine); University of Edinburgh: Raj Bhopal (Emeritus Professor of Public Health); University of Warwick: Paramjit Gill (Professor of General Practice); Birmingham Community Healthcare NHS Trust: Eleanor McGee (Public Health Nutrition Lead); Birmingham Services for Education: Sandra Passmore (Education Advisor)

We thank the children, school staff, and parents who participated in the trial; the study team, including Behnoush Mohammadpoor Ahranjani and Emma Popo who helped in overseeing the study measurements and data collection; the administrative team who facilitated the running of the project; the research staff who undertook the study measurements; and Robert Lancashire who developed the trial database and oversaw data management. We also thank our collaborators at the Institute of Metabolic Science at the Medical Research Council (MRC) Epidemiology Unit (University of Cambridge) for their support and assistance, particularly Soren Brage, Kate Westgate, and Stefanie Hollidge, for training of field staff on use and maintenance of Actihearts, for overseeing data preparation, and for analysis and interpretation of the physical activity data; Timo Lakka (principal investigator of the PANIC study, University of Eastern Finland) for sharing

For peer review only

exercise data in a similarly aged cohort of children to allow translation of observed heart rate into activity energy expenditure; staff who are working, or have previously worked, at the Nutrition Epidemiology Group in Leeds, who have supported the trial team in terms of administration of the CADET food tick list and processing of the data to enable us to undertake dietary intake analysis, in particular Neil Hancock, Cristina Cleghorn, Meagan Christian, Jayne Hutchinson, Holly Rippin, and Catherine Rycroft. We also thank Sayeed Haque for his statistical advice on the analyses for this particular study.

HUMAN SUBJECTS APPROVAL STATEMENT

The WAVES trial was approved by NHS Research Ethics Service Committee West Midlands, The Black Country (NHS REC Number: 10/H1202/69) and sponsored by the University of Birmingham (Sponsor Number: RG_10-183). Parents gave written informed consent for children to participate and children gave verbal assent before measurements were taken. Permission to access the WAVES study data for this study was obtained in October 2018.

TRIAL MONITORING AND DATA MONITORING

The trial steering committee met annually and included: chair/statistician: Kelvin Jordan, Keele University; subject expert: Peter Whincup, St George's, University of London; health economist: Louise Longworth, Brunel University; public representative: John Bennett, PHSE advisor; and Chief Investigator: Peymane Adab, University of Birmingham.

FUNDING

This WAVES trial was funded by the National Institute for Health Research (NIHR) Health Technology Assessment Programme (project reference 06/85/11). The views expressed are those of the authors and not necessarily those of the NIHR or the Department of Health and Social Care.

For peer review only

COMPETING INTERESTS

Miranda Pallan, Tania Griffin, Kiya Hurley, Emma Lancashire, Alice Sitch and Peymane Adab hold grants from the UK National Institute for Health Research, and Peymane Adab is Chair of the National Institute for Health Research Public Health Research Funding Committee. There are no other competing interests to declare.

AUTHOR CONTRIBUTION

Elizabeth Mairenn Garden (first author) designed and undertook the analysis reported in this manuscript, and drafted the manuscript. Miranda Pallan and Peymane Adab designed and oversaw the conduct of the trial from which the data is drawn, advised on the design and analysis of the reported study, and also contributed substantially to the manuscript. Joanne Clarke, Tania Griffin, Kiya Hurley and Emma Lancashire all designed school and pupil data collection tools and undertook data collection. They also substantially contributed to the manuscript. Alice Sitch assisted in developing the analysis plan, conducted parts of the analyses and substantially contributed to the manuscript. Sandra Passmore oversaw the main trial from which the data is drawn, advised on the development of the school data collection tool, and substantially contributed to the manuscript, bringing a school policy perspective. All authors approved the final version of the manuscript.

DATA SHARING STATEMENT

Requests for access to data from the WAVES trial should be addressed to the Principal Investigator at p.adab@bham.ac.uk. All proposals requesting data access will need to specify how it is planned to use the data, and all proposals will need approval of the trial co-investigator team before data release.

For peer review only - http://bmjopen.bmj.com/site/about/guidelines.xhtml

REFERENCES

1. Mead E, Brown T, Rees K, et al. Diet, physical activity and behavioural interventions for the treatment of overweight or obese children from the age of 6 to 11 years. *Cochrane Database Syst Rev* 2017; Issue 6. Art No.:CD012651 doi:10.1002/14651858.CD012651.
2. Sahoo K, Sahoo B, Choudhury AK, et al. Childhood obesity: causes and consequences. *J Family Med Prim Care* 2015;4(2):187–92 doi:10.4103/2249-4863.154628.
3. Rutter H. Overweigh and obesity: where are we and where are we heading?. National Obesity Observatory. 2012. Available from: https://www.england.nhs.uk/wp-content/uploads/2012/05/Obesity-Review-Group-19-April-2012-Background-Slide-Set-1-Prevalence-and-Trends.pdf. Accessed: 10 Oct 2020.
4. Stats Team. National child measurement programme England, 2017/ 2018 School Year. 2018. Available from: https://files.digital.nhs.uk/9F/22AF4D/nati-chil-meas-prog-eng-2017-2018-rep.pdf. Accessed: 22 Jan 2019.
5. Butland B, Jebb S, Kopelman P, et al. Tackling obesities: future choices-project report 2nd Edition. Government Office for Science. 2007. Available from: https://assets.publishing.service.gov.uk/government/uploads/system/uploads/attachment_data/file/287937/07-1184x-tackling-obesities-future-choices-report.pdf. Accessed: 25 Oct 2018.
6. Kipping RR, Payne C, Lawlor DA. Randomised controlled trial adapting US school obesity prevention to England. *Arch Dis Child* 2008;93(6):469-73 doi:10.1136/adc.2007.116970.
7. Public Health England. Health matters: obesity and the food environment. 2017. Available from: https://www.gov.uk/government/publications/health-matters-obesity-and-the-food-environment/health-matters-obesity-and-the-food-environment--2. Accessed: 12 Nov 2018.
8. Huang TT, Drewnosksi A, Kumanyika S, et al. A systems-oriented multilevel framework for addressing obesity in the 21st century. *Prev Chronic Dis* 2009;6(3):A82.

For peer review only - http://bmjopen.bmj.com/site/about/guidelines.xhtml

9. Rutter H. The single most important intervention to tackle obesity…. *Int J Public Health* 2012;57(4):657-8 doi:10.1007/s00038-012-0385-6.

10. Johnston L, Matteson C, Finegood D. Systems science and obesity policy: a novel framework for analyzing and rethinking population-level planning. *Am J Public Health* 2014;104(7):1270-8 doi:10.2105/AJPH.2014.301884.

11. Brown T, Moore THM, Hooper L, et al. Interventions for preventing obesity in children. *Cochrane Database Syst Rev* 2019, Issue 7. Art No.:CD001871 doi:10.1002/14651858.CD001871.pub4.

12. Dobbins M, DeCorby K, Robeson P, et al. School-based physical activity programs for promoting physical activity and fitness in children and adolescents aged 6 – 18. *Cochrane Database Syst Rev* 2013, Issue 2. Art No.: CD007651 doi:10.1002/14651858.CD007651.pub2.

13. Langford R, Bonell C, Jones H, et al. The WHO health promoting school framework for improving the health and well-being of students and their academic achievement. *Cochrane Database Syst Rev* 2014, Issue 4. Art No.: CD008958 doi:10.1002/14651858.CD008958.pub2.

14. Drummy C, Murtagh EM, McKee DP, et al. The effect of a classroom activity break on physical activity levels and adiposity in primary school children. *J Paediatr Child Health* 2016;52(7):745–9 doi:10.1111/jpc.13182.

15. Wilkie HJ, Standage M, Gillison FB, et al. Correlates of intensity-specific physical activity in children aged 9-11 years: a multilevel analysis of UK data from the international study of childhood obesity, lifestyle and the environment. *BMJ Open* 2018;8(2):e018373–e018373 doi:10.1136/bmjopen-2017-018373.

16. Love R, Adams J, van Sluijs EM. Are school-based physical activity interventions effective and equitable? A meta-analysis of cluster randomised controlled trials with accelerometer-assessed activity. *Obes Rev* 2019;20(6):859-70 doi:10.1111/obr.12823.

For peer review only - http://bmjopen.bmj.com/site/about/guidelines.xhtml

17. Micha R, Karageorgou D, Bakogianni I, et al. Effectiveness of school food environment policies on children's dietary behaviors: a systematic review and meta-analysis. *PLoS One* 2018;13(3):e0194555–e0194555 doi:10.1371/journal.pone.0194555.

18. Harrison F, Bentham G, Jones AP, et al. School level correlates with adiposity in 9-10 year old children. *Health Place* 2011;17(3):710-6 doi:10.1016/j.healthplace.2011.01.009.

19. Pallan MJ, Adab P, Sitch AJ, et al. Are school physical activity characteristics associated with weight status in primary school children? A multilevel cross-sectional analysis of routine surveillance data. *Arch Dis Child* 2014;99(2):135-41 doi:10.1136/archdischild-2013-303987.

20. Adab P, Pallan MJ, Lancashire ER, et al. Effectiveness of a childhood obesity prevention programme delivered through schools, targeting 6 and 7 year olds: cluster randomised controlled trial (WAVES study). *BMJ* 2018;360:k211 doi:10.1136/bmj.k211

21. Department for Communities and Local Government. The English indices of deprivation 2010. 2011. Available from: https://assets.publishing.service.gov.uk/government/uploads/system/uploads/attachment_data/file/6871/1871208.pdf. Accessed: 22 Jan 2019.

22. Cole TJ, Freeman JV, Preece MA. Body mass index reference curves for the UK, 1990. *Arch Dis Child* 1995;73(1):25–9 doi:10.1136/adc.73.1.25

23. World Health Organization. Physical status: the use and interpretation of anthropometry [WHO technical report series 854]. Geneva 1995. Available from: https://www.who.int/childgrowth/publications/physical_status/en/. Accessed: 8 Feb 2019.

24. Cade JE, Frear L, Greenwood DC. Assessment of diet in young children with an emphasis on fruit and vegetable intake: using CADET – Child and Diet Evaluation Tool. *Public Health Nutr* 2006; 9(4):501–8 doi: 10.1079/phn2005871.

25. Englyst HN, Cummings JH. Improved method for measurement of dietary fiber as non-starch polysaccharides in plant foods. *J Assoc Off Anal Chem* 1988; 71(4):808—814.

For peer review only - http://bmjopen.bmj.com/site/about/guidelines.xhtml

26. The requirements of school food standards 2014 (SI 2014/1603). 2014. Available from: http://www.legislation.gov.uk/uksi/2014/1603/contents/made. Accessed: 22 Jan 2019.

27. Department of Health and Department for Children, Schools and Families. Introduction to the national healthy schools programme. Available from: http://dera.ioe.ac.uk/6798/7/Introduction_Redacted.pdf. Accessed: 27 Oct 2018.

28. Evans C EL, Mandl V, Christian MS, et al. Impact of school lunch type on nutritional quality of English children's diets. *Public Health Nutr* 2016;19(1):36–45 doi:10.1017/S1368980015000853.

29. Department for Education and Skills. Learning through PE and sport. Available from: https://webarchive.nationalarchives.gov.uk/20130402163246/https:/www.education.gov.uk/publications/eOrderingDownload/LTPES.pdf.pdf. Accessed: 10 Oct 2020.

30. Stata Corp. Stata statistical software: release 15. College station, TX: Stata Corp LLC; 2017.

31. The Office for Standards in Education, Children's Services and Skills (Ofsted). The PE and sport premium for primary schools. Available from: https://www.gov.uk/government/publications/the-pe-and-sport-premium-for-primary-schools. Accessed: 20 November 2020.

32. Rose G. Sick individuals and sick populations. *Int J Epidemiol* 1985;14(1):32–8 doi: 10.1093/ije/14.1.32.

33. Conolly A. Health survey for England 2015 children's body mass index, overweight and obesity. NHS Digital [ISBN: 978-1-78386-896-4]. 2016. Available from: http://healthsurvey.hscic.gov.uk/media/37776/hse2015-child-obe.pdf. Accessed: 22 Jan 2019.

34. Adab P, Barrett T, Bhopal R, et al. The West Midlands ActiVe lifestyle and healthy Eating in School children (WAVES) study: a cluster randomised controlled trial testing the clinical effectiveness and cost-effectiveness of a multifaceted obesity prevention intervention

For peer review only

For peer review only - http://bmjopen.bmj.com/site/about/guidelines.xhtml

programme targeted at children aged 6–7 years. Southampton (UK): NIHR Journals Library; 2018 Feb. (Health Technology Assessment, No. 22.8.) doi:10.3310/hta22080.

35. Wolf EJ, Harrington KM, Clark SL, et al. Sample size requirements for structural equation models: an evaluation of power, bias, and solution propriety. *Educ Psychol Meas* 2013;73:913-34 doi: 10.1177/0013164413495237.

36. Rothman KJ. No adjustments are needed for multiple comparisons. *Epidemiology* 1990;1(1):43-6.

37. Zaborskis A, Moceviciene R, Iannotti RJ. The influence of chronological period of data collection on differences in reported dietary intake among school-aged children surveyed in 39 countries. *J Nutr Educ Behav* 2014;46(5):359–69 doi:10.1016/j.jneb.2013.11.017.

38. Health committee. Childhood obesity: time for action (HC 882). London: The Stationery Office; 2018.

39. Fairbrother H, Curtis P, Goyder E. Where are the schools? Children, families and food practices. *Health Place* 2016;40:51-7 doi:10.1016/j.healthplace.2016.04.013.

40. Golan M, Weizman A. Familial approach to the treatment of childhood obesity: conceptual model. *J Nutr Educ* 2001;33(2):102–7 doi:10.1016/s1499-4046(06)60173-5.

41. Esposito L, Fisher JO, Mennella JA, et al. Developmental perspectives on nutrition and obesity from gestation to adolescence. *Prev Chronic Dis* 2009;6(3):A94.

42. Passmore S, Jones L. A review of the sustainability and impact of a healthy lifestyles programme in primary schools 2–5 years after the intervention phase. *Health Educ Res* 2019;34(1):72-83 doi:10.1093/her/cyy043.

43. Story M, Nanney MS, Schwartz MB. Schools and obesity prevention: creating school environments and policies to promote healthy eating and physical activity. *Milbank Q* 2009; 87(1):71–100 doi:10.1111/j.1468-0009.2009.00548.x.

44. Martin A, Booth JN, Laird Y, et al. Physical activity, diet and other behavioural intervention for improving cognition and school achievement in children and adolescents with obesity or

For peer review only - http://bmjopen.bmj.com/site/about/guidelines.xhtml

overweight. *Cochrane Database Syst Rev* 2018, Issue 3. Art. No.: CD009728 doi:10.1002/14651858.CD009728.pub4.

For peer review only

Relationship between primary school healthy eating and physical activity promoting environments and children's dietary intake, physical activity and weight status: a longitudinal study in the West Midlands, United Kingdom

Supplementary file

Table 1: School environment and policy scoring

Description of variable	Score allocation
**Healthy eating score**	
School has a policy regarding HE	0 = No<br>2 = Yes
HE is promoted in school curriculum	0 = No<br>2 = Yes
School cookery club to promote HE	0 = No<br>2 = Yes
Involve parents in the promotion of HE	0 = No<br>2 = Yes
Provide a range of ways to promote HE	Split data into tertiles<br>0 = < 5<br>1 = 5 ≤ x < 7<br>2 = ≥ 7
Drinking water is provided throughout the day	0 = No<br>2 = Yes
Breakfast club is available for students	0 = No<br>2 = Yes
Food available at break deemed as healthy	0 = No†<br>2 = Yes†
Proportion of children choosing to eat school lunches	Split data into tertiles<br>0 = < 50%<br>1 = 50% ≤ x < 60%<br>2 = ≥ 60% of children
Governors support HE in school *	0.33 = Weakly support<br>0.67 = Support<br>1.00 = Strongly support
The Senior Leadership Team support HE in school *	0.33 = Weakly support<br>0.67 = Support<br>1.00 = Strongly support
Staff support HE in school *	0.33 = Weakly support<br>0.67 = Support<br>1.00 = Strongly support
School council support HE in school *	0.33 = Weakly support<br>0.67 = Support<br>1.00 = Strongly support
Pupils support HE in school *	0.33 = Weakly support<br>0.67 = Support<br>1.00 = Strongly support
Parents support HE in school *	0.33 = Weakly support<br>0.67 = Support<br>1.00 = Strongly support
**Physical activity score**	
School has a policy regarding PA	0 = No<br>2 = Yes
School has a PA coordinator	0 = No<br>2 = Yes
Staff are trained regarding PA	0 = No<br>2 = Yes

For peer review only - http://bmjopen.bmj.com/site/about/guidelines.xhtml

**Description of variable**	**Score allocation**
School has sport partnerships and community links	0 = No<br>2 = Yes
PA is promoted in the school curriculum	0 = No<br>2 = Yes
Involve parents in the promotion of PA	0 = No<br>2 = Yes
Have a walk to school campaign	0 = No<br>2 = Yes
Provide a range of ways to promote PA	Split data into tertiles<br>0 = < 5<br>1 = 5 ≤ x < 7<br>2 = ≥ 7
PE is taught by a specialist	0 = no<br>2 = yes
Range of activities offered in PE meet Department of Education recommendations	0 = no<br>2 = yes
Schools allocating two or more hours to PE per week	0 = for none of the school<br>1 = for some of the years<br>2 = for the whole school
Offer a variety of PA opportunities, other than PE	Split data into tertiles<br>0 = 0<br>1 = 1<br>2 = ≥ 2
Have a range of play equipment to be used	Split data into tertiles<br>0 = < 5<br>1 = 5<br>2 = ≥ 6
Provide a range of physically active/ sports clubs	Split data into tertiles<br>0 = < 4<br>1 = 4 ≤ x < 6<br>2 = ≥ 6
Governors support PA in school *	0.33 = Weakly support<br>0.67 = Support<br>1.00 = Strongly support
The Senior Leadership Team support PA in school *	0.33 = Weakly support<br>0.67 = Support<br>1.00 = Strongly support
Staff support PA in school *	0.33 = Weakly support<br>0.67 = Support<br>1.00 = Strongly support
School council support PA in school *	0.33 = Weakly support<br>0.67 = Support<br>1.00 = Strongly support
Pupils support PA in school *	0.33 = Weakly support<br>0.67 = Support<br>1.00 = Strongly support
Parents support PA in school *	0.33 = Weakly support<br>0.67 = Support<br>1.00 = Strongly support

* Given a maximum score of 1, to ensure that level of support by respective groups did not account for too great a proportion of overall score.

† No = Any food other than fruit, vegetables, milk and juice. Yes = Fruit, vegetables, milk and juice

For peer review only

Table 2: Multilevel models developed for BMI z-score at the three study time points: all fixed effect covariates are presented

	Baseline		Follow-up one		Follow-up two	
	**B (95% CI)**	**p-value**	**B (95% CI)**	**p-value**	**B (95% CI)**	**p-value**
**Trial arm**	N/A	N/A	0.07 (-0.05, 0.19)	0.24	0.03 (-0.07, 0.14)	0.55
**Baseline zBMI**	N/A	N/A	0.95 (0.93, 0.98)	< 0.01	0.92 (0.89, 0.96)	< 0.01
**Index of Multiple Deprivation score**	0.004 (-0.001, 0.008)	0.09	<0.001 (-0.003, 0.002)	0.74	0.0004 (-0.003, 0.003)	0.80
**Female (reference male)**	-0.08 (-0.22, 0.05)	0.23	-0.11 (-0.18, -0.05)	< 0.01	-0.16 (-0.24, -0.07)	< 0.01
**Ethnicity (reference White)**						
**Asian**	-0.27 (-0.44, -0.10)	< 0.01	0.10 (0.01, 0.20)	0.03	0.16 (0.05, 0.28)	0.01
**African Caribbean**	0.38 (0.11, 0.65)	0.01	0.08 (-0.06, 0.22)	0.29	0.01 (-0.18, 0.19)	0.96
**Other**	-0.02 (-0.23, 0.18)	0.83	0.01 (-0.09, 0.12)	0.78	0.04 (-0.10, 0.17)	0.61
**Age**	0.03 (-0.19, 0.25)	0.76	-0.02 (-0.12, 0.09)	0.73	0.02 (-0.12, 0.16)	0.80
**School environment category (reference = minimal health focus)**						
**PA focus**	-0.06 (-0.25, 0.14)	0.57	-0.06 (-0.22, 0.10)	0.48	-0.17 (-0.31, -0.03)	0.02
**HE focus**	-0.09 (-0.28, 0.09)	0.33	-0.02 (-0.18, 0.15)	0.86	-0.02 (-0.16, 0.13)	0.80
**Complete health focus**	0.04 (-0.14, 0.23)	0.64	0.07 (-0.09, 0.23)	0.41	-0.11 (-0.25. 0.03)	0.14

For peer review only - http://bmjopen.bmj.com/site/about/guidelines.xhtml

Table 3: Multilevel models developed for waist circumference z-score at the three study time points: all fixed effect covariates are presented

	Baseline		Follow-up one		Follow-up two	
	**B (95% CI)**	**p-value**	**B (95% CI)**	**p-value**	**B (95% CI)**	**p-value**
**Trial arm**	N/A	N/A	-0.12 (-0.31, 0.07)	0.23	-0.09 (-0.25, 0.07)	0.27
**Baseline zWC**	N/A	N/A	0.90 (0.86, 0.94)	< 0.01	0.85 (0.80, 0.89)	< 0.01
**Index of Multiple Deprivation score**	0.002 (-0.003, 0.007)	0.35	-0.001 (-0.004, 0.003)	0.76	0.001 (-0.003, 0.005)	0.48
**Female (reference male)**	0.07 (-0.07, 0.21)	0.32	-0.06 (-0.15, 0.03)	0.17	0.11 (0.01, 0.21)	0.04
**Ethnicity (reference White)**						
**Asian**	-0.20 (-0.39, -0.01)	0.04	0.19 (0.06, 0.32)	< 0.01	0.21 (0.07, 0.36)	< 0.01
**African Caribbean**	0.33 (0.04, 0.62)	0.03	0.02 (-0.18, 0.21)	0.88	0.07 (-0.17, 0.31)	0.57
**Other**	0.004 (-0.21, 0.22)	0.97	0.06 (-0.08, 0.20)	0.41	0.05 (-0.18, 0.21)	0.58
**Age**	0.05 (-0.18, 0.29)	0.65	-0.06 (-0.21, 0.09)	0.44	0.05 (-0.13, 0.22)	0.58
**School environment category (reference = minimal health focus)**						
**PA focus**	-0.10 (-0.36, 0.16)	0.44	0.04 (-0.22, 0.30)	0.75	-0.15 (-0.36, 0.06)	0.16
**HE focus**	-0.24 (-0.48, 0.01)	0.06	0.19 (-0.07, 0.45)	0.14	0.07 (-0.15, 0.29)	0.55
**Complete health focus**	0.10 (-0.14, 0.34)	0.40	-0.01 (-0.26, 0.24)	0.95	-0.18 (-0.38, 0.03)	0.09

For peer review only

Table 4: Multilevel models developed for dietary total energy intake at the three study time points: all fixed effect covariates are presented

	Baseline		Follow-up one		Follow-up two	
	**B (95% CI)**	**p-value**	**B (95% CI)**	**p-value**	**B (95% CI**	**p-value**
**Trial arm**	N/A	N/A	18.5 (-364.3, 401.4)	0.92	71.4 (-431.0, 573.7)	0.78
**Baseline dietary total energy intake (kJ/ 24 hours)**	N/A	N/A	0.02 (0.01, 0.06)	0.19	0.06 (0.02, 0.11)	< 0.01
**Index of Multiple Deprivation score**	13.7 (-5.1, 32.5)	0.15	2.7 (-7.6, 13.0)	0.61	10.1 (-2.7, 22.8)	0.12
**Female (reference male)**	-586.9 (-1028.2, -145.6)	0.01	-199.7 (-484.2, 84.8)	0.17	-38.9 (-376.8, 298.9)	0.82
**Ethnicity (reference White)**						
**Asian**	-38.4 (-699.0, 622.3)	0.91	-423.5 (-803.7, -43.2)	0.03	304.4 (-153.3, 762.0)	0.19
**African Caribbean**	439.2 (-518.6, 1397.0)	0.37	97.2 (-520.6, 715.1)	0.76	43.9 (-740.1, 827.9)	0.91
**Other**	319.6 (-382.5, 1021.7)	0.37	-505.4 (-944.4, -66.3)	0.02	-120.8 (-654.6, 412.9)	0.66
**Age**	621.5 (-119.3, 1362.3)	0.10	423.3 (-49.1, 895.6)	0.08	353.6 (-208.3, 915.6)	0.22
**School environment category (reference = minimal health focus)**						
**PA focus**	-903.5 (-2402.7, 595.6)	0.24	433.7 (-70.5, 937.9)	0.09	33.2 (-614.5, 681.0)	0.92
**HE focus**	-847.8 (-2255.8, 560.3)	0.24	-63.7 (-598.0, 470.5)	0.82	260.8 (-443.1, 964.7)	0.47
**Complete health focus**	-399.3 (-1801.3, 1002.6)	0.58	32.8 (-464.7, 530.3)	0.90	171.3 (-466.4, 809.1)	0.60

For peer review only

Table 5: Multilevel models developed for fruit and vegetable intake at the three study time points: all fixed effect covariates are presented

	Baseline		Follow-up one		Follow-up two	
	B (95% CI)	p-value	B (95% CI)	p-value	B (95% CI)	p-value
**Trial arm**	N/A	N/A	9.6 (-20.5, 39.6)	0.53	-5.5 (-50.3, 39.2)	0.81
**Baseline fruit and vegetable intake (g/ 24 hours)**	N/A	N/A	0.17 (0.12, 0.22)	< 0.01	0.08 (0.02, 0.15)	0.01
**Index of Multiple Deprivation score**	0.31 (-0.72, 1.4)	0.55	-0.58 (-1.4, 0.20)	0.15	-0.66 (-1.7, 0.43)	0.24
**Female (reference male)**	11.8 (-13.0, 36.7)	0.35	36.9 (15.7, 58.1)	< 0.01	12.4 (-15.5, 40.4)	0.38
**Ethnicity (reference White)**						
**Asian**	17.0 (-19.9, 53.8)	0.37	9.6 (-19.1, 38.2)	0.51	46.5 (7.9, 85.0)	0.02
**African Caribbean**	10.8 (-43.0, 64.6)	0.69	64.8 (18.6, 111.0)	0.01	33.2 (-32.1, 98.4)	0.32
**Other**	41.6 (2.2, 81.0)	0.04	26.7 (-6.2, 59.6)	0.11	60.6 (16.1, 105.1)	0.01
**Age**	38.4 (-3.4, 80.1)	0.07	31.5 (-3.7, 66.7)	0.08	16.2 (-30.4, 62.7)	0.50
**School environment category (reference = minimal health focus)**						
**PA focus**	-57.8 (-135.5, 19.8)	0.14	11.8 (-27.9, 51.5)	0.56	-16.0 (-73.9, 41.9)	0.59
**HE focus**	-31.6 (-104.7, 41.4)	0.40	3.0 (-38.6, 44.7)	0.89	-4.4 (-66.9, 58.0)	0.89
**Complete health focus**	-7.3 (-80.0, 65.4)	0.84	27.1 (-11.9, 66.0)	0.17	8.6 (-48.1, 65.3)	0.77

For peer review only

Table 6: Multilevel models developed for PAEE at the three study time points: all fixed effect covariates are presented

	Baseline		Follow-up one		Follow-up two	
	**B (95% CI)**	**p-value**	**B (95% CI)**	**p-value**	**B (95% CI)**	**p-value**
**Trial arm**	N/A	N/A	0.25 (-4.6, 5.1)	0.92	-1.7 (-6.0, 2.6)	0.44
**Baseline PAEE (kJ/kg body weight/ 24 hours)**	N/A	N/A	0.53 (0.45, 0.60)	< 0.01	0.43 (0.36, 0.51)	< 0.01
**Index of Multiple Deprivation score**	0.09 (0.01, 0.19)	0.09	-0.10 (-0.22, 0.02)	0.09	-0.02 (-0.13, 0.10)	0.79
**Female (reference male)**	-14.1 (-16.9, -11.2)	< 0.01	-4.9 (-8.2, -1.6)	< 0.01	-7.0 (-10.4, -3.5)	< 0.01
**Ethnicity (reference White)**						
**Asian**	-3.8 (-7.7, 0.13)	0.06	-3.7 (-8.0, 0.62)	0.09	-7.9 (-12.1, -3.6)	< 0.01
**African Caribbean**	-17.8 (-23.8, -11.8)	< 0.01	-6.9 (-14.0, 0.18)	0.06	-12.8 (-21.0, -4.6)	< 0.01
**Other**	-4.1 (-8.4, 0.24)	0.06	-2.4 (-7.0, 2.3)	0.32	-4.4 (-9.4, 0.73)	0.09
**Age**	0.57 (-4.2, 5.3)	0.81	2.0 (-3.2, 7.3)	0.46	-6.6 (-12.1, -1.1)	0.02
**School environment category (reference = minimal health focus)**						
**PA focus**	-6.0 (-11.7, -0.28)	0.04	-0.79 (-7.2, 5.7)	0.81	1.9 (-3.8, 7.6)	0.51
**HE focus**	-6.3 (-11.8, -0.86)	0.02	3.8 (-3.1, 10.7)	0.28	3.5 (-2.7, 9.7)	0.27
**Complete health focus**	-6.3 (-11.6, -1.0)	0.02	-2.6 (-8.8, 3.6)	0.42	5.3 (0.04, 10.6)	0.05

For peer review only

Table 7: Multilevel models developed for estimated time spent in MVPA at the three study time points: all fixed effect covariates are presented

	Baseline		Follow-up one		Follow-up two	
	**B (95% CI)**	**p-value**	**B (95% CI)**	**p-value**	**B (95% CI)**	**p-value**
**Trial arm**	N/A	N/A	0.57 (-13.5, 14.6)	0.94	3.8 (-7.1, 14.7)	0.49
**Baseline estimated time spent in MVPA (min/ 24 hours)**	N/A	N/A	0.21 (0.11, 0.31)	< 0.01	0.13 (0.06, 0.20)	< 0.01
**Index of Multiple Deprivation score**	0.26 (0.03, 0.50)	0.03	-0.18 (-0.52, 0.15)	0.28	0.04 (-0.29, 0.20)	0.72
**Female (reference male)**	-13.6 (-19.6, -7.6)	< 0.01	-13.6 (-22.4, -4.7)	< 0.01	-13.6 (-19.6, -7.6)	< 0.01
**Ethnicity (reference White)**						
**Asian**	-3.0 (-11.6, 5.5)	0.49	-5.8 (-18.0, 6.4)	0.35	-12.5 (-20.8, -4.2)	< 0.01
**African Caribbean**	2.6 (-10.2, 15.5)	0.69	-3.1 (-23.1, 17.0)	0.77	-7.3 (-22.3, 7.6)	0.34
**Other**	5.8 (-3.4, 15.1)	0.22	-3.6 (-16.8, 9.6)	0.59	-3.4 (-13.0, 6.1)	0.48
**Age**	-0.62 (-10.7, 9.4)	0.90	8.4 (-6.4, 23.2)	0.27	-9.1 (-19.1, 1.0)	0.08
**School environment category (reference = minimal health focus)**						
**PA focus**	-4.2 (-18.5, 10.1)	0.57	-3.7 (-22.7, 15.2)	0.70	3.8 (-10.5, 18.2)	0.60
**HE focus**	-13.2 (-26.8, 0.47)	0.06	-0.80 (-20.9, 19.3)	0.94	0.78 (-14.5, 16.1)	0.92
**Complete health focus**	-14.1 (-27.4, -0.75)	0.04	-6.4 (-24.7, 11.8)	0.49	7.2 (-6.4, 20.9)	0.30

For peer review only - http://bmjopen.bmj.com/site/about/guidelines.xhtml

Table 8: Repeated measures models for outcomes of BMI z-score, waist circumference z-score, dietary total energy intake, fruit and vegetable intake, PAEE and time spent in MVPA: all fixed covariates are presented

	Outcome											
	zBMI		zWC		Dietary total energy intake (kJ/24 hrs)		Fruit and vegetable intake (g/24 hrs)		PAEE (kJ/kg body weight/24 hrs)		Estimated time in MVPA (min/24 hrs)	
	B (95% CI)	p-value	B (95% CI)	p-value	B (95% CI)	p-value	B (95% CI)	p-value	B (95% CI)	p-value	B (95% CI)	p-value
**Trial arm**	-0.04 (-0.19, 0.10)	0.55	-0.11 (-0.28, 0.05)	0.17	322.7 (-120.8, 766.2)	0.15	20.1 (-11.4, 51.6)	0.21	-1.9 (-5.1, 1.4)	0.26	0.27 (-7.5, 8.1)	0.95
**Time point (reference baseline)**												
**Follow-up 1**	0.11 (0.08, 0.15)	<0.01	0.25 (0.20, 0.29)	<0.01	-229.2 (-491.6, 33.2)	0.09	-52.5 (-68.4, -36.6)	<0.01	-2.7 (-4.4, -1.0)	<0.01	10.4 (6.0, 14.8)	<0.01
**Follow-up 2**	0.19 (0.15, 0.22)	<0.01	0.30 (0.25, 0.35)	<0.01	445.1 (174.9, 715.2)	<0.01	-29.5 (-45.9, -13.1)	<0.01	-15.1 (-16.9, -13.3)	<0.01	-16.5 (-21.1, -11.8)	<0.01
**Index of Multiple Deprivation score**	0.004 (-0.001, 0.01)	0.12	0.003 (-0.002, 0.01)	0.26	10.6 (1.2, 19.9)	0.02	0.05 (-0.62, 0.71)	0.89	0.0002 (-0.8, 0.8)	0.99	0.04 (-0.14, 0.21)	0.67
**Female (reference male)**	-0.15 (-0.29, -0.01)	0.03	0.06 (-0.07, 0.20)	0.36	-316.1 (-554.0, -87.1)	0.01	20.3 (4.1, 36.5)	0.01	-13.3 (-15.6, -11.0)	<0.01	-14.3 (-18.7, -9.9)	<0.01
**Ethnicity (reference White)**												
**Asian**	-0.19 (-0.36, -0.02)	0.03	-0.09 (-0.26, 0.09)	0.34	-18.8 (-349.0, 311.5)	0.91	19.9 (-3.6, 43.3)	0.10	-6.7 (-9.8, -3.5)	<0.01	-8.1 (-14.3, -1.9)	0.01
**African Caribbean**	0.38 (0.11, 0.65)	0.01	0.31 (0.03, 0.59)	0.03	198.2 (-299.7, 696.0)	0.44	26.1 (-8.9, 61.0)	0.14	-16.9 (-21.9, -12.0)	<0.01	0.80 (-8.8, 10.4)	0.87
**Other**	-0.004 (-0.21, 0.20)	0.97	0.02 (-0.19, 0.23)	0.87	-38.9 (-401.4, 323.5)	0.83	41.4 (15.8, 67.1)	<0.01	-5.7 (-9.3, -2.2)	<0.01	0.52 (-6.3, 7.3)	0.88
**Age**	0.03 (-0.19, 0.25)	0.49	0.11 (-0.12, 0.33)	0.35	529.3 (148.7, 909.9)	0.01	37.9 (11.0, 64.9)	0.01	-0.70 (-4.6, 3.2)	0.73	-1.8 (-9.2, 5.5)	0.62
**School environment category (reference = minimal health focus)**												

For peer review only - http://bmjopen.bmj.com/site/about/guidelines.xhtml

	Outcome											
	**zBMI**		**zWC**		**Dietary total energy intake (kJ/24 hrs)**		**Fruit and vegetable intake (g/24 hrs)**		**PAEE (kJ/kg body weight/24 hrs)**		**Estimated time in MVPA (min/24 hrs)**	
	**B (95% CI)**	**p-value**	**B (95% CI)**	**p-value**	**B (95% CI)**	**p-value**	**B (95% CI)**	**p-value**	**B (95% CI)**	**p-value**	**B (95% CI)**	**p-value**
**PA focus**	-0.10 (-0.29, 0.10)	0.32	-0.11 (-0.32, 0.11)	0.33	-220.9 (-815.1, 373.4)	0.47	-22.6 (-64.9, 19.6)	0.29	-2.8 (-7.2, 1.6)	0.21	-1.4 (-11.9, 9.1)	0.80
**HE focus**	-0.07 (-0.27, 0.13)	0.47	-0.14 (-0.37, 0.08)	0.21	-483.8 (-1087.4, 119.9)	0.12	-21.6 (-64.4, 21.2)	0.32	-0.83 (-5.3, 3.7)	0.72	-5.0 (-15.7, 5.7)	0.36
**Complete health focus**	0.07 (-0.12, 0.26)	0.49	0.07 (-0.14, 0.28)	0.52	-200.0 (-773.9, 373.8)	0.49	5.16 (-35.6, 45.9)	0.80	-3.0 (-7.2, 1.2)	0.16	-6.4 (-16.5, 3.7)	0.21

For peer review only - http://bmjopen.bmj.com/site/about/guidelines.xhtml

STROBE Statement—checklist of items that should be included in reports of observational studies

	Item No	Recommendation	Page No
**Title and abstract**	1	(*a*) Indicate the study's design with a commonly used term in the title or the abstract	1-2
		(*b*) Provide in the abstract an informative and balanced summary of what was done and what was found	2-3
**Introduction**			
Background/rationale	2	Explain the scientific background and rationale for the investigation being reported	4-5
Objectives	3	State specific objectives, including any prespecified hypotheses	5
**Methods**			
Study design	4	Present key elements of study design early in the paper	6
Setting	5	Describe the setting, locations, and relevant dates, including periods of recruitment, exposure, follow-up, and data collection	6-7
Participants	6	(*a*) *Cohort study*—Give the eligibility criteria, and the sources and methods of selection of participants. Describe methods of follow-up<br>*Case-control study*—Give the eligibility criteria, and the sources and methods of case ascertainment and control selection. Give the rationale for the choice of cases and controls<br>*Cross-sectional study*—Give the eligibility criteria, and the sources and methods of selection of participants	6-7
		(*b*) *Cohort study*—For matched studies, give matching criteria and number of exposed and unexposed<br>*Case-control study*—For matched studies, give matching criteria and the number of controls per case	NA
Variables	7	Clearly define all outcomes, exposures, predictors, potential confounders, and effect modifiers. Give diagnostic criteria, if applicable	7-8
Data sources/ measurement	8*	For each variable of interest, give sources of data and details of methods of assessment (measurement). Describe comparability of assessment methods if there is more than one group	7-8
Bias	9	Describe any efforts to address potential sources of bias	6-7, 11-12
Study size	10	Explain how the study size was arrived at	6-7
Quantitative variables	11	Explain how quantitative variables were handled in the analyses. If applicable, describe which groupings were chosen and why	7-12
Statistical methods	12	(*a*) Describe all statistical methods, including those used to control for confounding	10-13
		(*b*) Describe any methods used to examine subgroups and interactions	NA
		(*c*) Explain how missing data were addressed	13
		(*d*) *Cohort study*—If applicable, explain how loss to follow-up was addressed<br>*Case-control study*—If applicable, explain how matching of cases and controls was addressed<br>*Cross-sectional study*—If applicable, describe analytical methods taking account of sampling strategy	13, 25
		(*e*) Describe any sensitivity analyses	NA

For peer review only - http://bmjopen.bmj.com/site/about/guidelines.xhtml

Continued on next page

For peer review only

For peer review only - http://bmjopen.bmj.com/site/about/guidelines.xhtml

**Results**			
Participants	13*	(a) Report numbers of individuals at each stage of study—eg numbers potentially eligible, examined for eligibility, confirmed eligible, included in the study, completing follow-up, and analysed	7, 13
		(b) Give reasons for non-participation at each stage	14
		(c) Consider use of a flow diagram	Flow diagram for trial is reported in main trial paper
Descriptive data	14*	(a) Give characteristics of study participants (eg demographic, clinical, social) and information on exposures and potential confounders	13-14
		(b) Indicate number of participants with missing data for each variable of interest	14, 16 (Tables 2 &3)
		(c) *Cohort study*—Summarise follow-up time (eg, average and total amount)	7
Outcome data	15*	*Cohort study*—Report numbers of outcome events or summary measures over time	16 (Table 3)
		Case-control study—Report numbers in each exposure category, or summary measures of exposure	
		Cross-sectional study—Report numbers of outcome events or summary measures	
Main results	16	(*a*) Give unadjusted estimates and, if applicable, confounder-adjusted estimates and their precision (eg, 95% confidence interval). Make clear which confounders were adjusted for and why they were included	20-23
		(*b*) Report category boundaries when continuous variables were categorized	11-12
		(*c*) If relevant, consider translating estimates of relative risk into absolute risk for a meaningful time period	NA
Other analyses	17	Report other analyses done—eg analyses of subgroups and interactions, and sensitivity analyses	NA
**Discussion**			
Key results	18	Summarise key results with reference to study objectives	24
Limitations	19	Discuss limitations of the study, taking into account sources of potential bias or imprecision. Discuss both direction and magnitude of any potential bias	25-27
Interpretation	20	Give a cautious overall interpretation of results considering objectives, limitations, multiplicity of analyses, results from similar studies, and other relevant evidence	29-30
Generalisability	21	Discuss the generalisability (external validity) of the study results	24-25
**Other information**			
Funding	22	Give the source of funding and the role of the funders for the present study and, if applicable, for the original study on which the present article is based	32

*Give information separately for cases and controls in case-control studies and, if applicable, for exposed and unexposed groups in cohort and cross-sectional studies.

For peer review only

Note: An Explanation and Elaboration article discusses each checklist item and gives methodological background and published examples of transparent reporting. The STROBE checklist is best used in conjunction with this article (freely available on the Web sites of PLoS Medicine at http://www.plosmedicine.org/, Annals of Internal Medicine at http://www.annals.org/, and Epidemiology at http://www.epidem.com/). Information on the STROBE Initiative is available at www.strobe-statement.org.

For peer review only

For peer review only - http://bmjopen.bmj.com/site/about/guidelines.xhtml

BMJ Open

Relationship between primary school healthy eating and physical activity promoting environments and children's dietary intake, physical activity and weight status: a longitudinal study in the West Midlands, United Kingdom

Journal:	*BMJ Open*
Manuscript ID	bmjopen-2020-040833.R3
Article Type:	Original research
Date Submitted by the Author:	04-Dec-2020

Complete List of Authors:	Garden, Elizabeth; University of Birmingham, Medical School<br>Pallan, Miranda; University of Birmingham, Institute of Applied Health Research<br>Clarke, Joanne; University of Birmingham, Institute of Applied Health Research<br>Griffin, Tania ; University of Bath,<br>Hurley, Kiya; University of Birmingham, Cancer Research UK Clinical Trials Unit<br>Lancashire, Emma; University of Birmingham, Institute of Applied Health Research<br>Sitch, Alice; University of Birmingham, Institute of Applied Health Research; University Hospitals Birmingham NHS Foundation Trust, NIHR Birmingham Biomedical Research Centre<br>Passmore, Sandra; Services for Education Ltd,<br>Adab, Peymane; University of Birmingham, Institute of Applied Health Research

**Primary Subject Heading**:	Pharmacology and therapeutics
Secondary Subject Heading:	Epidemiology
Keywords:	PUBLIC HEALTH, NUTRITION & DIETETICS, PREVENTIVE MEDICINE

SCHOLARONE™
Manuscripts

I, the Submitting Author has the right to grant and does grant on behalf of all authors of the Work (as defined in the below author licence), an exclusive licence and/or a non-exclusive licence for contributions from authors who are: i) UK Crown employees; ii) where BMJ has agreed a CC-BY licence shall apply, and/or iii) in accordance with the terms applicable for US Federal Government officers or employees acting as part of their official duties; on a worldwide, perpetual, irrevocable, royalty-free basis to BMJ Publishing Group Ltd ("BMJ") its licensees and where the relevant Journal is co-owned by BMJ to the co-owners of the Journal, to publish the Work in this journal and any other BMJ products and to exploit all rights, as set out in our licence.

The Submitting Author accepts and understands that any supply made under these terms is made by BMJ to the Submitting Author unless you are acting as an employee on behalf of your employer or a postgraduate student of an affiliated institution which is paying any applicable article publishing charge ("APC") for Open Access articles. Where the Submitting Author wishes to make the Work available on an Open Access basis (and intends to pay the relevant APC), the terms of reuse of such Open Access shall be governed by a Creative Commons licence – details of these licences and which Creative Commons licence will apply to this Work are set out in our licence referred to above.

Other than as permitted in any relevant BMJ Author's Self Archiving Policies, I confirm this Work has not been accepted for publication elsewhere, is not being considered for publication elsewhere and does not duplicate material already published. I confirm all authors consent to publication of this Work and authorise the granting of this licence.

For peer review only

Relationship between primary school healthy eating and physical activity promoting environments and children's dietary intake, physical activity and weight status: a longitudinal study in the West Midlands, United Kingdom

Elizabeth Mairenn Garden, Miranda Pallan, Joanne Clarke, Tania Griffin, Kiya Hurley, Emma Lancashire, Alice J Sitch, Sandra Passmore, Peymane Adab 4517

For peer review only

Affiliations:

Elizabeth Mairenn Garden: Student, University of Birmingham, Birmingham, UK

Miranda Pallan: Institute of Applied Health Research, University of Birmingham, Birmingham, UK

Joanne Clarke: Institute of Applied Health Research, University of Birmingham, Birmingham, UK

Tania Griffin: Department for Health, University of Bath; Bath, UK

Kiya Hurley: Cancer Clinical Trials Unit, University of Birmingham; Birmingham, UK

Emma Lancashire: Institute of Applied Health Research, University of Birmingham, Birmingham, UK

Alice J Sitch: NIHR Birmingham Biomedical Research Centre, University Hospitals Birmingham NHS Foundation Trust and University of Birmingham, UK & Institute of Applied Health Research, University of Birmingham, UK

Sandra Passmore: Services for Education; Birmingham, UK

Peymane Adab: Institute of Applied Health Research, University of Birmingham, Birmingham, UK

Corresponding author:

Miranda Pallan: Institute of Applied Health Research, Murray Learning Centre, University of Birmingham, Edgbaston, Birmingham, UK. B15 2TT

Email: M.J.Pallan@bham.ac.uk

Word count: 4,517 (excluding title page, abstract, references, figures and tables)

Abstract word count: 270

For peer review only - http://bmjopen.bmj.com/site/about/guidelines.xhtml

ABSTRACT

Objectives: We aimed to examine the association between food and physical activity environments in primary schools and child anthropometric, healthy eating and physical activity measures.

Design: Observational longitudinal study using data from a childhood obesity prevention trial.

Setting: State-primary schools in the West Midlands region, UK.

Participants: 1,392 pupils who participated in the West Midlands ActiVe lifestyle and healthy Eating in School children (WAVES) childhood obesity prevention trial (2011-2015).

Primary and secondary outcome measures: School environment (exposure) was categorised according to questionnaire responses indicating their support for healthy eating and/or physical activity. Child outcome measures, undertaken at three time-points (age 5-to-6, 7-to-8 and 8-to-9 years), included: body mass index z-scores, dietary intake (using a 24-hour food-tick-list) and physical activity (using an Actiheart monitor over five days). Associations between school food and physical activity environment categories and outcomes were explored through multilevel models.

Results: Data were available for 1,304 children (94% of the study sample). At age 8-to-9 years, children in ten schools with healthy eating and physical activity-supportive environments had a higher physical activity energy expenditure than those in 22 schools with less supportive healthy eating/physical activity environments (mean difference=5.3kJ/kg body weight/24 hours, p=0.05). Children in schools with supportive physical activity environments (n=8) had a lower body mass index z-score than those in schools with less supportive healthy eating/physical activity environments (n=22; mean difference=-0.17, p=0.02). School food and physical activity promoting environments were not significantly associated with dietary outcomes.

Conclusions: School environments that support healthy food and physical activity behaviours may positively influence physical activity and childhood obesity.

Trial registration: The WAVES trial was registered on 19/05/2010, registration number: ISRCTN97000586.

For peer review only

KEY WORDS

Obesity, Child, Primary school, Environment, Diet, Healthy eating, Physical activity

ARTICLE SUMMARY

Strengths and limitations of this study

- The study was conducted with a large ethnically and socio-economically diverse sample of children over a three year period.
- Outcomes were assessed by objective measures and validated instruments, using standardised procedures.
- The school questionnaire and scoring system used to assess school food and physical activity promoting environments may not give a true representation of the extent to which schools promote health.
- Associations between weight, dietary and physical activity outcomes, and four categories of school food/physical activity environment were explored, but this does not fully take into account the large variation in environments seen across schools.
- The Child And Diet Evaluation Tool (CADET) only measured dietary intake over a single day, so may not be an accurate assessment of habitual intake.

For peer review only

BACKGROUND

Childhood obesity is a global public health crisis,[1] affecting countries of all income levels.[2] Since 2005 the overall trend has stabilised,[3] but its prevalence remains high in England, and a substantial increase in overweight and obesity prevalence is seen across the primary school years.[4] Approximately 23% of children age 4-to-5 years are living with overweight and obesity, and this rises to 34% by age 10-to-11 years.[4] Overweight and obesity in childhood is associated with a range of physical, psychological and social challenges,[5] and is likely to persist into adulthood.[6] It also results in significant healthcare costs, estimated to be around £6 billion per year in the UK, and projected to continue to rise in the coming years.[7] Therefore, prevention strategies targeting primary school-aged children are essential to address this significant health and economic burden.

Over the last decade, prevention strategies have shifted from focusing on individuals to systems approaches;[5,8-10] recognising the multiple interconnecting physical, economic, political, social and cultural influences. Within this context, the environments in which children are situated are critical to consider.

Schools provide a physical, social and educational environment for children and have the ability to shape physical activity (PA) and eating behaviours.[5] However, to date, our understanding of how the whole school environment influences these behaviours, and impacts on childhood obesity, is relatively limited. Research has mainly focused on evaluating specific intervention programmes introduced into schools to target weight-related behaviours. A recent Cochrane systematic review of childhood obesity prevention interventions reported that in the primary school-aged population there is some evidence to suggest that combined dietary and PA interventions in schools had a small positive effect on body mass index z-scores (mean difference between intervention and control groups of −0.05, 95% CI −0.10 to −0.01; based on meta-analysis of 20 randomised controlled trials,

For peer review only

but with low certainty of evidence).[11] However, there was no evidence to suggest that PA or dietary interventions on their own influenced body mass index z-scores.[11]

Although previous trials, using objective outcomes, suggest that school based interventions have the ability to increase PA levels;[12-15] a recent systematic review found no evidence of an effect of activity interventions on increasing whole-day moderate-to-vigorous physical activity (MVPA; standardised mean difference 0.02, 95% CI -0.07 to 0.11).[16]

Two systematic reviews have explored the effectiveness of school food environment interventions and policies on dietary behaviours. These have reported some beneficial effects, such as increased vegetable consumption, but have found no evidence of effect of these interventions on total energy intake.[13,17] However, there was significant heterogeneity of included studies in terms of the differences between schools and educational systems, which makes it challenging to draw general conclusions.

In general, whilst useful for considering specific intervention programmes designed to target diet and/or PA, the existing evidence gives little information on how the combination of different policies, initiatives and activities in schools (which together can be termed 'the school environment') promotes PA, healthy eating (HE), and consequently impacts obesity in children. Among the few studies that have examined the relationship between school level characteristics and obesity-related behaviours, the findings have been mixed and concluded that further research is needed.[18,19]

Objectives

In this study, using data collected as part of a large childhood obesity prevention trial,[20] we aimed to explore the association between school environments promoting HE and/or PA, and

For peer review only - http://bmjopen.bmj.com/site/about/guidelines.xhtml

anthropometric, PA and dietary outcomes in UK primary school children. We hypothesised that school environments promoting HE or PA would positively influence the corresponding behavioural, and anthropometric outcomes in children.

METHODS

Design

This longitudinal, observational study uses data obtained from the West Midlands ActiVe lifestyle and healthy Eating in School children (WAVES) trial; a UK-based cluster randomised controlled trial, conducted between 2011 and 2015, that evaluated the clinical and cost-effectiveness of a 12-month obesity-prevention intervention programme delivered in primary schools.[20] The study reported here used school-level data obtained at trial baseline, and participant outcome data obtained at baseline and two subsequent time points.

Participants

All state primary schools in the West Midlands which included Year groups 1 (age 5-to-6 years) to 5 (age 9-to-10 years) and were within a 50km radius of the University of Birmingham, were eligible for inclusion in the WAVES trial. The WAVES sampling strategy sought to over-recruit schools with a high minority ethnic population through use of a weighted random sample, as the original objective of the trial was to evaluate a childhood obesity prevention intervention in an ethnically diverse population. Schools above the 80th percentile for proportion of pupils of South Asian or Black ethnicity had an increased chance of being sampled, with a 3:1 ratio. To ensure representation of a range of school characteristics, the sampling strategy was further balanced to take into account the proportion of children eligible for free school meals, school size and urban/ rural location. With this sampling method we selected 200 schools, which we randomly ordered and sequentially invited to participate in the trial. To achieve the required sample size of 54 schools, we approached 148 schools; 4 did not respond and 90 declined to participate.

For peer review only - http://bmjopen.bmj.com/site/about/guidelines.xhtml

All Year 1 pupils (age 5-to-6 years) in participating schools were eligible to take part (n=2,462 children). Parental consent was obtained for 60% of children (n=1,470), of whom 1,392 were measured at baseline (57% of those eligible). Child demographic data were obtained from parent questionnaires, or if these were not available, from school records. Home postcodes were obtained for children and mapped to Index of Multiple Deprivation 2010 scores,[21] to give an indicator of socio-economic status.

Pupil outcome measures

Participating pupils took part in assessments undertaken by trained researchers following standardised operating procedures. Apart from demographic data, which was only obtained at baseline, all outcome measures were obtained at three time points: baseline (age 5-to-6 years), follow-up-1 (age 7-to-8 years) and follow-up-2 (age 8-to-9 years).

The outcomes of interest in this study were: body mass index z-score (zBMI); waist circumference z-score (zWC) (both z-scores were calculated using UK 1990 reference curves for children to account for age and sex);[22] total energy intake (kJ/24 hours); fruit and vegetable intake (g/24 hours and portions); PA energy expenditure (PAEE; kJ/kg body weight/24 hours); and estimated time spent in MVPA (min/24 hours; defined as time spent above the acceleration threshold of 1.75 m/second).[23] Measurement instruments and processes are summarised in Table 1.

For peer review only

Table 1: Summary of measurements undertaken and their associated outcome variables

Measurement	Instrument	Number of measurements	Method of assessment	Outcome variable
**Weight (to nearest 0.1 kg)**	Tanita bioimpedance monitor (Tanita SC-331S; Tanita, Tokyo, Japan)	Once	Barefoot and in light clothing	**zBMI:** BMI was calculated by dividing weight (kg) by height (m^2). UK 1990 growth reference charts were used to produce an age- and sex-specific zBMI and define weight categories.[22] Overweight is defined as ≥ 85th centile and obese as ≥ 95th centile.[22]
**Height (to nearest 0.1 cm)**	Leicester height measure	Twice (third measure if difference >0.4cm)†	Barefoot and in light clothing	
**Child demographic data: sex, ethnicity. postcode**	Parent questionnaire (or school records if parent questionnaire not completed)	Once	Parent report or school record	
**Waist circumference (to nearest 0.1cm)**	Flexible, non-stretch, cloth tape measure	Twice (third measure if difference >0.4cm)†	Measure at iliac crest	**zWC:** calculated from WC using UK reference curves.[22]
**Dietary intake**	CADET: A validated 115 item 24-hour food tick list completed for 7 distinct time periods.[24] The CADET tool was developed for use in children aged 3-7 years.[24]	Once (24 hours)	Trained researchers recorded all food and drinks consumed by children during 1 school day. On the same day, children were given a home food diary for completion by a parent/ carer. The following day, trained researchers collected home food diaries and reviewed these with the children. When 1 or more uncompleted sections were identified, the researcher tried to complete these through child dietary recall. Data were processed through the CADET nutrient analysis program at the University of Leeds.	The food and nutrient intake data was used to calculate: **dietary total energy intake (kJ),** and **fruit and vegetable intake (g)** based on the Englyst method.[25]

For peer review only - http://bmjopen.bmj.com/site/about/guidelines.xhtml

**Physical activity**	Actiheart (Cambridge Neurotechnology, Papworth, UK)	Once (worn continuously for 5 days, including 1 weekend)	Attached to the child's chest with 2 ECG electrodes in school by a trained researcher, initialised to record in 30-second epochs	Total movement volume was summarised as average acceleration, along with its underlying movement intensity distribution, using the acceleration and heart rate signals. This was used to calculate: **PA energy expenditure** ¶ and **estimated time spent in MVPA.**

† Where 2 values within ≤ 0.4cm, a definitive measurement value was calculated as the average of the 2. For individuals with 3 values recorded, a definitive measurement value was calculated as the average of the closest pair (within ≤ 0.4cm), or average of all 3 reading (if there were no 2 closest readings, but the differences between values were ≤ 0.4cm). When none of the 3 values were within 0.4cm of each other, no definitive measurement value was calculated.

¶ Children with < 24 hours of valid data over the 5-day measurement period were excluded. To ensure representation across the whole 24-hour period, for those with 24 hours of valid data, only those with a distribution of at least 6 hours in each quadrant (morning 3 am – 9 am; noon 9 am – 3 pm; afternoon 3 pm – 9 pm; and midnight 9 pm – 3 am) were included.

WC, waist-circumference. CADET, Child and Diet Evaluation Tool. zBMI, body mass index z-score. BMI, body mass index. zWC, waist circumference z-score. ECG, electrocardiogram. PA, physical activity. MVPA, moderate-to-vigorous-physical -activity

For peer review only

Assessment of the school environment

Schools participating in the WAVES trial were asked to complete a baseline questionnaire, which enquired about the school's HE and PA environments. The questionnaire was developed to capture the variation in these school environments across the participating schools to assist in interpretation of the main trial findings. Question items were initially developed by the research team and then sent to a health education adviser for review. Questions were refined following feedback from the adviser. The questionnaire was completed by the head teacher or a nominated staff representative and included tick-box questions, followed by open-ended questions to gain further detail regarding policies and practices in the school.

The Health Promoting School framework requirements, government guidelines and prior research were used to develop a scoring system to categorise schools regarding their food/PA environment based on their questionnaire responses. (Supplementary File Table 1).[17,26-29] The scoring system was developed in an iterative way, by the study team, until a consensus was reached on the scoring method and weighting. The use of government publications ensured that the scoring system encompassed key items that have been determined as important for national policy relating to child health at school (for example the provision of 2 hours or more of physical education (PE) per week).[29]

Each question contributed to the final scores. Questions with continuous response variables (such as number of active after school clubs on offer), were categorised into three groups: high; medium; low, using tertile cut-offs with a score allocation of 2;1;0, respectively. Questions with categorical responses were either ordinal or binary (yes/no) questions. Binary questions, such as schools having a healthy eating policy, were scored as 2/0. For the ordinal variables: schools allocating more than two hours to PE was considered of high importance, they were grouped into schools providing this for all years; some years; no years with a score of 2;1;0; while level of support from stakeholders

For peer review only - http://bmjopen.bmj.com/site/about/guidelines.xhtml

(strongly support; support; weakly support) was deemed as less important, so was scored out of 1 (1;0.67;0.33 respectively).

We developed separate HE and PA scores based on a sum of scores from 15 and 20 questions, with the maximum possible score 24 and 34 respectively. The scores for HE and PA were then converted into percentages. The scores ranged from 44-92% for HE and 24-92% for PA. Using the range of scores across all schools, we used tertile cut-offs to split HE and PA scores into three groups. Schools were then categorised according to their environment: i) complete health focus, supporting both HE and PA (top tertile for both HE and PA scores); ii) HE focus (top tertile for HE scores only); iii) PA focus (top tertile for PA scores only); iv) minimal health focus (not in the top tertile for either HE or PA scores).

Data analysis

All analyses were undertaken using STATA Version 15.[30] Descriptive analyses were used to describe pupil characteristics at baseline and both follow-up time points. Categorical variables were summarised as numbers (percentage). Continuous data were summarised as mean (standard deviation) and median (interquartile range (IQR)), as appropriate.

Due to the clustered nature of the data, multilevel linear regression models were used, with school included as a random effect. Primary analysis explored the relationship between the school environment category and pupil outcome measures at the three-study time-points separately. Additionally, a model was fitted using all time-points, allowing for the repeated measures and including a time covariate. Variables included in the model were: baseline age, sex, ethnicity, Index of Multiple Deprivation score and school environment category. Models of follow-up time-points included adjustment for baseline outcome value and the arm of the WAVES trial. The trial arm covariate refers to whether schools were allocated to intervention or control arms in the main WAVES trial. Trial arm was not included in the baseline analyses as participant outcome data at

For peer review only - http://bmjopen.bmj.com/site/about/guidelines.xhtml

this time point were collected before trial arm allocation. Model assumptions were checked using residual plots and the appropriate inclusion of the school random effect was confirmed through likelihood ratio tests. Covariate data were missing for less than 5% of participants, therefore imputation was not performed and a complete case analysis was used.

Patient and public involvement

The WAVES trial research team included a health education advisor (SP) who advised on engagement with schools and school questionnaire design. Input was sought from a head teacher on methods of participant recruitment and participant information materials. The oversight committee for the trial also included a head teacher, who advised on trial conduct throughout.

RESULTS

Of the 54 schools participating in the WAVES trial, 50 (93%) returned the baseline questionnaire. It is these schools and the pupil participants attending them (n=1,304, 94% of the WAVES trial sample) that were included in the current analyses.

Pupil characteristics

Baseline characteristics for pupils are shown in Table 2. The sociodemographic characteristics of the WAVES trial participants excluded from these analyses (due to non-return of school questionnaires) were compared to the study sample and did not differ significantly by age or gender. Those excluded from the analyses were less likely to be of White British ethnicity, and more likely to be of South Asian ethnicity, or in the most deprived Index of Multiple Deprivation score quintile. Anthropometric, dietary and PA characteristics did not differ between those included and those excluded from the analyses.

For peer review only

Table 2: Summary of pupil demographic information at baseline

	Baseline value for those analysed in this study (n=1304)
**Age in years: mean (SD) ; missing data (n)***	6.3 (0.31); 61
**Gender: n (%)**	
**Boys**	654 (50.2)
**Girls**	650 (49.9)
**Missing data***	0
**Ethnicity: n (%)**	
**White British**	656 (50.8)
**South Asian**	333 (25.8)
**African-Caribbean**	100 (7.8)
**Other**	202 (15.7)
**Missing data* (n)**	13
**Deprivation quintile: n (%)†**	
**1 (most deprived)**	639 (50.0)
**2**	266 (20.8)
**3**	146 (11.4)
**4**	119 (9.3)
**5 (least deprived)**	109 (8.5)
**Missing data* (n)**	25
**Deprivation score: mean (SD); missing data (n)***	33.8 (17.9); 25

* Missing data values are not included in the denominator.

† Index of multiple deprivation scores categorised into 5 groups using the national quintile cut-offs for England.

SD, standard deviation.

Outcome measures are summarised for all three time-points in Table 3. Missing data at follow-up 1 and 2 are in part due to attrition of participants as the WAVES trial progressed. The main reasons for attrition were pupils moving away from the school or being absent on the day of data collection. The prevalence of overweight/obesity in the study population was 20.8% at baseline, 26.8% at follow-up-1 (15 months) and 31.1% at follow-up-2 (30 months). Prevalence varied across schools from 8–32% at baseline, 9–47% at follow-up-1 and 9–53% at follow-up-2. There was a statistically significant increase between baseline and follow-up-2 for zBMI and zWC ($p<0.001$). As would be expected, total energy intake increased over the study period as the participating children grew and increased their energy requirement. However, a statistically significant decrease in PAEE and estimated time spent in MVPA ($p<0.001$) was also observed during this period. There was no significant difference in fruit and vegetable intake over time ($p=0.20$).

For peer review only - http://bmjopen.bmj.com/site/about/guidelines.xhtml

Table 3: Summary of pupil outcome measures at 3 timepoints

	Baseline (mean (SD) age = 6.3 (0.3) years)	Follow-up 1 (mean (SD) age = 7.7 (0.3) years)	Follow-up 2 (mean (SD) age = 9.0 (0.3) years)
**Anthropometric**			
**Mean (SD) zBMI; missing data***	0.21 (1.2); 64	0.32 (1.3); 201	0.39(1.3); 293
**BMI group category: n (%)†**			
**Underweight (≤2nd centile)**	29 (2.3)	23 (2.1)	23 (2.3)
**Healthy (>2nd and <85th centiles)**	953 (76.9)	784 (71.1)	674 (66.7)
**Overweight (≥85th and <95th centiles)**	109 (8.8)	126 (11.4)	117 (11.6)
**Obese (≥95th centile)**	149 (12.0)	170 (15.4)	197 (19.5)
**Missing data***	64	201	293
**Median (IQR) zWC; missing data***	0.76 (1.2); 167	0.99 (1.3); 275	1.1 (1.3); 416
**Dietary intake**			
**Median (IQR) dietary total energy intake (kJ/ 24 hours); missing data***	6976.9 (5933.9 – 8101.8); 212	7150.3 (6100.0 – 8279.0); 301	7728.8 (6595.0 – 9128.9); 393
**Median (IQR) fruit and vegetable intake (g/ 24 hours); missing data***	241.8 (149.6 – 352.6); 212	207.8 (111.5 – 320.2); 301	219.5 (117.0 – 346.5); 393
**≥ 5 portion fruit and vegetables: n (%)**			
**Yes**	705 (64.6)	502 (50.1)	519 (57.0)
**No**	387 (35.4)	501 (50.0)	392 (43.0)
**Missing data***	212	301	393
**Physical activity**			
**Mean (SD) PAEE (kJ/kg body weight/ 24 hours); missing data***	94.8 (23.8); 341	91.5 (24.5); 521	79.4 (22.4); 680
**Median (IQR) estimated time spent in MVPA (min/ 24 hours); missing data***	58.1 (41.9 – 81.7); 345	62.0 (39.8 – 101.9); 523	43.1 (31.6 – 64.2); 672

* Missing data values are not included in the denominator.

† Based on UK 1990 reference centile curves and applying cut-offs used for population monitoring.

SD, standard deviation. IQR, interquartile range. zBMI, body mass index z-score. BMI, body mass index. zWC, waist circumference z-score. PAEE, physical activity energy expenditure. MVPA, moderate-to-vigorous physical activity.

For peer review only - http://bmjopen.bmj.com/site/about/guidelines.xhtml

School characteristics

All schools had some activities or policies that promoted HE or PA. Overall schools gained higher scores for PA than HE promotion, with the mean normalised score (adjusted for number and type of questions) being 76% (IQR: 66–85%) for PA and 67% (IQR: 51–74%) for HE. Eight (16%) schools were classed as PA focus, 10 (20%) as HE focus, 10 (20%) as complete health focus and 22 (44%) as minimal health focus. The application of the scoring system to schools based on their baseline questionnaire responses, including information on missing data, is shown in Table 4.

For peer review only

Table 4: Summary of the scoring of schools on healthy eating and physical activity based on baseline questionnaire responses

Description of variable	Number of schools (%)		
**Healthy eating score**			
	**No (score=0)**	**Yes (score =2)**	
School has a policy regarding HE*	11 (22.5)	38 (77.6)	
HE is promoted in school curriculum	10 (20.0)	40 (80.0)	
School cookery club to promote HE	29 (58.0)	21 (42.0)	
Involve parents in the promotion of HE	27 (54.0)	23 (46.0)	
Drinking water is provided throughout the day*	0 (0.0)	49 (100.0)	
Breakfast club is available for students	15 (30.0)	35 (70.0)	
Food provided by the school at break deemed as healthy*	32 (65.3)	17 (34.7)	
	**Low (score=0)**	**Medium (score =1)**	**High (score =2)**
Provide a range of ways to promote HE	12 (24.0)	13 (26.0)	25 (50.0)
Proportion of children choosing to eat school lunches†	12 (32.4)	11 (29.7)	14 (37.8)
	**Weakly support (score=0.33)**	**Support (score=0.67)**	**Strongly support (score=1)**
Governors support HE in school	2 (4.0)	31 (62.0)	17 (34.0)
Senior Leadership Team support HE in school*	0 (0.0)	21 (42.9)	28 (57.1)
Staff support HE in school*	0 (0.0)	27 (55.1)	22 (44.9)
School council support HE in school*	2 (4.1)	25 (51.0)	22 (44.9)
Pupils support HE in school*	5 (10.2)	35 (71.4)	9 (18.4)
Parents support HE in school*	14 (28.6)	31 (63.3)	4 (8.2)

Description of variable	Number of schools (%)		
**Physical activity score**			
	**No (score=0)**	**Yes (score=2)**	
School has a policy regarding PA**	3 (6.3)	45 (93.8)	
School has a PA co-ordinator***	4 (8.5)	43 (91.5)	
Staff are trained regarding PA***	17 (36.2)	30 (63.8)	
Has sport partnerships and community links***	11 (23.4)	36 (76.6)	
PA is promoted in the school curriculum**	6 (12.5)	42 (87.5)	
Involve parents in the promotion of PA**	32 (66.7)	16 (33.3)	
School has a walk to school campaign**	13 (27.1)	35 (72.9)	
PE is taught by a specialist**	21 (43.8)	27 (56.3)	
Range of activities offered in PE meet Department of Education recommendations**	3 (6.3)	45 (93.8)	
	**Low (score=0)**	**Medium (score=1)**	**High (score=2)**
Provide a range of ways to promote PA**	7 (14.6)	20 (41.7)	21 (43.8)
Variety of PA opportunities other than PE**	10 (20.8)	15 (31.3)	23 (47.9)
Have a range of play equipment to be used**	12 (25.0)	18 (37.5)	18 (37.5)
Provide a range of sports clubs**	12 (25.0)	11 (22.9)	25 (52.1)
	**For no years (score=0)**	**For some years (score=1)**	**For all years (score=2)**
Schools allocating 2 or more hours to PE per week **	7 (14.6)	2 (4.2)	39 (81.3)
	**Weakly support (score=0.33)**	**Support (score=0.67)**	**Strongly support (score=1)**
Governors support PA in school**	0 (0.0)	28 (58.3)	20 (41.7)
Senior Leadership Team support PA in school**	0 (0.0)	18 (37.5)	30 (62.5)
Staff support PA in school**	0 (0.0)	22 (45.8)	26 (54.2)
School council support PA in school***	0 (0.0)	24 (51.1)	23 (48.9)
Pupils support PA in school**	0 (0.0)	22 (45.8)	26 (54.2)
Parents support PA in school**	4 (8.3)	33 (68.8)	11 (22.9)

* Missing data from 1 school, ** Missing data from 2 schools, *** Missing data from 3 schools, † Missing data from 13 schools.

Missing data values are not included in the denominator used for percentage calculations.

HE, healthy eating. PA, physical activity. PE, Physical Education.

Not all schools reported having HE or PA policies (11 (22%) with no HE policy; 3 (6%) with no PA policy); however, several of these schools were placed in the PA focus, HE focus or complete health focus categories as they scored highly in other aspects of healthy food and PA promotion activity. All schools provided drinking water throughout the school day. Thirty-five (70%) had a

For peer review only - http://bmjopen.bmj.com/site/about/guidelines.xhtml

breakfast club available to students. However, it was difficult to assess how healthy the food provided was from the information available and only 17 schools (35%) provided a healthy break snack. Forty-five schools (94%) offered a range of activities during PE that met the Department of Education recommendations and 39 (81%) allocated two or more hours to PE per week. However, PE was taught by a specialist in only 27 schools (56%). Parents were perceived to support the HE and PA environment less than other stakeholders in the school. In addition, only 23 (46%) and 16 (33%) of schools involved parents in HE and PA promotion, respectively.

Associations between the school food/PA environment category and childhood anthropometric, diet and PA outcomes

For all models (i.e. models at each time-point and repeated measures models for each outcome), the regression coefficients (B) for the school environment categories are presented in Table 5, with the minimal health focus category as the reference. Regression coefficients for all fixed-effect covariates in the models are presented in Supplementary File Tables 2-8. School environment category was not statistically significantly associated with child zBMI at baseline or follow-up-1. At follow-up-2, compared with minimal health focus schools, pupils in PA focus schools had a lower zBMI (B=-0.17, 95% CI -0.31 to -0.03, p=0.02). Although not statistically significant, compared to those in schools with minimal health focus, children in schools with a complete health focus also had a lower zBMI at follow-up-2 (B=-0.11, 95% CI -0.25 to 0.03, p=0.14). At follow-up-2, PA focus and complete health focus schools also had a lower zWC than minimal health focus schools, but this was non-significant (PA focus: B=-0.15, 95% CI -0.36 to 0.06, p=0.16 and complete health focus: B=-0.18, 95% CI -0.38 to 0.03, p=0.09). Compared with minimal health focus schools, PA focus and the HE focus schools had lower zBMI and zWC in the repeated measures models, but these were non-significant.

For peer review only - http://bmjopen.bmj.com/site/about/guidelines.xhtml

Children in schools in HE, PA and complete health focus categories had significantly lower daily PAEE at baseline than those in minimal health focus schools (PA focus: B=-6.0, 95% CI -11.7 to -0.28, p=0.04; HE focus: B=- 6.3, 95% CI -11.8 to – 0.86, p=0.02; and complete health focus: B=-6.3, 95% CI -11.6 to -1.0, p=0.02). They also did less MVPA at baseline, however, this was only significant for schools with a complete health focus (B=-14.1, 95% CI -27.4 to -0.75, p=0.04). By follow-up-2, this trend reversed, with higher levels of both PAEE and MVPA in the HE, PA and complete health focus schools, compared with minimal health focus schools, although these findings were not significant. We found no significant differences in PAEE across school environment categories in the repeated measures model.

School environment category was not significantly associated with total dietary energy intake or fruit and vegetable intake in any of the models.

For peer review only

Table 5: The association between the school food/physical activity environment category and pupil obesity, dietary and physical activity outcome measures, estimated using multilevel modelling

Outcome variable	School environment category *	Outcome time point							
		Baseline		Follow-up-1		Follow-up-2		Repeated measures model	
		Regression coefficient (95% CI)	p-value	Regression coefficient (95% CI)	p-value	Regression coefficient (95% CI)	p-value	Regression coefficient (95% CI)	p-value
**zBMI**	**PA focus**	-0.06 (-0.25, 0.14)	0.57	-0.06 (-0.22, 0.10)	0.48	**-0.17 (-0.31, -0.03)**	**0.02**	-0.10 (-0.29, 0.10)	0.32
	**HE focus**	-0.09 (-0.28, 0.09)	0.33	-0.02 (-0.18, 0.15)	0.86	-0.02 (-0.16, 0.13)	0.80	-0.07 (-0.27, 0.13)	0.47
	**Complete health focus**	0.04 (-0.14, 0.23)	0.64	0.07 (-0.09, 0.23)	0.41	-0.11 (-0.25, 0.03)	0.14	0.07 (-0.12, 0.26)	0.49
**zWC**	**PA focus**	-0.10 (-0.36, 0.16)	0.44	0.04 (-0.22, 0.30)	0.75	-0.15 (-0.36, 0.06)	0.16	-0.11 (-0.32, 0.11)	0.33
	**HE focus**	-0.24 (-0.48, 0.01)	0.06	0.19 (-0.07, 0.45)	0.14	0.07 (-0.15, 0.29)	0.55	-0.14 (-0.37, 0.08)	0.21
	**Complete health focus**	0.10 (-0.14, 0.34)	0.40	-0.01 (-0.26, 0.24)	0.95	-0.18 (-0.38, 0.03)	0.09	0.07 (-0.14, 0.28)	0.52
**Dietary total energy intake (kJ/24 hours)**	**PA focus**	-903.5 (-2402.7, 595.6)	0.24	433.7 (-70.5, 937.9)	0.09	33.2 (-614.5, 681.0)	0.92	-220.9 (-815.1, 373.4)	0.47
	**HE focus**	-847.8 (-2255.8, 560.3)	0.24	-63.7 (-598.0, 470.5)	0.82	260.8 (-443.1, 964.7)	0.47	-483.8 (-1087.4, 119.9)	0.12
	**Complete health focus**	-399.3 (-1801.3, 1002.6)	0.58	32.8 (-464.7, 530.3)	0.90	171.3 (-466.4, 809.1)	0.60	-200.0 (-773.9, 373.8)	0.49
**Fruit and vegetable intake (g/24 hours)**	**PA focus**	-57.8 (-135.5, 19.8)	0.14	11.8 (-27.9, 51.5)	0.56	-16.0 (-73.9, 41.9)	0.59	-22.6 (-64.9, 19.6)	0.29
	**HE focus**	-31.6 (-104.7, 41.4)	0.40	3.0 (-38.6, 44.7)	0.89	-4.4 (-66.9, 58.0)	0.89	-21.6 (-64.4, 21.2)	0.32
	**Complete health focus**	-7.3 (-80.0, 65.4)	0.84	27.1 (-11.9, 66.0)	0.17	8.6 (-48.1, 65.3)	0.77	5.2 (-35.6, 45.9)	0.80

For peer review only - http://bmjopen.bmj.com/site/about/guidelines.xhtml

Outcome variable	School environment category *	Outcome time point							
		Baseline		Follow-up-1		Follow-up-2		Repeated measures model	
		Regression coefficient (95% CI)	p-value	Regression coefficient (95% CI)	p-value	Regression coefficient (95% CI)	p-value	Regression coefficient (95% CI)	p-value
**PAEE (kJ/kg body weight/24 hours)**	**PA focus**	**-6.0 (-11.7, -0.28)**	**0.04**	-0.79 (-7.2, 5.7)	0.81	1.9 (-3.8, 7.6)	0.51	-2.8 (-7.2, 1.6)	0.21
	**HE focus**	**-6.3 (-11.8, -0.86)**	**0.02**	3.8 (-3.1, 10.7)	0.28	3.5 (-2.7, 9.7)	0.27	-0.83 (-5.3, 3.7)	0.72
	**Complete health focus**	**-6.3 (-11.6, -1.0)**	**0.02**	-2.6 (-8.8, 3.6)	0.42	**5.3 (0.04, 10.6)**	**0.05**	-3.0 (-7.2, 1.2)	0.16
**Estimated time spent in MVPA (min/24 hours)**	**PA focus**	-4.2 (-18.5, 10.1)	0.57	-3.7 (-22.7, 15.2)	0.70	3.8 (-10.5, 18.2)	0.60	-1.4 (-11.9, 9.1)	0.80
	**HE focus**	-13.2 (-26.8, 0.47)	0.06	-0.80 (-20.9, 19.3)	0.94	0.78 (-14.5, 16.1)	0.92	-5.0 (-15.7, 5.7)	0.36
	**Complete health focus**	**-14.1 (-27.4, -0.75)**	**0.04**	-6.4 (-24.7, 11.8)	0.49	7.2 (-6.4, 20.9)	0.30	-6.4 (-16.5, 3.7)	0.21

* Minimal health focus schools are the reference category.

All models with outcomes measured at a single time point include school as a random effect and age at baseline, sex, ethnicity, and index of multiple deprivation score as fixed-effect covariates. Models with outcomes at follow-up-1 and follow-up-2 also include the outcome variable at baseline and the WAVES trial arm as fixed-effect covariates.

Repeated measures models include school and pupil as a random effect, and measurement time point, age at baseline, sex, ethnicity, index of multiple deprivation score and WAVES trial arm as fixed-effect covariates.

Results shown in bold are statistically significant.

95% CI, 95% confidence interval. zBMI, body mass index z-score. zWC, waist circumference z-score. PAEE, physical activity energy expenditure. MVPA, moderate-to-vigorous physical activity. PA, physical activity. HE, healthy eating.

For peer review only - http://bmjopen.bmj.com/site/about/guidelines.xhtml

DISCUSSION

The questionnaire responses indicated that all schools promoted healthy behaviours to some degree, but there was variation in the extent of their efforts. The school scores indicated that schools tended to focus more on promoting PA than HE. This may relate to increased government focus and spending on PA in schools during the study period.[31] Overall, we found little relationship between the school food/PA environment and pupil anthropometric measures, diet or PA levels. Most associations between the school environment category and the outcome measures were non-significant, and those that were significant were small in magnitude. However, by the second follow-up time point (30 months after baseline) there was a statistically significant lower mean zBMI among pupils in schools with a PA focus, compared with those in schools with minimal health focus. Although the effect size is small (mean difference= -0.17; 95% CI -0.31 to -0.03; p= 0.02), if successfully sustained it has the potential to produce public health benefits at a population level.[32] A similar finding was observed for zWC, although this was non-significant. In line with these associations between school environment activity and obesity-related outcomes, we found that at the second follow-up time point PAEE levels were higher in schools with a complete health focus compared with those with minimal health focus. Therefore, the findings may suggest that a healthy school environment could have an effect over time. In this study, we used baseline data collected when the participants had already been at school for a year, so it is possible that if we had baseline data from before school entry, we would have seen a larger association between school environment and obesity and physical activity outcomes.

Strengths

To our knowledge, this is the first UK study to assess the association between primary school food and PA promoting environments and child obesity measures longitudinally. The study involves a large sample, from an ethnically and socio-economically diverse population, over a three-year period. Outcomes were predominantly assessed by objective measures, using validated instruments

For peer review only - http://bmjopen.bmj.com/site/about/guidelines.xhtml

and standardised procedures. We accounted for clustering and individual level confounders by using multilevel models.

Limitations

The sample includes only 50 primary schools that consented to be part of an obesity prevention trial and parental consent was only obtained for 60% of eligible children within these schools. Our study sample may therefore represent schools with more interest in health promotion and pupils who were healthier. However, obesity prevalence in the participating schools was compared with the prevalence estimated from the National Child Measurement Programme at the time and was found to be similar.[33] Comparisons of demographic characteristics of children with and without parental consent to participate in the WAVES trial (undertaken as part of the WAVES trial) also did not show major differences,[34] suggesting that the study sample is broadly representative.
Further limitations relate to participant outcome measurement and attrition. There was attrition between each measurement time point. A higher proportion of participants of African/Caribbean ethnicity and participants from more deprived groups had missing outcome data at the follow up time points, which may partially account for different findings in the follow-up models compared with the baseline models. Limiting analyses to participants with data at all time points would potentially help us explore this, however, we have not conducted these analyses as they would only involve a small, unrepresentative sample.

The questionnaire used to assess the school environment was developed by the WAVES trial research team with input from a health education adviser to capture variation in the environments of the participating schools. It was assessed for face validity, but not further validated. Schools completed the questionnaire with a varying degree of completeness; therefore, the resulting information may not fully reflect their activity relating to the promotion of healthy eating and PA. The methods for scoring schools in relation to HE and PA were developed by the authors, based on

For peer review only

past and present guidelines relating to health promoting schools. However, the construct validity of these could not be meaningfully explored through confirmatory factor analysis due to the small number of schools in our sample.[35] Furthermore, the categorisation process of schools was pragmatically determined based on statistical cut-offs, and there may be large variations in health promoting activities within each of the categories. It is possible that, due to limitations of the baseline questionnaire and the lack of validation of the scoring system, the schools' food and PA promoting environments may not have been accurately represented, which in turn may have led to the equivocal findings of the study. In addition, the school environment was only assessed at 1 time point, and it is possible that support for HE or PA in the schools changed over time. Therefore, we have not been able to assess longitudinal changes in the school environment and how these are associated with obesity, diet and PA outcomes.

We explored associations between the school environment and multiple outcomes in this study. It is therefore possible that the statistically significant associations that we found could be due to multiple testing. However, as this was an exploratory, hypothesis generating study, we did not apply a correction for multiple testing as these tend to be conservative and may have led us to miss potential differences between groups that warrant further research.[36] The Child And Diet Evaluation Tool (CADET) was quick and practical with a relatively low response burden,[37] but it only measured dietary intake over a single day, so may not be an accurate assessment of habitual intake.

Finally, the data used in this study were drawn from a randomised controlled trial evaluating a childhood obesity prevention intervention and included data from both intervention and control arms. Although there was no evidence of effectiveness of the intervention from the main trial,[20] we adjusted for the intervention/control status of participants in our analyses. However, even with these adjustments we cannot rule out the possibility of marginal intervention effects influencing the

For peer review only - http://bmjopen.bmj.com/site/about/guidelines.xhtml

association between school food and PA environments and anthropometric, physical activity and dietary outcomes.

Comparison with previous research

This study brings new information regarding the potential impact the school HE and PA environment has on children, as previous research has focused on the effectiveness of specific interventions and policies within schools.

Our findings are in line with those from two systematic reviews that found moderate evidence that school PA interventions are able to reduce BMI.[11,13] Our study included a range of school level activities, rather than focusing on specific interventions, and the effect size observed at 30 months was similar to the summary estimate from the trials.[11]

There is a large pool of research indicating PA interventions increase PA levels in children during the school day,[12-15] although overall PA levels may not be increased.[16] We found that children in schools with an increased focus on PA were less active and engaged in less MVPA at baseline compared with minimal health focus schools, but over time the trend was reversed, suggesting that school PA promotion may increase child PA levels over time. Our findings may also indicate that PA promotion in schools only affects PA levels as children get older, when their PA levels generally decrease.

Previous systematic reviews have not found promising results regarding the impact of interventions on dietary outcomes, although there is some evidence that they are able to increase fruit and vegetable intake.[13,17] This study did not find a relationship between the school environment and child dietary outcomes. The differences between the results of this study and previous research may be as a result of assessing overall environment, rather than specific targeted interventions. In 2018,

For peer review only - http://bmjopen.bmj.com/site/about/guidelines.xhtml

For peer review only

the House of Commons (UK Parliament) stated that there was no convincing evidence the school healthy food standards had been implemented effectively.[38] The majority (n=32, 65%) of schools in this study provided a break-time snack which was deemed unhealthy, suggesting other school food provision may also be unhealthy. Furthermore, the limitations of the questionnaire in assessing school healthy eating environment and those related to the dietary assessment method may also have contributed to differences in findings.

In terms of healthy food and PA promoting environments, we found that school perception of parental support for HE in schools was not high (low parental support perceived by over a quarter of schools). Children consistently refer to home life being more significant in changing behaviour than schools and families have been shown to be pivotal in ensuring improvements to lifestyle in primary school aged children.[39-41] Our findings suggest that in relation to PA and HE, parent-school relationships need strengthening. Nevertheless, the complexity of promoting a healthy school environment and of including parents in such endeavours also need to be considered.[42]

Implications

Consistent with previous research, this study indicates that supporting PA in primary schools may have beneficial health effects in terms of increasing PA levels and reducing weight status. Although schools often prioritise academic attainment over PE and PA, increasing PE in the curriculum has not been found to have negative academic consequences and may improve school achievement.[43,44] Therefore, schools are recommended to increase their efforts to promote PA.

In this study, school environments were not as supportive of HE as expected, which is in line with UK Parliament's report stating that there was a lack of effective implementation of school food standards legislation.[38] Stronger enforcement of existing school food regulation and further regulatory measures may be needed to ensure HE is fully supported in schools.

For peer review only - http://bmjopen.bmj.com/site/about/guidelines.xhtml

All associations between the school food/PA environment and health/behavioural outcomes were of small magnitude, which suggests it has an important but limited role in child health. Parents are key influencers of children's health,[40,41] but in this study the majority of schools did not involve parents in their health promotion efforts. The development of school food and PA promoting environments may be more effective if schools develop better relationships with parents and involve them in their health promotion efforts.

Finally, the changes in health behaviours that we observed were at follow-up-2, once the children had been exposed to the school environment for three years. This may indicate that future intervention studies should have a longer follow-up periods to evaluate their effectiveness.

Conclusion

We found that over time, a school environment supportive of PA may positively influence zBMI and that schools with a more food and PA promoting environment may contribute to increased PA levels in children over time. More research is needed to further understand the influence of the school environment, but in the meantime, schools should be encouraged to provide an environment that is supportive of PA and HE. However, schools cannot undertake health promotion in isolation. They need to be considered as part of a complex system, which requires intervention at multiple points to produce a sustained reduction in child overweight and obesity.

For peer review only

For peer review only - http://bmjopen.bmj.com/site/about/guidelines.xhtml

ACKNOWLEDGEMENTS

We would like to acknowledge the contribution of all the WAVES study trial investigators: University of Birmingham: Peymane Adab (Professor of Chronic Disease Epidemiology & Public Health and Chief Investigator), Tim Barrett (Leonard Parsons Professor of Paediatrics and Child Health), KK Cheng (Professor of Public Health and Primary Care), Jonathan J Deeks (Professor of Biostatistics), Joan L Duda (Professor of Sport and Exercise Psychology), Emma Frew (Professor in Health Economics), Karla Hemming (Professor of Biostatistics), Miranda J Pallan (Reader in Public Health & Epidemiology), Jayne Parry (Professor of Policy and Public Health); University of Cambridge, Cambridge MRC Epidemiology Unit / Norwegian School of Sport Sciences: Ulf Ekelund (Professor in Physical Activity and Health); University of Leeds: Janet E Cade (Professor of Nutritional Epidemiology and Public Health); Loughborough University: Amanda Daley (Professor of Behavioural Medicine); University of Edinburgh: Raj Bhopal (Emeritus Professor of Public Health); University of Warwick: Paramjit Gill (Professor of General Practice); Birmingham Community Healthcare NHS Trust: Eleanor McGee (Public Health Nutrition Lead); Birmingham Services for Education: Sandra Passmore (Education Advisor)

We thank the children, school staff, and parents who participated in the trial; the study team, including Behnoush Mohammadpoor Ahranjani and Emma Popo who helped in overseeing the study measurements and data collection; the administrative team who facilitated the running of the project; the research staff who undertook the study measurements; and Robert Lancashire who developed the trial database and oversaw data management. We also thank our collaborators at the Institute of Metabolic Science at the Medical Research Council (MRC) Epidemiology Unit (University of Cambridge) for their support and assistance, particularly Soren Brage, Kate Westgate, and Stefanie Hollidge, for training of field staff on use and maintenance of Actihearts, for overseeing data preparation, and for analysis and interpretation of the physical activity data; Timo Lakka (principal investigator of the PANIC study, University of Eastern Finland) for sharing

For peer review only

exercise data in a similarly aged cohort of children to allow translation of observed heart rate into activity energy expenditure; staff who are working, or have previously worked, at the Nutrition Epidemiology Group in Leeds, who have supported the trial team in terms of administration of the CADET food tick list and processing of the data to enable us to undertake dietary intake analysis, in particular Neil Hancock, Cristina Cleghorn, Meagan Christian, Jayne Hutchinson, Holly Rippin, and Catherine Rycroft. We also thank Sayeed Haque for his statistical advice on the analyses for this particular study.

HUMAN SUBJECTS APPROVAL STATEMENT

The WAVES trial was approved by NHS Research Ethics Service Committee West Midlands, The Black Country (NHS REC Number: 10/H1202/69) and sponsored by the University of Birmingham (Sponsor Number: RG_10-183). Parents gave written informed consent for children to participate and children gave verbal assent before measurements were taken. Permission to access the WAVES study data for this study was obtained in October 2018.

TRIAL MONITORING AND DATA MONITORING

The trial steering committee met annually and included: chair/statistician: Kelvin Jordan, Keele University; subject expert: Peter Whincup, St George's, University of London; health economist: Louise Longworth, Brunel University; public representative: John Bennett, PHSE advisor; and Chief Investigator: Peymane Adab, University of Birmingham.

FUNDING

This WAVES trial was funded by the National Institute for Health Research (NIHR) Health Technology Assessment Programme (project reference 06/85/11). The views expressed are those of the authors and not necessarily those of the NIHR or the Department of Health and Social Care.

For peer review only - http://bmjopen.bmj.com/site/about/guidelines.xhtml

COMPETING INTERESTS

Miranda Pallan, Tania Griffin, Kiya Hurley, Emma Lancashire, Alice Sitch and Peymane Adab hold grants from the UK National Institute for Health Research, and Peymane Adab is Chair of the National Institute for Health Research Public Health Research Funding Committee. There are no other competing interests to declare.

AUTHOR CONTRIBUTION

Elizabeth Mairenn Garden (first author) designed and undertook the analysis reported in this manuscript, and drafted the manuscript. Miranda Pallan and Peymane Adab designed and oversaw the conduct of the trial from which the data is drawn, advised on the design and analysis of the reported study, and also contributed substantially to the manuscript. Joanne Clarke, Tania Griffin, Kiya Hurley and Emma Lancashire all designed school and pupil data collection tools and undertook data collection. They also substantially contributed to the manuscript. Alice Sitch assisted in developing the analysis plan, conducted parts of the analyses and substantially contributed to the manuscript. Sandra Passmore oversaw the main trial from which the data is drawn, advised on the development of the school data collection tool, and substantially contributed to the manuscript, bringing a school policy perspective. All authors approved the final version of the manuscript.

DATA SHARING STATEMENT

Requests for access to data from the WAVES trial should be addressed to the Principal Investigator at p.adab@bham.ac.uk. All proposals requesting data access will need to specify how it is planned to use the data, and all proposals will need approval of the trial co-investigator team before data release.

For peer review only

For peer review only - http://bmjopen.bmj.com/site/about/guidelines.xhtml

REFERENCES

1. Mead E, Brown T, Rees K, et al. Diet, physical activity and behavioural interventions for the treatment of overweight or obese children from the age of 6 to 11 years. *Cochrane Database Syst Rev* 2017; Issue 6. Art No.:CD012651 doi:10.1002/14651858.CD012651.
2. Sahoo K, Sahoo B, Choudhury AK, et al. Childhood obesity: causes and consequences. *J Family Med Prim Care* 2015;4(2):187–92 doi:10.4103/2249-4863.154628.
3. Rutter H. Overweigh and obesity: where are we and where are we heading?. National Obesity Observatory. 2012. Available from: https://www.england.nhs.uk/wp-content/uploads/2012/05/Obesity-Review-Group-19-April-2012-Background-Slide-Set-1-Prevalence-and-Trends.pdf. Accessed: 10 Oct 2020.
4. Stats Team. National child measurement programme England, 2017/ 2018 School Year. 2018. Available from: https://files.digital.nhs.uk/9F/22AF4D/nati-chil-meas-prog-eng-2017-2018-rep.pdf. Accessed: 22 Jan 2019.
5. Butland B, Jebb S, Kopelman P, et al. Tackling obesities: future choices-project report 2nd Edition. Government Office for Science. 2007. Available from: https://assets.publishing.service.gov.uk/government/uploads/system/uploads/attachment_data/file/287937/07-1184x-tackling-obesities-future-choices-report.pdf. Accessed: 25 Oct 2018.
6. Kipping RR, Payne C, Lawlor DA. Randomised controlled trial adapting US school obesity prevention to England. *Arch Dis Child* 2008;93(6):469-73 doi:10.1136/adc.2007.116970.
7. Public Health England. Health matters: obesity and the food environment. 2017. Available from: https://www.gov.uk/government/publications/health-matters-obesity-and-the-food-environment/health-matters-obesity-and-the-food-environment--2. Accessed: 12 Nov 2018.
8. Huang TT, Drewnosksi A, Kumanyika S, et al. A systems-oriented multilevel framework for addressing obesity in the 21st century. *Prev Chronic Dis* 2009;6(3):A82.

For peer review only - http://bmjopen.bmj.com/site/about/guidelines.xhtml

9. Rutter H. The single most important intervention to tackle obesity…. *Int J Public Health* 2012;57(4):657-8 doi:10.1007/s00038-012-0385-6.

10. Johnston L, Matteson C, Finegood D. Systems science and obesity policy: a novel framework for analyzing and rethinking population-level planning. *Am J Public Health* 2014;104(7):1270-8 doi:10.2105/AJPH.2014.301884.

11. Brown T, Moore THM, Hooper L, et al. Interventions for preventing obesity in children. *Cochrane Database Syst Rev* 2019, Issue 7. Art No.:CD001871 doi:10.1002/14651858.CD001871.pub4.

12. Dobbins M, DeCorby K, Robeson P, et al. School-based physical activity programs for promoting physical activity and fitness in children and adolescents aged 6 – 18. *Cochrane Database Syst Rev* 2013, Issue 2. Art No.: CD007651 doi:10.1002/14651858.CD007651.pub2.

13. Langford R, Bonell C, Jones H, et al. The WHO health promoting school framework for improving the health and well-being of students and their academic achievement. *Cochrane Database Syst Rev* 2014, Issue 4. Art No.: CD008958 doi:10.1002/14651858.CD008958.pub2.

14. Drummy C, Murtagh EM, McKee DP, et al. The effect of a classroom activity break on physical activity levels and adiposity in primary school children. *J Paediatr Child Health* 2016;52(7):745–9 doi:10.1111/jpc.13182.

15. Wilkie HJ, Standage M, Gillison FB, et al. Correlates of intensity-specific physical activity in children aged 9-11 years: a multilevel analysis of UK data from the international study of childhood obesity, lifestyle and the environment. *BMJ Open* 2018;8(2):e018373–e018373 doi:10.1136/bmjopen-2017-018373.

16. Love R, Adams J, van Sluijs EM. Are school-based physical activity interventions effective and equitable? A meta-analysis of cluster randomised controlled trials with accelerometer-assessed activity. *Obes Rev* 2019;20(6):859-70 doi:10.1111/obr.12823.

For peer review only - http://bmjopen.bmj.com/site/about/guidelines.xhtml

17. Micha R, Karageorgou D, Bakogianni I, et al. Effectiveness of school food environment policies on children's dietary behaviors: a systematic review and meta-analysis. *PLoS One* 2018;13(3):e0194555–e0194555 doi:10.1371/journal.pone.0194555.

18. Harrison F, Bentham G, Jones AP, et al. School level correlates with adiposity in 9-10 year old children. *Health Place* 2011;17(3):710-6 doi:10.1016/j.healthplace.2011.01.009.

19. Pallan MJ, Adab P, Sitch AJ, et al. Are school physical activity characteristics associated with weight status in primary school children? A multilevel cross-sectional analysis of routine surveillance data. *Arch Dis Child* 2014;99(2):135-41 doi:10.1136/archdischild-2013-303987.

20. Adab P, Pallan MJ, Lancashire ER, et al. Effectiveness of a childhood obesity prevention programme delivered through schools, targeting 6 and 7 year olds: cluster randomised controlled trial (WAVES study). *BMJ* 2018;360:k211 doi:10.1136/bmj.k211

21. Department for Communities and Local Government. The English indices of deprivation 2010. 2011. Available from: https://assets.publishing.service.gov.uk/government/uploads/system/uploads/attachment_data/file/6871/1871208.pdf. Accessed: 22 Jan 2019.

22. Cole TJ, Freeman JV, Preece MA. Body mass index reference curves for the UK, 1990. *Arch Dis Child* 1995;73(1):25–9 doi:10.1136/adc.73.1.25

23. World Health Organization. Physical status: the use and interpretation of anthropometry [WHO technical report series 854]. Geneva 1995. Available from: https://www.who.int/childgrowth/publications/physical_status/en/. Accessed: 8 Feb 2019.

24. Cade JE, Frear L, Greenwood DC. Assessment of diet in young children with an emphasis on fruit and vegetable intake: using CADET – Child and Diet Evaluation Tool. *Public Health Nutr* 2006; 9(4):501–8 doi: 10.1079/phn2005871.

25. Englyst HN, Cummings JH. Improved method for measurement of dietary fiber as non-starch polysaccharides in plant foods. *J Assoc Off Anal Chem* 1988; 71(4):808—814.

For peer review only

26. The requirements of school food standards 2014 (SI 2014/1603). 2014. Available from: http://www.legislation.gov.uk/uksi/2014/1603/contents/made. Accessed: 22 Jan 2019.

27. Department of Health and Department for Children, Schools and Families. Introduction to the national healthy schools programme. Available from: http://dera.ioe.ac.uk/6798/7/Introduction_Redacted.pdf. Accessed: 27 Oct 2018.

28. Evans C EL, Mandl V, Christian MS, et al. Impact of school lunch type on nutritional quality of English children's diets. *Public Health Nutr* 2016;19(1):36–45 doi:10.1017/S1368980015000853.

29. Department for Education and Skills. Learning through PE and sport. Available from: https://webarchive.nationalarchives.gov.uk/20130402163246/https:/www.education.gov.uk/publications/eOrderingDownload/LTPES.pdf.pdf. Accessed: 10 Oct 2020.

30. Stata Corp. Stata statistical software: release 15. College station, TX: Stata Corp LLC; 2017.

31. The Office for Standards in Education, Children's Services and Skills (Ofsted). The PE and sport premium for primary schools. Available from: https://www.gov.uk/government/publications/the-pe-and-sport-premium-for-primary-schools. Accessed: 20 November 2020.

32. Rose G. Sick individuals and sick populations. *Int J Epidemiol* 1985;14(1):32–8 doi: 10.1093/ije/14.1.32.

33. Conolly A. Health survey for England 2015 children's body mass index, overweight and obesity. NHS Digital [ISBN: 978-1-78386-896-4]. 2016. Available from: http://healthsurvey.hscic.gov.uk/media/37776/hse2015-child-obe.pdf. Accessed: 22 Jan 2019.

34. Adab P, Barrett T, Bhopal R, et al. The West Midlands ActiVe lifestyle and healthy Eating in School children (WAVES) study: a cluster randomised controlled trial testing the clinical effectiveness and cost-effectiveness of a multifaceted obesity prevention intervention

For peer review only

programme targeted at children aged 6–7 years. Southampton (UK): NIHR Journals Library; 2018 Feb. (Health Technology Assessment, No. 22.8.) doi:10.3310/hta22080.

35. Wolf EJ, Harrington KM, Clark SL, et al. Sample size requirements for structural equation models: an evaluation of power, bias, and solution propriety. *Educ Psychol Meas* 2013;73:913-34 doi: 10.1177/0013164413495237.

36. Rothman KJ. No adjustments are needed for multiple comparisons. *Epidemiology* 1990;1(1):43-6.

37. Zaborskis A, Moceviciene R, Iannotti RJ. The influence of chronological period of data collection on differences in reported dietary intake among school-aged children surveyed in 39 countries. *J Nutr Educ Behav* 2014;46(5):359–69 doi:10.1016/j.jneb.2013.11.017.

38. Health committee. Childhood obesity: time for action (HC 882). London: The Stationery Office; 2018.

39. Fairbrother H, Curtis P, Goyder E. Where are the schools? Children, families and food practices. *Health Place* 2016;40:51-7 doi:10.1016/j.healthplace.2016.04.013.

40. Golan M, Weizman A. Familial approach to the treatment of childhood obesity: conceptual model. *J Nutr Educ* 2001;33(2):102–7 doi:10.1016/s1499-4046(06)60173-5.

41. Esposito L, Fisher JO, Mennella JA, et al. Developmental perspectives on nutrition and obesity from gestation to adolescence. *Prev Chronic Dis* 2009;6(3):A94.

42. Passmore S, Jones L. A review of the sustainability and impact of a healthy lifestyles programme in primary schools 2–5 years after the intervention phase. *Health Educ Res* 2019;34(1):72-83 doi:10.1093/her/cyy043.

43. Story M, Nanney MS, Schwartz MB. Schools and obesity prevention: creating school environments and policies to promote healthy eating and physical activity. *Milbank Q* 2009; 87(1):71–100 doi:10.1111/j.1468-0009.2009.00548.x.

44. Martin A, Booth JN, Laird Y, et al. Physical activity, diet and other behavioural intervention for improving cognition and school achievement in children and adolescents with obesity or

For peer review only - http://bmjopen.bmj.com/site/about/guidelines.xhtml

overweight. *Cochrane Database Syst Rev* 2018, Issue 3. Art. No.: CD009728 doi:10.1002/14651858.CD009728.pub4.

For peer review only

Relationship between primary school healthy eating and physical activity promoting environments and children's dietary intake, physical activity and weight status: a longitudinal study in the West Midlands, United Kingdom

Supplementary file

Table 1: School environment and policy scoring

Description of variable	Score allocation
**Healthy eating score**	
School has a policy regarding HE	0 = No<br>2 = Yes
HE is promoted in school curriculum	0 = No<br>2 = Yes
School cookery club to promote HE	0 = No<br>2 = Yes
Involve parents in the promotion of HE	0 = No<br>2 = Yes
Provide a range of ways to promote HE	Split data into tertiles<br>0 = < 5<br>1 = 5 ≤ x < 7<br>2 = ≥ 7
Drinking water is provided throughout the day	0 = No<br>2 = Yes
Breakfast club is available for students	0 = No<br>2 = Yes
Food available at break deemed as healthy	0 = No†<br>2 = Yes†
Proportion of children choosing to eat school lunches	Split data into tertiles<br>0 = < 50%<br>1 = 50% ≤ x < 60%<br>2 = ≥ 60% of children
Governors support HE in school *	0.33 = Weakly support<br>0.67 = Support<br>1.00 = Strongly support
The Senior Leadership Team support HE in school *	0.33 = Weakly support<br>0.67 = Support<br>1.00 = Strongly support
Staff support HE in school *	0.33 = Weakly support<br>0.67 = Support<br>1.00 = Strongly support
School council support HE in school *	0.33 = Weakly support<br>0.67 = Support<br>1.00 = Strongly support
Pupils support HE in school *	0.33 = Weakly support<br>0.67 = Support<br>1.00 = Strongly support
Parents support HE in school *	0.33 = Weakly support<br>0.67 = Support<br>1.00 = Strongly support
**Physical activity score**	
School has a policy regarding PA	0 = No<br>2 = Yes
School has a PA coordinator	0 = No<br>2 = Yes
Staff are trained regarding PA	0 = No<br>2 = Yes

For peer review only - http://bmjopen.bmj.com/site/about/guidelines.xhtml

**Description of variable**	**Score allocation**
School has sport partnerships and community links	0 = No<br>2 = Yes
PA is promoted in the school curriculum	0 = No<br>2 = Yes
Involve parents in the promotion of PA	0 = No<br>2 = Yes
Have a walk to school campaign	0 = No<br>2 = Yes
Provide a range of ways to promote PA	Split data into tertiles<br>0 = < 5<br>1 = 5 ≤ x < 7<br>2 = ≥ 7
PE is taught by a specialist	0 = no<br>2 = yes
Range of activities offered in PE meet Department of Education recommendations	0 = no<br>2 = yes
Schools allocating two or more hours to PE per week	0 = for none of the school<br>1 = for some of the years<br>2 = for the whole school
Offer a variety of PA opportunities, other than PE	Split data into tertiles<br>0 = 0<br>1 = 1<br>2 = ≥ 2
Have a range of play equipment to be used	Split data into tertiles<br>0 = < 5<br>1 = 5<br>2 = ≥ 6
Provide a range of physically active/ sports clubs	Split data into tertiles<br>0 = < 4<br>1 = 4 ≤ x < 6<br>2 = ≥ 6
Governors support PA in school *	0.33 = Weakly support<br>0.67 = Support<br>1.00 = Strongly support
The Senior Leadership Team support PA in school *	0.33 = Weakly support<br>0.67 = Support<br>1.00 = Strongly support
Staff support PA in school *	0.33 = Weakly support<br>0.67 = Support<br>1.00 = Strongly support
School council support PA in school *	0.33 = Weakly support<br>0.67 = Support<br>1.00 = Strongly support
Pupils support PA in school *	0.33 = Weakly support<br>0.67 = Support<br>1.00 = Strongly support
Parents support PA in school *	0.33 = Weakly support<br>0.67 = Support<br>1.00 = Strongly support

* Given a maximum score of 1, to ensure that level of support by respective groups did not account for too great a proportion of overall score.
† No = Any food other than fruit, vegetables, milk and juice. Yes = Fruit, vegetables, milk and juice
HE, healthy eating. PA, physical activity. PE, physical education.

For peer review only

Table 2: Multilevel models developed for body mass index z-score at the three study time points: all fixed effect covariates are presented

	Baseline		Follow-up one		Follow-up two	
	**B (95% CI)**	**p-value**	**B (95% CI)**	**p-value**	**B (95% CI)**	**p-value**
**Trial arm**	N/A	N/A	0.07 (-0.05, 0.19)	0.24	0.03 (-0.07, 0.14)	0.55
**Baseline zBMI**	N/A	N/A	0.95 (0.93, 0.98)	< 0.01	0.92 (0.89, 0.96)	< 0.01
**Index of Multiple Deprivation score**	0.004 (-0.001, 0.008)	0.09	<0.001 (-0.003, 0.002)	0.74	0.0004 (-0.003, 0.003)	0.80
**Female (reference male)**	-0.08 (-0.22, 0.05)	0.23	-0.11 (-0.18, -0.05)	< 0.01	-0.16 (-0.24, -0.07)	< 0.01
**Ethnicity (reference White)**						
**Asian**	-0.27 (-0.44, -0.10)	< 0.01	0.10 (0.01, 0.20)	0.03	0.16 (0.05, 0.28)	0.01
**African Caribbean**	0.38 (0.11, 0.65)	0.01	0.08 (-0.06, 0.22)	0.29	0.01 (-0.18, 0.19)	0.96
**Other**	-0.02 (-0.23, 0.18)	0.83	0.01 (-0.09, 0.12)	0.78	0.04 (-0.10, 0.17)	0.61
**Age**	0.03 (-0.19, 0.25)	0.76	-0.02 (-0.12, 0.09)	0.73	0.02 (-0.12, 0.16)	0.80
**School environment category (reference = minimal health focus)**						
**PA focus**	-0.06 (-0.25, 0.14)	0.57	-0.06 (-0.22, 0.10)	0.48	-0.17 (-0.31, -0.03)	0.02
**HE focus**	-0.09 (-0.28, 0.09)	0.33	-0.02 (-0.18, 0.15)	0.86	-0.02 (-0.16, 0.13)	0.80
**Complete health focus**	0.04 (-0.14, 0.23)	0.64	0.07 (-0.09, 0.23)	0.41	-0.11 (-0.25. 0.03)	0.14

95% CI, 95% confidence interval. N/A, not applicable. zBMI, body mass index z-score. PA, physical activity. HE, healthy eating.

For peer review only

Table 3: Multilevel models developed for waist circumference z-score at the three study time points: all fixed effect covariates are presented

	Baseline		Follow-up one		Follow-up two	
	**B (95% CI)**	**p-value**	**B (95% CI)**	**p-value**	**B (95% CI)**	**p-value**
**Trial arm**	N/A	N/A	-0.12 (-0.31, 0.07)	0.23	-0.09 (-0.25, 0.07)	0.27
**Baseline zWC**	N/A	N/A	0.90 (0.86, 0.94)	< 0.01	0.85 (0.80, 0.89)	< 0.01
**Index of Multiple Deprivation score**	0.002 (-0.003, 0.007)	0.35	-0.001 (-0.004, 0.003)	0.76	0.001 (-0.003, 0.005)	0.48
**Female (reference male)**	0.07 (-0.07, 0.21)	0.32	-0.06 (-0.15, 0.03)	0.17	0.11 (0.01, 0.21)	0.04
**Ethnicity (reference White)**						
**Asian**	-0.20 (-0.39, -0.01)	0.04	0.19 (0.06, 0.32)	< 0.01	0.21 (0.07, 0.36)	< 0.01
**African Caribbean**	0.33 (0.04, 0.62)	0.03	0.02 (-0.18, 0.21)	0.88	0.07 (-0.17, 0.31)	0.57
**Other**	0.004 (-0.21, 0.22)	0.97	0.06 (-0.08, 0.20)	0.41	0.05 (-0.18, 0.21)	0.58
**Age**	0.05 (-0.18, 0.29)	0.65	-0.06 (-0.21, 0.09)	0.44	0.05 (-0.13, 0.22)	0.58
**School environment category (reference = minimal health focus)**						
**PA focus**	-0.10 (-0.36, 0.16)	0.44	0.04 (-0.22, 0.30)	0.75	-0.15 (-0.36, 0.06)	0.16
**HE focus**	-0.24 (-0.48, 0.01)	0.06	0.19 (-0.07, 0.45)	0.14	0.07 (-0.15, 0.29)	0.55
**Complete health focus**	0.10 (-0.14, 0.34)	0.40	-0.01 (-0.26, 0.24)	0.95	-0.18 (-0.38, 0.03)	0.09

95% CI, 95% confidence interval. N/A, not applicable. zWC, waist circumference z-score. PA, physical activity. HE, healthy eating.

For peer review only

Table 4: Multilevel models developed for dietary total energy intake at the three study time points: all fixed effect covariates are presented

	Baseline		Follow-up one		Follow-up two	
	**B (95% CI)**	**p-value**	**B (95% CI)**	**p-value**	**B (95% CI**	**p-value**
**Trial arm**	N/A	N/A	18.5 (-364.3, 401.4)	0.92	71.4 (-431.0, 573.7)	0.78
**Baseline dietary total energy intake (kJ/ 24 hours)**	N/A	N/A	0.02 (0.01, 0.06)	0.19	0.06 (0.02, 0.11)	< 0.01
**Index of Multiple Deprivation score**	13.7 (-5.1, 32.5)	0.15	2.7 (-7.6, 13.0)	0.61	10.1 (-2.7, 22.8)	0.12
**Female (reference male)**	-586.9 (-1028.2, -145.6)	0.01	-199.7 (-484.2, 84.8)	0.17	-38.9 (-376.8, 298.9)	0.82
**Ethnicity (reference White)**						
**Asian**	-38.4 (-699.0, 622.3)	0.91	-423.5 (-803.7, -43.2)	0.03	304.4 (-153.3, 762.0)	0.19
**African Caribbean**	439.2 (-518.6, 1397.0)	0.37	97.2 (-520.6, 715.1)	0.76	43.9 (-740.1, 827.9)	0.91
**Other**	319.6 (-382.5, 1021.7)	0.37	-505.4 (-944.4, -66.3)	0.02	-120.8 (-654.6, 412.9)	0.66
**Age**	621.5 (-119.3, 1362.3)	0.10	423.3 (-49.1, 895.6)	0.08	353.6 (-208.3, 915.6)	0.22
**School environment category (reference = minimal health focus)**						
**PA focus**	-903.5 (-2402.7, 595.6)	0.24	433.7 (-70.5, 937.9)	0.09	33.2 (-614.5, 681.0)	0.92
**HE focus**	-847.8 (-2255.8, 560.3)	0.24	-63.7 (-598.0, 470.5)	0.82	260.8 (-443.1, 964.7)	0.47
**Complete health focus**	-399.3 (-1801.3, 1002.6)	0.58	32.8 (-464.7, 530.3)	0.90	171.3 (-466.4, 809.1)	0.60

95% CI, 95% confidence interval. N/A, not applicable. PA, physical activity. HE, healthy eating.

For peer review only

Table 5: Multilevel models developed for fruit and vegetable intake at the three study time points: all fixed effect covariates are presented

	Baseline		Follow-up one		Follow-up two	
	B (95% CI)	p-value	B (95% CI)	p-value	B (95% CI)	p-value
**Trial arm**	N/A	N/A	9.6 (-20.5, 39.6)	0.53	-5.5 (-50.3, 39.2)	0.81
**Baseline fruit and vegetable intake (g/ 24 hours)**	N/A	N/A	0.17 (0.12, 0.22)	< 0.01	0.08 (0.02, 0.15)	0.01
**Index of Multiple Deprivation score**	0.31 (-0.72, 1.4)	0.55	-0.58 (-1.4, 0.20)	0.15	-0.66 (-1.7, 0.43)	0.24
**Female (reference male)**	11.8 (-13.0, 36.7)	0.35	36.9 (15.7, 58.1)	< 0.01	12.4 (-15.5, 40.4)	0.38
**Ethnicity (reference White)**						
**Asian**	17.0 (-19.9, 53.8)	0.37	9.6 (-19.1, 38.2)	0.51	46.5 (7.9, 85.0)	0.02
**African Caribbean**	10.8 (-43.0, 64.6)	0.69	64.8 (18.6, 111.0)	0.01	33.2 (-32.1, 98.4)	0.32
**Other**	41.6 (2.2, 81.0)	0.04	26.7 (-6.2, 59.6)	0.11	60.6 (16.1, 105.1)	0.01
**Age**	38.4 (-3.4, 80.1)	0.07	31.5 (-3.7, 66.7)	0.08	16.2 (-30.4, 62.7)	0.50
**School environment category (reference = minimal health focus)**						
**PA focus**	-57.8 (-135.5, 19.8)	0.14	11.8 (-27.9, 51.5)	0.56	-16.0 (-73.9, 41.9)	0.59
**HE focus**	-31.6 (-104.7, 41.4)	0.40	3.0 (-38.6, 44.7)	0.89	-4.4 (-66.9, 58.0)	0.89
**Complete health focus**	-7.3 (-80.0, 65.4)	0.84	27.1 (-11.9, 66.0)	0.17	8.6 (-48.1, 65.3)	0.77

95% CI, 95% confidence interval. N/A, not applicable. PA, physical activity. HE, healthy eating.

For peer review only - http://bmjopen.bmj.com/site/about/guidelines.xhtml

Table 6: Multilevel models developed for physical activity energy expenditure at the three study time points: all fixed effect covariates are presented

	Baseline		Follow-up one		Follow-up two	
	B (95% CI)	p-value	B (95% CI)	p-value	B (95% CI)	p-value
**Trial arm**	N/A	N/A	0.25 (-4.6, 5.1)	0.92	-1.7 (-6.0, 2.6)	0.44
**Baseline PAEE (kJ/kg body weight/ 24 hours)**	N/A	N/A	0.53 (0.45, 0.60)	< 0.01	0.43 (0.36, 0.51)	< 0.01
**Index of Multiple Deprivation score**	0.09 (0.01, 0.19)	0.09	-0.10 (-0.22, 0.02)	0.09	-0.02 (-0.13, 0.10)	0.79
**Female (reference male)**	-14.1 (-16.9, -11.2)	< 0.01	-4.9 (-8.2, -1.6)	< 0.01	-7.0 (-10.4, -3.5)	< 0.01
**Ethnicity (reference White)**						
**Asian**	-3.8 (-7.7, 0.13)	0.06	-3.7 (-8.0, 0.62)	0.09	-7.9 (-12.1, -3.6)	< 0.01
**African Caribbean**	-17.8 (-23.8, -11.8)	< 0.01	-6.9 (-14.0, 0.18)	0.06	-12.8 (-21.0, -4.6)	< 0.01
**Other**	-4.1 (-8.4, 0.24)	0.06	-2.4 (-7.0, 2.3)	0.32	-4.4 (-9.4, 0.73)	0.09
**Age**	0.57 (-4.2, 5.3)	0.81	2.0 (-3.2, 7.3)	0.46	-6.6 (-12.1, -1.1)	0.02
**School environment category (reference = minimal health focus)**						
**PA focus**	-6.0 (-11.7, -0.28)	0.04	-0.79 (-7.2, 5.7)	0.81	1.9 (-3.8, 7.6)	0.51
**HE focus**	-6.3 (-11.8, -0.86)	0.02	3.8 (-3.1, 10.7)	0.28	3.5 (-2.7, 9.7)	0.27
**Complete health focus**	-6.3 (-11.6, -1.0)	0.02	-2.6 (-8.8, 3.6)	0.42	5.3 (0.04, 10.6)	0.05

95% CI, 95% confidence interval. N/A, not applicable. PAEE, physical activity energy expenditure. PA, physical activity. HE, healthy eating.

For peer review only

Table 7: Multilevel models developed for estimated time spent in moderate to vigorous physical activity at the three study time points: all fixed effect covariates are presented

	Baseline		Follow-up one		Follow-up two	
	**B (95% CI)**	**p-value**	**B (95% CI)**	**p-value**	**B (95% CI)**	**p-value**
**Trial arm**	N/A	N/A	0.57 (-13.5, 14.6)	0.94	3.8 (-7.1, 14.7)	0.49
**Baseline estimated time spent in MVPA (min/ 24 hours)**	N/A	N/A	0.21 (0.11, 0.31)	< 0.01	0.13 (0.06, 0.20)	< 0.01
**Index of Multiple Deprivation score**	0.26 (0.03, 0.50)	0.03	-0.18 (-0.52, 0.15)	0.28	0.04 (-0.29, 0.20)	0.72
**Female (reference male)**	-13.6 (-19.6, -7.6)	< 0.01	-13.6 (-22.4, -4.7)	< 0.01	-13.6 (-19.6, -7.6)	< 0.01
**Ethnicity (reference White)**						
**Asian**	-3.0 (-11.6, 5.5)	0.49	-5.8 (-18.0, 6.4)	0.35	-12.5 (-20.8, -4.2)	< 0.01
**African Caribbean**	2.6 (-10.2, 15.5)	0.69	-3.1 (-23.1, 17.0)	0.77	-7.3 (-22.3, 7.6)	0.34
**Other**	5.8 (-3.4, 15.1)	0.22	-3.6 (-16.8, 9.6)	0.59	-3.4 (-13.0, 6.1)	0.48
**Age**	-0.62 (-10.7, 9.4)	0.90	8.4 (-6.4, 23.2)	0.27	-9.1 (-19.1, 1.0)	0.08
**School environment category (reference = minimal health focus)**						
**PA focus**	-4.2 (-18.5, 10.1)	0.57	-3.7 (-22.7, 15.2)	0.70	3.8 (-10.5, 18.2)	0.60
**HE focus**	-13.2 (-26.8, 0.47)	0.06	-0.80 (-20.9, 19.3)	0.94	0.78 (-14.5, 16.1)	0.92
**Complete health focus**	-14.1 (-27.4, -0.75)	0.04	-6.4 (-24.7, 11.8)	0.49	7.2 (-6.4, 20.9)	0.30

95% CI, 95% confidence interval. N/A, not applicable. MVPA, moderate to vigorous physical activity. PA, physical activity. HE, healthy eating.

For peer review only

Table 8: Repeated measures models for outcomes of body mass index z-score, waist circumference z-score, dietary total energy intake, fruit and vegetable intake, physical activity energy expenditure and time spent in moderate to vigorous physical activity: all fixed covariates are presented

	**Outcome**											
	**zBMI**		**zWC**		**Dietary total energy intake (kJ/24 hrs)**		**Fruit and vegetable intake (g/24 hrs)**		**PAEE (kJ/kg body weight/24 hrs)**		**Estimated time in MVPA (min/24 hrs)**	
	**B (95% CI)**	**p-value**	**B (95% CI)**	**p-value**	**B (95% CI)**	**p-value**	**B (95% CI)**	**p-value**	**B (95% CI)**	**p-value**	**B (95% CI)**	**p-value**
**Trial arm**	-0.04 (-0.19, 0.10)	0.55	-0.11 (-0.28, 0.05)	0.17	322.7 (-120.8, 766.2)	0.15	20.1 (-11.4, 51.6)	0.21	-1.9 (-5.1, 1.4)	0.26	0.27 (-7.5, 8.1)	0.95
**Time point (reference baseline)**												
**Follow-up 1**	0.11 (0.08, 0.15)	<0.01	0.25 (0.20, 0.29)	<0.01	-229.2 (-491.6, 33.2)	0.09	-52.5 (-68.4, -36.6)	<0.01	-2.7 (-4.4, -1.0)	<0.01	10.4 (6.0, 14.8)	<0.01
**Follow-up 2**	0.19 (0.15, 0.22)	<0.01	0.30 (0.25, 0.35)	<0.01	445.1 (174.9, 715.2)	<0.01	-29.5 (-45.9, -13.1)	<0.01	-15.1 (-16.9, -13.3)	<0.01	-16.5 (-21.1, -11.8)	<0.01
**Index of Multiple Deprivation score**	0.004 (-0.001, 0.01)	0.12	0.003 (-0.002, 0.01)	0.26	10.6 (1.2, 19.9)	0.02	0.05 (-0.62, 0.71)	0.89	0.0002 (-0.8, 0.8)	0.99	0.04 (-0.14, 0.21)	0.67
**Female (reference male)**	-0.15 (-0.29, -0.01)	0.03	0.06 (-0.07, 0.20)	0.36	-316.1 (-554.0, -87.1)	0.01	20.3 (4.1, 36.5)	0.01	-13.3 (-15.6, -11.0)	<0.01	-14.3 (-18.7, -9.9)	<0.01
**Ethnicity (reference White)**												
**Asian**	-0.19 (-0.36, -0.02)	0.03	-0.09 (-0.26, 0.09)	0.34	-18.8 (-349.0, 311.5)	0.91	19.9 (-3.6, 43.3)	0.10	-6.7 (-9.8, -3.5)	<0.01	-8.1 (-14.3, -1.9)	0.01
**African Caribbean**	0.38 (0.11, 0.65)	0.01	0.31 (0.03, 0.59)	0.03	198.2 (-299.7, 696.0)	0.44	26.1 (-8.9, 61.0)	0.14	-16.9 (-21.9, -12.0)	<0.01	0.80 (-8.8, 10.4)	0.87
**Other**	-0.004 (-0.21, 0.20)	0.97	0.02 (-0.19, 0.23)	0.87	-38.9 (-401.4, 323.5)	0.83	41.4 (15.8, 67.1)	<0.01	-5.7 (-9.3, -2.2)	<0.01	0.52 (-6.3, 7.3)	0.88
**Age**	0.03 (-0.19, 0.25)	0.49	0.11 (-0.12, 0.33)	0.35	529.3 (148.7, 909.9)	0.01	37.9 (11.0, 64.9)	0.01	-0.70 (-4.6, 3.2)	0.73	-1.8 (-9.2, 5.5)	0.62
**School environment category (reference = minimal health focus)**												

For peer review only

	Outcome											
	**zBMI**		**zWC**		**Dietary total energy intake (kJ/24 hrs)**		**Fruit and vegetable intake (g/24 hrs)**		**PAEE (kJ/kg body weight/24 hrs)**		**Estimated time in MVPA (min/24 hrs)**	
	**B (95% CI)**	**p-value**	**B (95% CI)**	**p-value**	**B (95% CI)**	**p-value**	**B (95% CI)**	**p-value**	**B (95% CI)**	**p-value**	**B (95% CI)**	**p-value**
**PA focus**	-0.10 (-0.29, 0.10)	0.32	-0.11 (-0.32, 0.11)	0.33	-220.9 (-815.1, 373.4)	0.47	-22.6 (-64.9, 19.6)	0.29	-2.8 (-7.2, 1.6)	0.21	-1.4 (-11.9, 9.1)	0.80
**HE focus**	-0.07 (-0.27, 0.13)	0.47	-0.14 (-0.37, 0.08)	0.21	-483.8 (-1087.4, 119.9)	0.12	-21.6 (-64.4, 21.2)	0.32	-0.83 (-5.3, 3.7)	0.72	-5.0 (-15.7, 5.7)	0.36
**Complete health focus**	0.07 (-0.12, 0.26)	0.49	0.07 (-0.14, 0.28)	0.52	-200.0 (-773.9, 373.8)	0.49	5.16 (-35.6, 45.9)	0.80	-3.0 (-7.2, 1.2)	0.16	-6.4 (-16.5, 3.7)	0.21

zBMI, body mass index z-score. zWC, waist circumference z-score. PAEE, physical activity energy expenditure. MVPA, moderate to vigorous physical activity. 95% CI, 95% confidence interval. PAEE, physical activity energy expenditure. PA, physical activity. HE, healthy eating.

For peer review only

STROBE Statement—checklist of items that should be included in reports of observational studies

	Item No	Recommendation	Page No
**Title and abstract**	1	(*a*) Indicate the study's design with a commonly used term in the title or the abstract	1-2
		(*b*) Provide in the abstract an informative and balanced summary of what was done and what was found	2-3
**Introduction**			
Background/rationale	2	Explain the scientific background and rationale for the investigation being reported	4-5
Objectives	3	State specific objectives, including any prespecified hypotheses	5
**Methods**			
Study design	4	Present key elements of study design early in the paper	6
Setting	5	Describe the setting, locations, and relevant dates, including periods of recruitment, exposure, follow-up, and data collection	6-7
Participants	6	(*a*) *Cohort study*—Give the eligibility criteria, and the sources and methods of selection of participants. Describe methods of follow-up<br>*Case-control study*—Give the eligibility criteria, and the sources and methods of case ascertainment and control selection. Give the rationale for the choice of cases and controls<br>*Cross-sectional study*—Give the eligibility criteria, and the sources and methods of selection of participants	6-7
		(*b*) *Cohort study*—For matched studies, give matching criteria and number of exposed and unexposed<br>*Case-control study*—For matched studies, give matching criteria and the number of controls per case	NA
Variables	7	Clearly define all outcomes, exposures, predictors, potential confounders, and effect modifiers. Give diagnostic criteria, if applicable	7-8
Data sources/ measurement	8*	For each variable of interest, give sources of data and details of methods of assessment (measurement). Describe comparability of assessment methods if there is more than one group	7-8
Bias	9	Describe any efforts to address potential sources of bias	6-7, 11-12
Study size	10	Explain how the study size was arrived at	6-7
Quantitative variables	11	Explain how quantitative variables were handled in the analyses. If applicable, describe which groupings were chosen and why	7-12
Statistical methods	12	(*a*) Describe all statistical methods, including those used to control for confounding	10-13
		(*b*) Describe any methods used to examine subgroups and interactions	NA
		(*c*) Explain how missing data were addressed	13
		(*d*) *Cohort study*—If applicable, explain how loss to follow-up was addressed<br>*Case-control study*—If applicable, explain how matching of cases and controls was addressed<br>*Cross-sectional study*—If applicable, describe analytical methods taking account of sampling strategy	13, 25
		(*e*) Describe any sensitivity analyses	NA

For peer review only - http://bmjopen.bmj.com/site/about/guidelines.xhtml

Continued on next page

For peer review only

**Results**			
Participants	13*	(a) Report numbers of individuals at each stage of study—eg numbers potentially eligible, examined for eligibility, confirmed eligible, included in the study, completing follow-up, and analysed	7, 13
		(b) Give reasons for non-participation at each stage	14
		(c) Consider use of a flow diagram	Flow diagram for trial is reported in main trial paper
Descriptive data	14*	(a) Give characteristics of study participants (eg demographic, clinical, social) and information on exposures and potential confounders	13-14
		(b) Indicate number of participants with missing data for each variable of interest	14, 16 (Tables 2 &3)
		(c) *Cohort study*—Summarise follow-up time (eg, average and total amount)	7
Outcome data	15*	*Cohort study*—Report numbers of outcome events or summary measures over time	16 (Table 3)
		Case-control study—Report numbers in each exposure category, or summary measures of exposure	
		Cross-sectional study—Report numbers of outcome events or summary measures	
Main results	16	(*a*) Give unadjusted estimates and, if applicable, confounder-adjusted estimates and their precision (eg, 95% confidence interval). Make clear which confounders were adjusted for and why they were included	20-23
		(*b*) Report category boundaries when continuous variables were categorized	11-12
		(*c*) If relevant, consider translating estimates of relative risk into absolute risk for a meaningful time period	NA
Other analyses	17	Report other analyses done—eg analyses of subgroups and interactions, and sensitivity analyses	NA
**Discussion**			
Key results	18	Summarise key results with reference to study objectives	24
Limitations	19	Discuss limitations of the study, taking into account sources of potential bias or imprecision. Discuss both direction and magnitude of any potential bias	25-27
Interpretation	20	Give a cautious overall interpretation of results considering objectives, limitations, multiplicity of analyses, results from similar studies, and other relevant evidence	29-30
Generalisability	21	Discuss the generalisability (external validity) of the study results	24-25
**Other information**			
Funding	22	Give the source of funding and the role of the funders for the present study and, if applicable, for the original study on which the present article is based	32

*Give information separately for cases and controls in case-control studies and, if applicable, for exposed and unexposed groups in cohort and cross-sectional studies.

For peer review only

Note: An Explanation and Elaboration article discusses each checklist item and gives methodological background and published examples of transparent reporting. The STROBE checklist is best used in conjunction with this article (freely available on the Web sites of PLoS Medicine at http://www.plosmedicine.org/, Annals of Internal Medicine at http://www.annals.org/, and Epidemiology at http://www.epidem.com/). Information on the STROBE Initiative is available at www.strobe-statement.org.

For peer review only

For peer review only - http://bmjopen.bmj.com/site/about/guidelines.xhtml
